# An efficient synthesis of 4*H*-pyrano quinolinone derivatives catalysed by a versatile organocatalyst tetra-*n*-butylammonium fluoride and their pharmacological screening

**DOI:** 10.1098/rsos.170764

**Published:** 2017-11-29

**Authors:** Pratibha Prasad, Pratik G. Shobhashana, Manish P. Patel

**Affiliations:** Department of Chemistry, Sardar Patel University, Vallabh Vidyanagar, Gujarat 388120, India

**Keywords:** quinoline, indole, tetra-*n*-butylammonium fluoride (TBAF), anti-microbial activity

## Abstract

A new series of indole-based pyranoquinoline derivatives **P_1–24_** has been synthesized by a one-pot cyclocondensation reaction of 2-(4-substituted)phenyl-*N*-allyl-indole-3-carbaldehydes **1a–d**; active methylenes **2a–c**; and 4-hydroxy-1-substituted quinolin-2(1*H*)-one **3a–b** catalysed by an organocatalyst tetra-*n*-butylammonium fluoride (TBAF) in aqueous ethanol. The easy experimental procedure of the reaction leads to excellent yields of pyranoquinoline derivatives. All the compounds were screened against a representative panel of bacteria and fungi. Some of the compounds are found to be equipotent or more potent than standard drugs as evident from the structural activity relationship study.

## 1. Introduction

**Figure 1.**
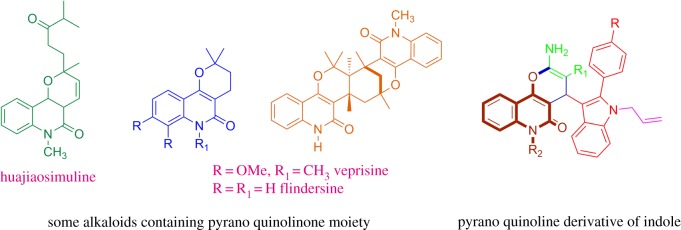
Some alkaloids containing pyrano quinolinone moiety and repurposed compound similarity.

Quinolines and indole are privileged heterocyclic ring moieties existing in a number of pharmacologically active natural or synthetic products, and have been used as templates for the synthesis of many drugs prescribed for a lot of pathologies. Quinolines are an important group of compounds of both natural and synthetic origins; especially those with a pyranoquinoline ring system are of considerable interest as it is a core structure and constitutes the basic skeleton of a number of alkaloids [1], such as flindersine, oricine and verprisine, which are generally seen in the plant family Rutaceae [2] (figure 1). These derivatives exhibit a wide range of biological activities [3–12] such as anti-allergic, anti-bacterial, anti-microbial, anti-coagulant, anti-tumour, anti-hypertensive, anti-algal and anti-inflammatory activities. In addition to these bioactivities, some of them show cancer cell growth inhibitory activity and have been found to be potential anti-cancer agents [13]. The pyranoquinolinone moiety is also found in many alkaloids. These are also useful intermediates for the construction of azo dyestuffs [14].

Contrariwise, the indole ring system is also probably the most pervasive heterocycle in nature. Owing to the great structural diversity and great significance of biologically active indoles [15], these derivatives have been a topic of extensive research interest in existing heterocyclic and medicinal chemistry, as they are an essential part of the amino acid tryptophan and the neurotransmitter serotonin. The indole scaffold is also found in a manifold of naturally occurring plant-based alkaloids [16]. *N*-1 and *C*-3-substituted indole derivatives show various pharmacological properties and are widely recognized to have anti-inflammatory, anti-cancer, anti-nociceptive and anti-psychotic, anti-fungal, anti-migraine, anti-parasitic, anti-tuberculosis and anti-malarial drug activities [17].

**Scheme 1.**
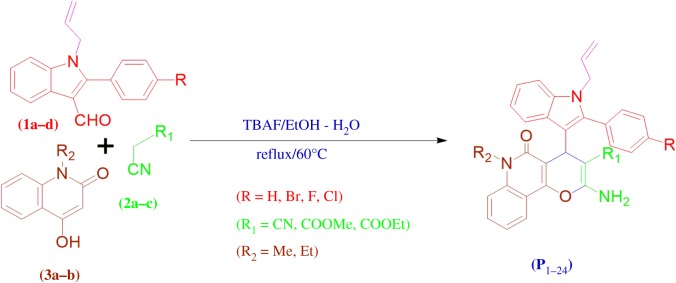
Synthetic pathway for the synthesis of pyrano quinoline derivatives of compounds **1a–d.**

The developments of multicomponent reactions (MCRs) have attracted much attention from the vantage point of combinatorial and medicinal chemistry. Among available methods, intramolecular cyclization via multicomponent reaction is an efficient protocol for the synthesis of new pharmacologically active heterocycles [18]. A literature survey manifests that using this one-pot, three-component approach, considerable efforts have been focused on the design and development of environmentally friendly and less expensive procedures for the generation of libraries of heterocyclic compounds employing various catalysts [19–26]. Therefore, the molecular manipulation of the promising lead involves an idea to merge the separate pharmacophoric groups of analogous activity into one compound, hence making structural changes in the biological activity. Thus, in continuance to the aforementioned facts, and as a prolongation of our investigation on the synthesis of biologically active heterocyclic compounds [27–30], we attempted to report an efficient synthesis of pyranoquinoline derivatives of the substituted 2-phenyl-*N-*allyl-indole scaffold, which are obtained through three-component reactions of substituted indole aldehydes **1**, different active methylene compounds **2** and 4-hydroxy-1-alkylquinolin-2(1*H*)-one **3** catalysed by tetra butyl ammonium fluoride (TBAF) in EtOH/H_2_O (2 : 8) (scheme 1). Also, these were biologically evaluated.

As tetra-*n*-butylammonium fluoride (TBAF) is a quaternary ammonium salt, it is widely renowned as a convenient, organic soluble source of naked fluoride ions and can be used as a phase-transfer catalyst and as a mild base. The TBAF is highly soluble in organic solvents, and weakly nucleophilic. Over the past years, TBAF has been widely used for most fluoride-assisted reactions [31], desilylation [32,33], deprotection of silyl groups [34], trifluoromethylation, fluorination [35,36], and a variety of base-catalysed reactions such as elimination, alkylation, Michael addition and aldol condensation [37–39]. The constitutions of all the products were confirmed using ^1^H NMR, ^13^C NMR, FTIR and elemental analysis. All synthesized compounds were screened for *in vitro* anti-microbial activity against a representative panel of bacteria and fungi using the broth microdilution minimum inhibitory concentration (MIC) method.

## 2. Material and methods

### 2.1. General procedures

Required acetophenones, phenyl hydrazine, polyphosphoric acid, anthranilic acid, diethyl/dimethyl sulphate and phosphorus oxychloride were obtained commercially. Moreover, malanonitrile, ethyl cyanoacetate and methyl cyanoacetate were obtained from Sigma-Aldrich and were used without further purification. Solvents were purified and dried before being used. The required substituted 2-phenyl-*N*-allyl-indole-3-carbaldehyde **1a–d** was prepared by the Vilsmeier–Haack reaction according to the procedure in the literature [40]. All melting points were taken in open capillaries and are uncorrected. Thin-layer chromatography (TLC, on aluminium plates precoated with silica gel, 60F_254_, 0.25 mm thickness) (Merck, Darmstadt, Germany) was used for monitoring the progress of all reactions, and purity and homogeneity of the synthesized compounds; eluent-hexane : ethyl acetate (5 : 5). UV radiation and/or iodine were used as the visualizing agents. Elemental analysis (% C, H, N) was carried out with the Perkin-Elmer 2400 series-II elemental analyser (PerkinElmer, USA), and all compounds are within ±0.4% of the theoretical value. The IR spectra were recorded in KBr on a PerkinElmer Spectrum GX FT-IR Spectrophotometer (PerkinElmer, USA) and only the characteristic peaks are reported in cm^−1^. ^1^H NMR and ^13^C NMR spectra were recorded in DMSO-*d_6_* on a Bruker Advance 400F (MHz) spectrometer (Bruker Scientific Corporation Ltd., Switzerland) using solvent peak as the internal standard at 400 and 100 MHz, respectively. Chemical shifts are reported in parts per million (ppm). Mass spectra were scanned on a Shimadzu LCMS 2010 spectrometer (Shimadzu, Tokyo, Japan).

### 2.2. General procedure for synthesis of 4-hydroxy-1-substituted quinolin-2(1*H*)-one **3a–b** [41]

Anthranilic acid (0.1 mmol) was dissolved in 15 ml of 5% NaOH. To this clear solution dimethylsulfate/diethylsulfate (0.2 mmol) was added and the mixture was stirred for 1 h. The separated solid was filtered out and washed with cold water and dried. It was recrystallized with ethanol. After that, *N*-methyl/ethyl anthranilic acid (0.01 mol) was dissolved in 50 ml of acetic acid, and 50 ml of acetic anhydride was added. The reaction mixture was heated at 120°C for 6.0 h and poured into ice. After basification with NaOH, the residue was filtered. The filtrate was acidified with HCl and cooled. The solid precipitate was filtered out, washed with benzene and dried, to give the desired product, which was used without further purification.

### 2.3. General procedure for the synthesis of compounds **P_1–24_**

Substituted 2-phenyl-*N*-allyl-indole-3-carbaldehyde **1a–d** (1 mmol), malononitrile/methyl cyanoacetate/ethyl cyanoacetate **2a–c** (1 mmol), 4-hydroxy-1-substituted quinolin-2(1*H*)-one **3a–b** (1 mmol) and a catalytic TBAF (20 mol%) in water : ethanol (8 : 2) were taken. The reaction mixture was heated under reflux for 3–3.5 h and the progress of the reaction was monitored by TLC. After the completion of reaction (as evidenced by TLC), the reaction mixture was cooled to room temperature and stirred magnetically for a further 20 min. The solid mass that separated was collected by filtration, washed well with ethanol (10 ml) and purified by leaching in equal volume ratio of chloroform and methanol (15 ml) to obtain pure solid sample **P_1–24_**.

Initially, 2-phenyl-1-allyl-3-indole-carbaldehyde (1 mmol), malononitrile (1 mmol) and 4-hydroxy-1-methylquinolin-2(1*H*)-one (1 mmol) were refluxed in H_2_O for 10.0 h without catalyst. Only Knoevenagel intermediate was formed with malononitrile, while no reaction progress was observed in the case of water as a solvent. TLC of the above reaction mixture showed the non-consumption of 4-hydroxy-1-methylquinolin-2(1*H*)-one. Successively, we tried to explore an effective system for MCRs by screening other fluoride sources under the same conditions. It was found that the fluoride ion of TBAF is more reactive than other fluoride sources (entries 6–9, table 1).

**Table 1.** The effects of different catalytic amounts of TBAF and other catalysts based on the % yield of **P_1_** in EtOH + H_2_O (2 : 8) under reflux.

trials no.	catalyst	mol (%)	time (h)	yield (%)
**1**	**TBAF**	**20**	**3**	**90**
2	TBAF	10	3	85
3	TBAF	5	4.5	78
5	TBAF	0	8	trace
6	KF	20	3.5	85
7	CsF	20	3.5	80
8	NH_4_F	20	3.5	78
9	H_2_SiF_6_	20	6	45
10	NaOH	20	4	70
11	K_2_CO_3_	20	4.5	40

The corresponding product was obtained in low yield in the presence of H_2_SiF_6_ because it prefers to afford acidic HF molecular, not basic F^−^ anion [42]. Besides the basic F^−^ anion, the corresponding product was obtained in a 45–70% yield in the presence of NaOH and K_2_CO_3_ (entries 10–11, table 1). Encouraged by this result, to our delight, organocatalyst TBAF provided satisfactory results.

Confident that the method would endure structural diversity, focus then shifted to optimization. We examined different conditions including catalysts and solvents to optimize the reaction. To optimize the catalyst loading, a mixture of 2-phenyl-1-allyl-3-indole-carbaldehyde, malononitrile and 4-hydroxy-1-methylquinolin-2(1*H*)-one was taken for a model reaction carried out under different TBAF percentages (20 mol%, 10 and 5). Higher percentage loading of the catalyst neither increased the yield nor reduced the reaction time. It was observed that 20 mol% loading of the TBAF provided the best result in terms of yield of **P_1_** and time (entries 1–4, table 1).

Finally, the model reaction was examined and optimized using various solvents to obtain the best yield of **P_1_**. To find the most appropriate solvent for TBAF, various polar-aprotic (CH_3_CN, THF and DMF), polar-protic (*n*-BuOH, *i*-PrOH, MeOH, EtOH, H_2_O) and the mixture of (EtOH + H_2_O) solvents were used. Among different reaction mediums, the use of polar-protic solvents in contrast to EtOH led to a significant decrease in yield. However, it was in fact pleasing to note an appreciable increase in the yield of **P_1_** with the choice of water as the solvent, with a drawback that the desired product obtained was a sticky solid. Hence, the relevant reaction condition was confined to the selection of the optimum EtOH : H_2_O proportion, and during these studies we ascertained that the best results were obtained by using EtOH : H_2_O (2 : 8) medium. The reactions usually completed within 3–3.5 h (monitored by TLC). Thus, we preferred to carry out the reactions using EtOH : H_2_O (2 : 8) as an ecofriendly and safe medium. The results are summarized in table 2.

**Table 2.** The effects of different solvents on the % yield of **P_1_** using TBAF (20 mol%) under reflux condition.

trials no.	solvents	yield (%)
1	EtOH + H_2_O (5 : 5)	60
2	EtOH + H_2_O (4 : 6)	65
3	EtOH + H_2_O (3 : 7)	85
**4**	**EtOH + H_**2**_O (2 : 8)**	**90**
5	EtOH + H_2_O (1 : 9)	80
6	H_2_O	85
7	EtOH	75
8	*n*-BuOH	50
9	*i-*PrOH	10
10	MeOH	41
11	THF	80
12	ACN	50
13	DMF	20

## 3. Results and discussion

### 3.1. Chemistry

In this study, a series of 4*H*-pyrano derivatives **P_1_–P_24_** have been synthesized by the one-pot three-component cyclocondensation reaction of substituted 2-(4-substituted) phenyl-*N*-allyl-indole-3-carbaldehyde **1a–d**; active methylenes **2a–c**; and 4hydroxy-*N*-alkyl-2-quinolinone **3a–b** under reflux catalysed by an organocatalyst TBAF. The above mixture under refluxing in water–ethanol mixtures of different ratios gives moderate to good yield, i.e. 50–90%. Presumably, the formation of the pyrano[3,2-*c*]quinoline derivatives may occur via an *in situ* initial formation of the heterylidene nitrile in the presence of TBAF via Knoevenagel condensation between various substituted indole aldehyde **1a–d** and active methylenes **2a–c.** Then, the enolate, which is obtained from the reaction between quinolinone **3a–b** and TBAF, performs the Michael addition to intermediate heterylidenenitrile. This subsequently undergoes enolization followed by intramolecular cyclization reaction and then 1, 3-proton shift to give the products **P_1–24_** exclusively. The structures of all the new synthesized compounds were established by ^1^H NMR, ^13^C NMR, FTIR and elemental analysis, and molecular weights of some selected compounds were confirmed by mass spectrometry. In ^1^H NMR, (DMSO-*d_6_*) spectrum of compound **P_5_** indicated the presence of one singlet peak at *δ* 5.18 ppm of –CH proton, and the disappearance of a singlet from *δ* 10.50 ppm of –CHO clearly confirms the cyclization of Knoevenagel intermediate. The NH_2_ protons of the pyran ring and aromatic protons of **P_5_** resonate as multiplets at *δ* 6.89–8.02 ppm. Further, disappearance of the aromatic singlet at *δ* 11.24 ppm stands for the secondary amine of the indole ring and confirms the *N*-allylation of the corresponding aldehyde. The allylic germinal *cis*, *trans* and vicinal protons resonate as a multiplet at *δ* 4.55–4.70 and another single geminal proton also gives a multiplet at *δ* 5.74–5.81. A triplet peak at *δ* 1.14 and a multiplet *δ* 4.09–4.32 project the *N*-ethylated protons of quinolones, while carboxylate protons show a singlet peak at *δ* 3.15. The IR spectrum of compound **P_5_** exhibited characteristic absorption bands around 3315–3285 cm^−1^and 1680–1692 cm^−1^ for (asym. and sym. stretching) –NH and C = O str., respectively.

### 3.2. Evaluation of anti-microbial activity

The *in vitro* anti-microbial activity was carried out against 24 h old cultures of three bacteria and two fungi by the disc diffusion method [43]. Compounds **P_1–24_** have been tested for their anti-bacterial activity against *Escherichia coli, Salmonella typhi and Vibrio cholerae* as Gram-negative bacteria and *Streptococcus pneumoniae, Bacillus subtilis* and *Clostridium tetani* as Gram-positive bacteria, and anti-fungal activity against *Candida albicans* and *Trichophyton rubrum.* Nutrient agar and potato dextrose were used to culture the bacteria and fungus, respectively. The compounds were tested at 1000 ppm in DMF solution. Ampicillin, chloramphenicol and nyastatin, griseofulvin were used as standards for comparison of anti-bacterial and anti-fungal activities, respectively. Inhibition was recorded by measuring the diameter of the inhibition zone at the end of 24 h for bacteria at 35°C and 48 h for fungus at 28°C. The results are summarized in table 3

**Table 3.** Per cent yield of synthesized compounds **P_1–24_** and their *in vitro* anti-microbial activity (MIC, μg ml^−1^). Italicized values indicate the active compounds; E.C., *Escherichia coli*; S.T., *Salmonella typhi*; V.C., *Vibrio cholerae*; S.P., *Streptococcus pneumoniae*; B.S., *Bacillus subtilis*; C.T., *Clostridium tetani*; C.A., *Candida albicans*; T.R., *Trichophyton rubrum*. MTCC, microbial-type culture collection; ‘—’ indicates not tested.

		Gram-negative bacteria			Gram-positive bacteria			fungi	
		E.C.	S.T.	V.C.	S.P.	B.S.	C.T.	C.A.	T.R.
	yield	MTCC	MTCC	MTCC	MTCC	MTCC	MTCC	MTCC	MTCC
compound	(%)	443	98	3906	1936	441	449	227	297
**P_1_**(R = H, R1 = CN, R2 = CH_3_)	90	250	*100*	125	200	*200*	*200*	*500*	1000
**P_2_** (R = H, R1 = COOMe, R2 = CH_3_)	92	125	200	200	500	500	500	*200*	1000
**P_3_** (R = H, R1 = COOEt, R2 = CH_3_)	86	125	200	250	250	*100*	*200*	1000	*500*
**P_4_** (R = H, R1 = CN, R2 = CH_2_CH_3_)	91	*62.5*	250	250	*100*	*125*	*125*	>1000	>1000
**P_5_** (R = H, R1 = COOMe, R2 = CH_2_CH_3_)	87	200	250	250	200	*100*	*100*	>1000	>1000
**P_6_**(R = H, R1 = COOEt, R2 = CH_2_CH_3_)	91	*100*	*100*	125	*100*	*250*	*250*	*500*	*500*
**P_7_** (R = Br, R1 = CN, R2 = CH_3_)	86	200	*62.5*	*100*	200	*100*	*100*	1000	>1000
**P_8_** (R = Br, R1 = COOMe, R2 = CH_3_)	88	*100*	*100*	250	200	*250*	*250*	1000	>1000
**P_9_** (R = Br, R1 = COOEt, R2 = CH_3_)	90	200	200	200	200	*125*	*100*	*250*	1000
**P_10_** (R = Br, R1 = CN, R2 = CH_2_CH_3_)	92	250	250	200	200	*250*	*250*	*250*	*500*
**P_11_** (R = Br, R1 = COOMe, R2 = CH_2_CH_3_)	85	*100*	*62.5*	250	200	*62.5*	*200*	>1000	>1000
**P_12_** (R = Br, R1 = COOEt, R2 = CH_2_CH_3_)	86	200	125	*100*	*100*	*100*	*200*	1000	>1000
**P_13_** (R = F, R1 = CN, R2 = CH_3_)	82	*100*	*100*	200	200	*200*	*250*	1000	1000
**P_14_** (R = F, R1 = COOMe, R2 = CH_3_)	87	250	250	200	250	*250*	*250*	1000	1000
**P_15_** (R = F, R1 = COOEt, R2 = CH_3_)	90	250	250	250	500	*200*	*200*	*250*	*500*
**P_16_** (R = F, R1 = CN, R2 = CH_2_CH_3_)	78	250	250	125	200	500	500	*100*	>1000
**P_17_** (R = F, R1 = COOMe, R2 = CH_2_CH_3_)	80	125	200	*100*	200	*200*	*200*	*500*	>1000
**P_18_** (R = F, R1 = COOEt, R2 = CH_2_CH_3_)	91	200	*12.5*	500	125	*200*	*125*	*500*	1000
**P_19_** (R = Cl, R1 = CN, R2 = CH_3_)	93	250	*100*	200	200	*125*	*100*	*250*	*500*
**P_20_** (R = Cl, R1 = COOMe, R2 = CH_3_)	85	*62.5*	*100*	500	500	*125*	*100*	1000	>1000
**P_21_** (R = Cl, R1 = COOEt, R2 = CH_3_)	87	*100*	200	200	200	*200*	*62.5*	>1000	>1000
**P_22_** (R = Cl, R1 = CN, R2 = CH_2_CH_3_)	81	250	250	250	*100*	*100*	*200*	>1000	1000
**P_23_** (R = Cl, R1 = COOMe, R2 = CH_2_CH_3_)	82	*100*	250	*100*	*62.5*	*100*	*200*	*500*	1000
**P_24_** (R = Cl, R1 = COOEt, R2 = CH_2_CH_3_)	85	*62.5*	*100*	125	*100*	*100*	*125*	*100*	*500*
ampicillin	—	*100*	*100*	*100*	*100*	*250*	*250*	—	—
chloramphenicol	—	*50*	*50*	*50*	*50*	*50*	*50*	—	—
nystatin	—	—	—	—	—	—	—	*100*	*500*
griseofulvin	—	—	—	—	—	—	—	*500*	*500*

#### 3.2.1. Gram-positive bacteria

Against Gram-positive bacteria *C. tetani*, **P_21_** (MIC 62.5 µg ml^−1^) was found to have outstanding activity; compounds **P_5_, P_7_, P_9_, P_19_, P_20_** (MIC 100 µg ml^−1^) showed significant activity; compounds **P_4_, P_24_** (MIC 125 µg ml^−1^) exhibited moderate activity; while compounds **P_1_, P_3_, P_11_, P_12_, P_15_, P_17_, P_22_, P_23_** (MIC 200 µg ml^−1^) showed better activity; while compounds **P_6_, P_8_, P_10_, P_14_, P_15_** (MIC 250 µg ml^−1^) were found equally potent when compared with ampicillin (MIC 250 µg ml^−1^).

#### 3.2.2. Gram-negative bacteria

Compounds **P_4_, P_20_** and **P_24_** (MIC 62.5 µg ml^−1^) against Gram-negative bacteria *E. coli* and **P_7_, P_11_** (MIC 62.5 µg ml^−1^) against *S. typhi,* displayed excellent potency compared to ampicillin (MIC 100 µg ml^−1^); whereas compounds (MIC 100 µg ml^−1^) **P_6_, P_8_, P_11_, P_13_, P_21_, P_23_** against *E. coli* and **P_1_, P_6_, P_8_, P_13_, P_19_, P_20_, P_24_** against *S. typhi* were equipotent compared to ampicillin (MIC 100 µg ml^−1^). Against Gram-positive bacteria *S. pneumoniae*, compound **P_23_** (MIC 62.5 µg ml^−1^) was found to have outstanding activity; compounds **P_4_, P_6_, P_12_, P_17_, P_23_** (MIC 100 µg ml^−1^) showed equipotent activity when compared with ampicillin (MIC 100 µg ml^−1^), while against *B. subtilis*, compound **P_11_** (MIC 62.5 µg ml^−1^) displayed excellent activity; compounds **P_3_, P_5_, P_7_, P_12_, P_22_, P_23_, P_24_** (MIC 100 µg ml^−1^) showed significant activity; compounds **P_4_, P_9_, P_19_, P_20_** (MIC 125 µg ml^−1^) exhibited moderate activity; while compounds **P_1_, P_13_, P_15_, P_17_, P_18_, P_21_** (MIC 200 µg ml^−1^) showed better activity; while compounds **P_6_, P_8_, P_10_, P_14_** (MIC 250 µg ml^−1^) were found equally potent when compared with ampicillin (MIC 250 µg ml^−1^).

#### 3.2.3. Fungi

Assessment of anti-fungal screening data table 3 revealed that, against *C. albicans*, compounds **P_24_, P_16_** (MIC 100 µg ml^−1^) were found to have outstanding activity; compounds **P_2_** (MIC 200 µg ml^−1^) and **P_9_, P_10_, P_15_, P_19_** (MIC 250 µg ml^−1^) displayed significant activity; while compounds **P_1_, P_6,_ P_17_, P_18_, P_23_** showed equal potency when compared with griseofulvin (MIC 500 µg ml^−1^). Also on comparison with nystatin (MIC 100 µg ml^−1^) compounds **P_24_, P_16_** (MIC 100 µg ml^−1^) were equivalently potent. Against *T. rubrum* compounds **P_3_, P_6_, P_10_, P_15_, P_19_** and **P_24_** (MIC 500 µg ml^−1^) were found to be equally potent to nystatin as well as griseofulvin (MIC 500 µg ml^−1^).

### 3.3. Structural activity relationship study

The structural activity relationship (SAR) analysis demonstrated that a change in the peripheral substituent might also affect the anti-microbial activity of title compounds. The investigation revealed that the compounds with 4-chloro and bromo phenyl rings at the 2-position of the indole nucleus and also the lipophilicity of the –COO-methyl and ethyl groups at the R1 and the R2 position, respectively, play an important role to stimulate the potency of the compounds and gave excellent results towards Gram-negative bacteria *E. coli, S. typhi* and *V. cholera* and fungal pathogens as well. Whereas, towards *S. pnemoniae*, compounds without or with phenyl ring substitutions of the indole nucleus and also the lipophilicity of the ethyl groups at the R1 and the R2 position were found to be highly competent and showed equal activity to that of ampicillin. Against *B. subtilis* and *C. tetani*, all compounds carrying different substitutions were found to be effective. Anti-fungal evaluation showed that 4-fluoro and chloro phenyl ring-containing compounds are the most potent. Thus, examining and analysing the activity data, it is noteworthy that the anti-microbial activity of the target compounds depends not only on the bicyclic heteroaromatic pharmacophore appended through the aryl ring but also upon their positional changes, spatial relationship and also on the nature of the peripheral substituents.

## 4. Conclusion

In conclusion, we have demonstrated a convenient method for the synthesis of dihydropyrano [3,2-*c*] quinoline derivatives of indoles by applying a three-component reaction in the presence of a commercially available, non-toxic, inexpensive, biodegradable and easy-to-handle tetra-*n*-butylammonium fluoride (TBAF) in aqueous ethanol. The plainness of the reaction, easy isolation procedure, excellent yields of the products and short reaction time make it an efficient route for synthesizing pyranoquinoline heterocycles. So this novel organocatalyst may find application in one-pot three-component reaction for the synthesis of pyranoquinoline derivatives also at a large scale. It can be concluded from anti-microbial screening that most of the synthesized 4*H*-pyran derivatives were found to be highly active against a panel of human pathogens, compared to the standard drugs. It is worth affirming that minor changes in molecular configuration due to their positional changes, spatial relationship and also the nature of the peripheral substituents of these compounds profoundly influence their activity.

## Supplementary Material

Supplementary Material

## Data accessibility

We have included all the experimental data including spectral characterizations in the electronic supplementary material.
