# Supplementary material for: An efficient synthesis of 4H-pyrano quinolinone derivatives catalysed by a versatile organocatalyst tetra-n-butylammonium fluoride and their pharmacological screening
Source: R Soc Open Sci. 2017 Nov 29;4(11):170764. doi: 10.1098/rsos.170764 (PMC5717643; doi:10.1098/rsos.170764)
Supplement: Supplementary Material [file rsos170764supp1.doc]

**Supplementary Information**

**An efficient synthesis of 4H-pyrano quinolinone derivatives catalysed by a versatile organocatalyst TBAF and their pharmacological screening**

Pratibha Prasad, Pratik. G. Shobhashana, and Manish P. Patel*

Department of Chemistry, Sardar Patel University, Vallabh Vidyanagar-388120, Gujarat, India

| **S. No.** | **Table of Contents** | **Page** |
| --- | --- | --- |
|  | Spectral characterization of compounds | 2-7 |
|  | The ^1^H NMR spectrum of compound **P_1_ - P_24_** | 8-31 |
|  | The ^13^C NMR spectrum of compound **P_1_ -P_23_** | 32-54 |
|  | The Mass spectrum of compound **P_5_** | 55 |
|  | The Mass spectrum of compound **P_14_** | 56 |
|  | The Mass spectrum of compound **P_17_** | 57 |

**4-(1-allyl-2-phenyl-1*H*-indol-3-yl)-2-amino-6-methyl-5-oxo-5,6-dihydro-4*H*-pyrano[3,2-*c*]quinoline-3-carbonitrile (P_1_):** White solid; m.p 256^o^C; IR (KBr, ν, cm^-1^): 3,455 and 3,390 (asym. and sym. str. of –NH_2_), 2,190 (C≡N str.), 1,676 (C=O str.), ^1^H NMR (400 MHz, DMSO-*d_6_*): *δ* 3.49 (s,3H,N-CH_3_), 4.55-4.70 (m,4H,N-CH_2_CH=CH_2_), 5.74-5.82 (m,1H,NCH_2_CH), 5.03 (s,1H,CH), 6.91-8.04 (m,15H,Ar-H+NH_2_) ppm; ^13^CNMR (100 MHz DMSO-*d_6_*): *δ* 29.22, 26.83, 45.43, 57.56, 107.95, 110.68, 112.50, 113.94, 114.84, 115.90, 117.72,119.29, 119.86, 121.10, 122.09, 122.15, 125.48, 127.13, 127.91, 128.34, 130.96, 131.46, 134.12,136.07, 138.27, 138.51, 149.89, 158.16, 159.92(C=O); Anal. Calc. for: C_31_H_24_N_4_O_2_ (484.19)**:** C, 76.84; H, 4.99; N, 11.56; Found: C, 76.73; H, 4.91; N, 11.59 %

**Methyl-4-(1-allyl-2-phenyl-1*H*-indol-3-yl)-2-amino-6-methyl-5-oxo-5,6-dihydro-4*H*-pyrano[3,2-*c*]quinoline-3-carboxylate (P_2_):** White solid; m.p 270^o^C; IR (KBr, ν, cm^-1^): 3,426 and 3,385 (asym. and sym. str. of –NH_2_), 1,648 and 1,697 (C=O str.),^1^H NMR (400 MHz, DMSO-*d_6_*): *δ* 3.62 (s, 3H,N-CH_3_), 3.26 (s,3H,COOCH_3_), 4.52-4.68 (m,4H,N-CH_2_CH=CH_2_), 5.76-5.92 (m,1H,NCH_2_CH), 5.06 (s,1H,CH), 6.98-7.97 (m,15H,Ar-H+NH_2_) ppm; ^13^CNMR (100 MHz DMSO-*d_6_*): *δ* 26.35, 29.26, 45.62, 58.14, 77.14, 109.01, 110.31, 110.70, 112.81, 114.69, 115.88,116.19, 118.65, 118.94, 120.78, 121.92, 122.20, 123.98, 126.44, 127.75, 131.08, 131.89, 134.31,135.79, 137.91, 138.28, 149.97, 158.64, 168.27, 160.46(C=O);Anal. Calc. for: C_32_H_27_N_3_O_4_ (517.20)**:** C, 74.26; H, 5.26; N, 8.12; Found: C, 74.18; H, 5.28; N, 8.09 %

**Ethyl-4-(1-allyl-2-phenyl-1*H*-indol-3-yl)-2-amino-6-methyl-5-oxo-5,6-dihydro-4*H*-pyrano[3,2-*c*]quinoline-3-carboxylate (P_3_):** White solid; m.p 263^o^C; IR (KBr, ν, cm^-1^): 3,431 and 3,392 (asym. and sym. str. of –NH_2_), 1,651and 1,710 (C=O str.),^1^H NMR (400 MHz, DMSO- *d_6_*): *δ* 0.85 (s,3H,COOCH_2_CH_3_), 3.53-3.90 (m,5H,COOCH_2_,N-CH_3_), 4.48-4.71 (m,4H,N-CH_2_CH=CH_2_), 5.64-5.79 (m,1H,NCH_2_CH), 5.24 (s,1H,CH), 6.91 -8.02 (m,15H,Ar-H+NH_2_) ppm; ^13^CNMR (100 MHz DMSO-*d_6_*): *δ* 14.34, 26.35, 29.26, 45.62, 58.14, 77.14, 110.31, 110.70, 112.81, 114.70, 115.88, 116.19,118.65, 118.94, 120.78, 121.93, 122.20, 126.02, 126.94, 127.75, 130.83, 131.09, 131.88, 134.32, 135.78, 137.91, 138.28, 149.98, 158.64, 168.27, 160.46(C=O); Anal. Calc. for: C_33_H_29_N_3_O_4_ (531.22)**:** C, 74.56; H, 5.50; N, 7.90; Found: C, 74.59; H, 5.54; N, 7.95 %

**4-(1-allyl-2-phenyl-1*H*-indol-3-yl)-2-amino-6-ethyl-5-oxo-5,6-dihydro-4*H*-pyrano[3,2-*c*]quinoline-3-carbonitrile (P_4_):** White solid; m.p 257^o^C; IR (KBr, ν, cm^-1^): 3,451 and 3,410 (asym. and sym. str. of –NH_2_), 2,175 (C≡N str.), 1,682 (C=O str.), ^1^H NMR (400 MHz, DMSO-*d_6_*): *δ* 1.16 (t,3H,N-CH_2_CH_3_), 4.10-4.12 (m,2H,N-CH_2_CH_3_), 4.49-4.68 (m,4H,N-CH_2_CH=CH_2_), 5.79-6.01 (m,1H,NCH_2_CH), 5.20 (s,1H,CH), 6.78-8.03 (m,15H,Ar-H+NH_2_) ppm; ^13^CNMR (100 MHz DMSO-*d_6_*): *δ* 12.67, 28.39, 36.66, 45.59, 57.65, 107.92, 110.64, 112.73, 114.13, 114.57, 115.80,117.77, 119.30, 119.90, 121.13, 121.92, 122.43, 125.60, 126.86, 127.98, 128.31, 130.98, 131.50, 134.16, 136.01, 137.41, 138.17, 149.92, 158.18, 159.50(C=O);Anal. Calc. for: C_32_H_26_N_4_O_2_ (498.21)**:** C, 77.09; H, 5.26; N, 11.24; Found: C, 77.25; H, 5.45; N, 11.45 %

**Methyl-4-(1-allyl-2-phenyl-1*H*-indol-3-yl)-2-amino-6-ethyl-5-oxo-5,6-dihydro-4*H*-pyrano[3,2-*c*]quinoline-3-carboxylate (P_5_):** White solid; m.p 265^o^C; IR (KBr, ν, cm^-1^): 3,315 and 3,285 (asym. and sym. str. of –NH_2_), 1,680 and 1,692 (C=O str.), ^1^H NMR (400 MHz, DMSO-*d_6_*): *δ* 1.14 (t,3H,N-CH_2_CH_3_), 3.15 (s,3H,COOCH_3_), 4.09-4.32 (m,2H,N-CH_2_CH_3_), 4.55-4.70 (m,4H,N-CH_2_CH=CH_2_), 5.74-5.81 (m,1H,NCH_2_CH), 5.18 (s,1H,CH), 6.89-8.02 (m,15H,Ar-H+NH_2_) ppm; ^13^CNMR (100 MHz DMSO-*d_6_*):*δ* 13.24, 26.71, 37.16, 46.02, 58.68, 77.79, 108.16,109.29, 110.74, 111.20, 113.56, 114.92, 116.20, 116.94, 119.40, 121.29, 122.23, 122.98,125.54, 128.10, 129.57, 131.62, 132.35, 134.84, 136.22, 137.69, 138.43, 150.47, 159.18, 168.78, 160.51(C=O); MS (m/z) Cald.: 531.22, found: 529.80 [M^+^], Anal. Calc. for: C_33_H_29_N_3_O_4_**:** C, 74.56; H, 5.50; N, 7.90; Found: C, 73.76; H, 5.47; N, 7.94 %

**Ethyl-4-(1-allyl-2-phenyl-1*H*-indol-3-yl)-2-amino-6-ethyl-5-oxo-5,6-dihydro-4*H*-pyrano[3,2-*c*]quinoline-3-carboxylate(P_6_):** White solid; m.p. 254^o^C; IR (KBr, ν, cm^-1^): 3,375 and 3,285 (asym. and sym. str. of –NH_2_), 1,684 and 1,725 (C=O str.), ^1^H NMR (400 MHz, DMSO-*d_6_*): *δ* 0.85 (t,3H,COOCH_2_CH_3_), 1.15 (t,3H,N-CH_2_CH_3_), 3.57-4.08 (m,2H,COOCH_2_), 4.07-4.34 (m,2H,N-CH_2_CH_3_), 4.64-4.73 (m,4H,N-CH_2_CH=CH_2_), 5.72 -5.82 (m,1H,NCH_2_CH), 5.19 (s,1H,CH), 6.79-8.04 (m,15H,Ar-H+NH_2_) ppm; ^13^CNMR (100 MHz DMSO-*d_6_*): *δ* 12.75, 14.34, 26.21, 36.67, 45.53, 58.15, 77.18, 108.02, 110.29, 110.67, 113.03, 114.45, 115.69, 116.33, 118.78, 118.93, 120.80, 121.77, 122.47, 125.31, 128.11, 129.77, 131.15, 131.84, 134.36,135.73, 137.18, 137.90, 149.99, 158.67, 168.26, 160.01(C=O); Anal. Calc. for: C_34_H_31_N_3_O_4_ (545.23)**:** C, 74.84; H, 5.73; N, 7.70; Found: C, 73.94; H, 5.68; N, 7.780 %

**4-(1-allyl-2-(4-bromophenyl)-1*H*-indol-3-yl)-2-amino-6-methyl-5-oxo-5,6-dihydro-4*H*-pyrano[3,2-*c*]quinoline-3-carbonitrile (P_7_):** White solid; m.p. 278^o^ C; IR (KBr, ν, cm^-1^): 3,410 and 3,351 (asym. and sym. str. of –NH_2_), 2,210 (C≡N str.), 1,710 (C=O str.), ^1^H NMR (400 MHz, DMSO-*d_6_*): *δ* 3.58 (s, 3H,N-CH_3_), 4.53-4.69 (m,4H,N-CH_2_CH=CH_2_),5.69-5.94 (m,1H,NCH_2_CH), 5.20 (s,1H,CH), 6.98-8.03 (m,14H,Ar-H+NH_2_) ppm; ^13^CNMR (100 MHz DMSO-*d_6_*): *δ* 29.03, 26.52, 45.81, 57.50, 108.03, 108.35, 110.27, 110.66, 113.02, 114.47, 115.74, 115.35, 118.77, 118.95, 120.77, 121.83, 122.43, 125.39, 128.23, 129.62, 131.14, 131.89, 134.34, 135.84, 137.11, 137.88, 149.89, 159.72, 161.01(C=O);Anal. Calc. for: C_31_H_23_BrN_4_O_2_ (562.10)**:** C, 66.08; H, 4.11; N, 9.94; Found: C, 65.98; H, 4.16; N, 9.89 %

**Methyl-4-(1-allyl-2-(4-bromophenyl)-1*H*-indol-3-yl)-2-amino-6-methyl-5-oxo-5,6-dihydro-4*H*-pyrano[3,2-*c*]quinoline-3-carboxylate (P_8_):** White solid; m.p. 291^o^C; IR (KBr, ν, cm^-1^): 3,391 and 3,270 (asym. and sym. str. of –NH_2_), 1,671 and 1,720(C=O str.), ^1^H NMR (400 MHz, DMSO-*d_6_*): *δ* 3.54 (s, 3H,N-CH_3_), 3.15 (s,3H,COOCH_3_), 4.54-4.72 (m,4H,N-CH_2_CH=CH_2_),5.76-5.84 (m,1H,NCH_2_CH), 5.15 (s,1H,CH), 6.85-8.04 (m,14H,Ar-H+NH_2_) ppm; ^13^CNMR (100 MHz DMSO-*d_6_*): *δ* 29.29, 26.33, 45.55, 58.22, 77.06, 108.53, 110.32, 110.62, 112.79, 114.77, 115.86, 116.49, 118.70, 119.00, 120.91, 122.00, 122.14, 125.01, 127.54, 129.19, 131.15, 132.77, 134.29, 135.74, 136.79, 138.28, 149.92, 158.65, 168.21, 160.71(C=O); Anal. Calc. for: C_32_H_26_BrN_3_O_4_ (595.11)**:** C, 64.44; H, 4.39; N, 7.04; Found: C, 63.94; H, 4.43; N, 7.14 %

**Ethyl-4-(1-allyl-2-(4-bromophenyl)-1*H*-indol-3-yl)-2-amino-6-methyl-5-oxo-5,6-dihydro-4*H*-pyrano[3,2-*c*]quinoline-3-carboxylate (P_9_):** White solid; m.p. 286^o^C; IR (KBr, ν, cm^-1^): 3,394 and 3,287 (asym. and sym. str. of –NH_2_), 1,691 and 1,730 (C=O str.), ^1^H NMR (400 MHz, DMSO-*d_6_*): *δ* 0.82 (t,3H,COOCH_2_CH_3_), 3.56 (s,3H,N-CH_3_), 3.94 (q,2H,COOCH_2_CH_3_), 4.47 (d,2H,N-CH_2_), 4.66 (d,1H,N-CH_2_CH=CH_2_ trans), 5.00 (d,1H,N-CH_2_CH=CH_2_cis), 5.19 (s,1H,CH), 5.76-5.84 (m,1H,NCH_2_CH), 6.92-8.35 (m,14H,Ar-H+NH_2_) ppm; ^13^CNMR (100 MHz DMSO-*d_6_*): *δ* 14.87, 26.83, 29.78, 46.02, 58.72, 77.63, 106.30, 106.88, 110.74, 111.15, 112.23, 113.28, 115.28, 116.37, 119.27, 119.50, 121.36, 122.44, 122.66, 128.64, 129.62, 130.68, 131.62, 134.77, 136.24, 137.29, 137.51, 139.00, 159.03, 168.70, 161.02(C=O); Anal. Calc. for: C_33_H_28_BrN_3_O_4_ (609.13)**:** C, 64.92; H, 4.62; N, 6.88; Found: C, 65.12; H, 5.32; N, 7.08%

**4-(1-allyl-2-(4-bromophenyl)-1*H*-indol-3-yl)-2-amino-6-ethyl-5-oxo-5,6-dihydro-4*H*-pyrano[3,2-*c*]quinoline-3-carbonitrile (P_10_):** White solid; m.p. 241^o^C; IR (KBr, ν, cm^-1^): 3,396 and 3,310 (asym. and sym. str. of –NH_2_), 2,195 (C≡N str.), 1,735 (C=O str.),^1^H NMR (400 MHz, DMSO-*d_6_*): *δ* 1.14 (t,3H,N-CH_2_CH_3_), 4.10-4.30 (m,2H,N-CH_2_CH_3_), 4.48-4.97 (m,4H,N-CH_2_CH=CH_2_), 5.20 (s,1H,CH), 5.79-5.85 (m,1H,NCH_2_CH), 6.78-8.03 (m,14H,Ar-H+NH_2_) ppm; ^13^CNMR (100 MHz DMSO-*d_6_*): *δ* 12.66, 28.40, 36.66, 45.39, 57.66, 107.93, 110.64, 112.73, 114.13, 114.55, 115.80, 117.78, 119.30, 119.91, 121.13, 121.91, 122.42, 124.15, 127.06, 127.92, 128.31, 130.98, 131.49, 134.15, 136.01, 137.41, 138.18, 149.93, 158.18, 159.50(C=O); Anal. Calc. for: C_32_H_25_BrN_4_O_2_ (576.12)**:** C, 66.56; H, 4.36; N, 9.70; Found: C, 66.61; H, 4.39; N, 9.85 %

**Methyl-4-(1-allyl-2-(4-bromophenyl)-1*H*-indol-3-yl)-2-amino-6-ethyl-5-oxo-5,6-dihydro-4*H*-pyrano[3,2-*c*]quinoline-3-carboxylate (P_11_):** White solid; m.p. 253^o^C; IR (KBr, ν, cm^-1^): 3,387 and 3,276 (asym. and sym. str. of –NH_2_), 1,692 and1,731(C=O str.), ^1^H NMR (400 MHz, DMSO-*d_6_*): *δ* 1.13 (t,3H,N-CH_2_CH_3_), 4.08-430. (m,2H,N-CH_2_CH_3_), 3.16 (s,3H,COOCH_3_), 4.52-4.69 (m,4H,N-CH_2_CH=CH_2_), 5.74-5.81 (m,1H,NCH_2_=CH), 5.19 (s,1H,CH), 6.87-8.28 (m,14H,Ar-H+NH_2_) ppm; ^13^CNMR (100 MHz DMSO-*d_6_*): *δ* 12.74, 26.19, 36.68, 45.45, 58.21, 77.10, 106.00, 107.95, 110.28, 110.59, 113.00, 114.50, 115.63, 116.64, 118.81, 118.98, 120.92, 121.82, 122.39, 124.02, 127.80, 128.14, 131.20, 134.32, 135.69, 136.76, 137.15, 149.92, 159.99, 168.20, 160.69(C=O); Anal. Calc. for: C_33_H_28_BrN_3_O_4_ (609.13)**:** C, 64.92; H, 4.62; N, 6.88; Found: C, 65.01; H, 4.71; N, 6.93 %

**Ethyl-4-(1-allyl-2-(4-bromophenyl)-1*H*-indol-3-yl)-2-amino-6-ethyl-5-oxo-5,6-dihydro-4*H*-pyrano[3,2-*c*]quinoline-3-carboxylate (P_12_):** White solid; m.p. 263^o^C; IR (KBr, ν, cm^-1^): 3,400 and 3,385 (asym. and sym. str. of –NH_2_), 1,685 and 1,720 (C=O str.), ^1^H NMR (400 MHz, DMSO-*d_6_*): *δ* 0.82 (t,3H,COOCH_2_CH_3_), 1.14 (t,3H,N-CH_2_CH_3_), 3.94 (q,2H,COOCH_2_CH_3_), 4.10-4.28 (m,2H,N-CH_2_CH_3_), 4.50-4.68 (m,4H,N-CH_2_CH=CH_2_), 5.20 (s,1H,CH), 5.75-5.83 (m,1H,NCH_2_=CH), 6.90-8.22 (m,14H,Ar-H+NH_2_) ppm; ^13^CNMR (100 MHz DMSO-*d_6_*): *δ* 12.74, 14.35, 26.19, 36.69, 45.46, 58.21, 77.10, 108.86, 110.28, 110.59, 113.00, 114.50, 115.63, 116.64, 118.82, 118.98, 120.92, 121.82, 122.39, 124.98, 126.40, 128.14, 131.20, 134.32, 135.70, 136.76, 137.16, 138.57, 149.92, 158.67, 168.20, 160.70(C=O); Anal. Calc. for: C_34_H_30_BrN_3_O_4_ (623.14)**:** C, 65.39; H, 4.84; N, 6.73; Found: C, 65.41; H, 4.89; N, 6.85 %

**4-(1-allyl-2-(4-fluorophenyl)-1*H*-indol-3-yl)-2-amino-6-methyl-5-oxo-5,6-dihydro-4*H*-pyrano[3,2-*c*]quinoline-3-carbonitrile (P_13_):** White solid; m.p. 278^o^C; IR (KBr, ν, cm^-1^): 3,387 and 3,276 (asym. and sym. str. of –NH_2_), 2,214 (C≡N str.), 1,727 (C=O str.), ^1^H NMR (400 MHz, DMSO-*d_6_*): *δ* 3.50 (s, 3H,N-CH_3_), 4.56-4.69 (m,4H,N-CH_2_CH=CH_2_), 5.05 (s,1H,CH), 5.79-5.86 (m,1H,NCH_2_CH), 6.90-8.06 (m,14H,Ar-H+NH_2_) ppm; ^13^CNMR (100 MHz DMSO-*d_6_*): *δ*26.82, 29.86, 45.99, 58.14, 103.69, 108.18, 109.25, 111.00, 113.09, 114.97, 115.48, 116.56, 118.37, 119.96, 120.32, 121.84, 122.71, 125.89, 129.87, 130.98, 131.60, 131.97, 133.56, 134.71, 136.74, 137.46, 158.58, 162.91, 160.46 (C=O); Anal. Calc. for: C_31_H_23_FN_4_O_2_ (502.18)**:** C, 74.09; H, 4.61; N, 11.15; Found: C, 74.14; H, 4.66; N, 11.06 %

**Methyl-4-(1-allyl-2-(4-fluorophenyl)-1*H*-indol-3-yl)-2-amino-6-methyl-5-oxo-5,6-dihydro-4*H*-pyrano[3,2-*c*]quinoline-3-carboxylate (P_14_):** White solid; m.p. 285^o^C; IR (KBr, ν, cm^-1^): 3,379 and 3,295 (asym. and sym. str. of –NH_2_), 1,687 and1,742(C=O str.), ^1^H NMR (400 MHz, DMSO-*d_6_*): *δ* 3.85 (s,3H,COOCH_3_), 3.54 (s, 3H,N-CH_3_), 4.44-5.01 (m,4H,N-CH_2_CH=CH_2_), 5.17 (s,1H,CH), 5.78-6.10 (m,1H,NCH_2_CH), 6.91-8.13 (m,14H,Ar-H+NH_2_) ppm; ^13^CNMR (100 MHz DMSO-*d_6_*): *δ* 26.80, 29.77, 46.02, 58.74,77.86, 110.63, 111.33, 113.43, 113.64, 115.14, 116.78, 119.26, 121.08, 122.31, 122.72, 124.40, 127.13, 131.46, 132.40, 134.85, 136.04, 138.41, 138.83, 150.40, 159.10, 159.49, 163.61, 168.85, 160.95 (C=O); MS (m/z) Cald.: 535.19 , found: 535.28 [M^+^], Anal. Calc. for: C_31_H_26_FN_3_O_4_**:** C, 71.76; H, 4.89; N, 7.85; Found: C, 71.83; H, 5.02; N, 7.54 %

**Ethyl-4-(1-allyl-2-(4-fluorophenyl)-1*H*-indol-3-yl)-2-amino-6-methyl-5-oxo-5,6-dihydro-4*H*-pyrano[3,2-*c*]quinoline-3-carboxylate (P_15_):** White solid; m.p. 289^o^C; IR (KBr, ν, cm^-1^): 3,317 and 3,271 (asym. and sym. str. of –NH_2_), 1,714 and 1,745 (C=O str.),^1^H NMR (400 MHz, DMSO-*d_6_*): *δ* 0.85 (t,3H,COOCH_2_CH_3_), 3.52 (s, 3H,N-CH_3_), 3.52-4.07 (m,2H,COOCH_2_CH_3_), 4.56-4.67 (m,4H,N-CH_2_CH=CH_2_), 5.08 (s,1H,CH), 5.76 -5.81 (m,1H,NCH_2_CH), 6.87-8.02 (m,14H,Ar-H+NH_2_) ppm; ^13^CNMR (100 MHz DMSO-*d_6_*): *δ* 14.34, 26.39, 29.30, 45.61, 58.20, 76.97, 108.38, 110.46, 112.75, 114.78, 115.86, 116.59, 118.61, 119.10, 121.05, 121.57, 122.01, 122.18, 124.52, 127.86, 130.69, 131.18, 134.25, 135.97, 136.50, 138.27, 149.97, 158.64, 162.18, 168.15, 160.43 (C=O);Anal. Calc. for: C_33_H_28_FN_3_O_4_ (549.21)**:** C, 72.12; H, 5.14; N, 7.65; Found: C, 71.98; H, 5.24; N, 7.73 %

**4-(1-allyl-2-(4-fluorophenyl)-1*H*-indol-3-yl)-2-amino-6-ethyl-5-oxo-5,6-dihydro-4*H*-pyrano[3,2-*c*]quinoline-3-carbonitrile (P_16_):** White solid; m.p. 321^o^C; IR (KBr, ν, cm^-1^): 3,373 and 3,280 (asym. and sym. str. of –NH_2_), 2,210 (C≡N str.), 1,731 (C=O str.), ^1^H NMR (400 MHz, DMSO-*d*_6_): *δ* 1.16 (t,3H,N-CH_2_CH_3_), 4.08-4.29 (m,2H,N-CH_2_CH_3_), 4.51-4.75 (m,4H,N-CH_2_CH=CH_2_), 5.18 (s,1H,CH), 5.73-5.82 (m,1H,NCH_2_CH), 6.87-8.12 (m,14H,Ar-H+NH_2_) ppm; ^13^CNMR (100 MHz DMSO-*d_6_*): *δ* 12.67, 28.40, 36.69, 45.38, 57.52, 107.74, 110.70, 112.69, 114.61, 115.73, 117.85, 119.44, 119.88, 121.41, 122.03, 122.20, 122.40, 125.50, 130.25, 130.98, 131.57, 132.95, 134.15, 136.14, 136.70, 137.38, 149.95, 158.09, 162.82, 159.51 (C=O); Anal. Calc. for: C_32_H_25_FN_4_O_2_ (516.20)**:** C, 74.40; H, 4.88; N, 10.85; Found: C, 74.32; H, 5.08; N, 11.23%

**Methyl-4-(1-allyl-2-(4-fluorophenyl)-1*H*-indol-3-yl)-2-amino-6-ethyl-5-oxo-5,6-dihydro-4*H*-pyrano[3,2-*c*]quinoline-3-carboxylate (P_17_):** White solid; m.p. 282^o^C; IR (KBr, ν, cm^-1^): 3,394 and 3,279 (asym. and sym. str. of –NH_2_), 1,691 and 1,713 (C=O str.), ^1^H NMR (400 MHz, DMSO-*d_6_*): *δ* 1.18 (t,3H,N-CH_2_CH_3_), 4.10-4.30 (m,2H,N-CH_2_CH_3_), 3.21 (s, 3H,COOCH_3_), 4.59-4.73 (m,4H,N-CH_2_CH=CH_2_), 5.14 (s,1H,CH), 5.75-5.87 (m,1H,NCH_2_CH), 6.86-8.10 (m,14H,Ar-H+NH_2_) ppm; ^13^CNMR (100 MHz DMSO-*d_6_*): *δ* 12.74, 26.25, 36.69, 45.51, 58.22, 77.06, 110.45, 112.97, 114.49, 115.64, 116.78, 118.77, 119.07, 121.08, 121.62, 121.85, 122.44, 126.31, 130.75, 131.12, 131.22, 132.76, 134.28, 135.92, 136.48, 137.15, 149.97, 158.67, 163.37, 168.16, 159.96 (C=O); MS (m/z) Cald.: 549.21, found: 550.30 [M^+^], Anal. Calc. for: C_33_H_28_FN_3_O_4_**:** C, 72.12; H, 5.14; N, 7.65; Found: C, 72.10; H, 5.04; N, 7.83 %

**Ethyl-4-(1-allyl-2-(4-fluorophenyl)-1*H*-indol-3-yl)-2-amino-6-ethyl-5-oxo-5,6-dihydro-4*H*-pyrano[3,2-*c*]quinoline-3-carboxylate (P_18_):** White solid; m.p. 276^o^C; IR (KBr, ν, cm^-1^): 3,356 and 3,289 (asym. and sym. str. of –NH_2_), 1,740 and 1,710 (C=O str.), ^1^H NMR (400 MHz, DMSO-*d_6_*): *δ* 0.84 (t,3H,COOCH_2_CH_3_), 1.17 (t,3H,N-CH_2_CH_3_), 3.56-4.03 (q,2H,COOCH_2_CH_3_), 4.08-4.31 (m,2H,N-CH_2_CH_3_), 4.53-4.65 (m,4H,N-CH_2_CH=CH_2_), 5.13 (s,1H,CH), 5.75-5.82 (m,1H,NCH_2_CH), 6.85-8.12 (m,14H,Ar-H+NH_2_) ppm; ^13^CNMR (100 MHz DMSO-*d_6_*): *δ* 12.74, 14.34, 26.25, 36.70, 45.51, 58.22, 77.05, 110.45, 112.97, 114.49, 115.65, 116.78, 118.78, 119.07, 121.07, 121.62, 121.85, 122.44, 126.31, 130.73, 131.12, 131.22, 132.76, 134.28, 135.91, 136.48, 137.15, 149.97, 158.97, 162.88, 168.16, 159.96 (C=O);Anal. Calc. for: C_34_H_30_FN_3_O_4_ (563.22)**:** C, 72.45; H, 5.37; N, 7.46; Found: C, 72.39; H, 5.41; N, 7.53 %

**4-(1-allyl-2-(4-chlorophenyl)-1*H*-indol-3-yl)-2-amino-6-methyl-5-oxo-5,6-dihydro-4*H*-pyrano[3,2-*c*]quinoline-3-carbonitrile (P_19_):** White solid; m.p. 275^o^C; IR (KBr, ν, cm^-1^): 3,342 and 3,295 (asym. and sym. str. of –NH_2_), 2,186 (C≡N str.), 1,728 (C=O str.), ^1^H NMR (400 MHz, DMSO-*d_6_*): *δ* 3.52 (s,3H,N-CH_3_), 4.57-4.71 (m,4H,N-CH_2_CH=CH_2_), 5.14 (s,1H,CH), 5.79-5.85 (m,1H,NCH_2_CH), 6.91-8.04 (m,14H,Ar-H+NH_2_) ppm; ^13^CNMR (100 MHz DMSO-*d_6_*): *δ* 26.54, 29.13, 45.85, 57.61, 108.05, 108.47, 110.35, 110.65, 113.13, 115.35, 115.83, 116.31, 118.79, 118.98, 120.77, 121.64, 122.42, 125.36, 128.25, 129.62, 131.11, 131.77, 134.34, 135.84, 137.14, 137.88, 149.89, 159.83, 161.07 (C=O); Anal. Calc. for: C_31_H_23_ClN_4_O_2_ (518.15)**:** C, 71.74; H, 4.47; N, 10.80; Found: C, 71.91; H, 4.71; N, 10.78%

**Methyl-4-(1-allyl-2-(4-chlorophenyl)-1*H*-indol-3-yl)-2-amino-6-methyl-5-oxo-5,6-dihydro-4*H*-pyrano[3,2-*c*]quinoline-3-carboxylate (P_20_):** White solid; m.p. 268^o^C; IR (KBr, ν, cm^-1^): 3,386 and 3,219 (asym. and sym. str. of –NH_2_), 1,720 and 1,689 (C=O str.), ^1^H NMR (400 MHz, DMSO-*d_6_*): *δ* 3.17 (s,3H,COOCH_3_), 3.52 (s,3H,N-CH_3_), 4.55-4.73 (m,4H,N-CH_2_CH=CH_2_), 5.15 (s,1H,CH), 5.76-5.87 (m,1H,NCH_2_CH), 6.87-8.06 (m,14H,Ar-H+NH_2_) ppm; ^13^CNMR (100 MHz DMSO-*d_6_*): *δ* 26.27, 29.31, 45.59, 58.17, 77.05, 108.49, 110.35, 110.77, 112.81, 114.62, 115.91, 116.65, 118.62, 119.15, 120.86, 122.03, 122.19, 125.16, 127.63, 129.22, 131.11, 132.81, 134.43, 135.82, 136.67, 138.32, 149.87, 158.89, 168.24, 160.67 (C=O); Anal. Calc. for: C_32_H_26_ClN_3_O_4_ (551.16)**:** C, 69.63; H, 4.75; N, 7.61; Found: C, 69.43; H, 4.68; N, 7.85 %

**Ethyl-4-(1-allyl-2-(4-chlorophenyl)-1*H*-indol-3-yl)-2-amino-6-methyl-5-oxo-5,6-dihydro-4*H*-pyrano[3,2-*c*]quinoline-3-carboxylate (P_21_):** White solid; m.p. 312^o^C; IR (KBr, ν, cm^-1^): 3,391 and 3,275 (asym. and sym. str. of –NH_2_), 1,760 and1,684 (C=O str.), ^1^H NMR (400 MHz, DMSO-*d_6_*): *δ* 0.87 (t,3H,COOCH_2_CH_3_), 3.54 (s,3H,N-CH_3_), 3.51-4.09 (m,2H,COOCH_2_CH_3_), 4.54-4.69 (m,4H,N-CH_2_CH=CH_2_), 5.17 (s,1H,CH), 5.74-5.86 (m,1H,NCH_2_CH), 6.89-8.06 (m,14H,Ar-H+NH_2_) ppm; ^13^CNMR (100 MHz DMSO-*d_6_*): *δ* 14.46, 26.25, 30.73, 45.51, 58.26, 77.13, 108.92, 110.32, 110.83, 113.08, 114.45, 115.59, 116.56, 118.78, 118.98, 120.88, 121.87, 122.49, 124.98, 126.39, 128.24, 131.18, 134.35, 135.70, 136.82, 137.16, 138.65, 149.90, 158.79, 168.26, 160.76 (C=O); Anal. Calc. for: C_33_H_28_ClN_3_O_4_ (565.18)**:** C, 70.02; H, 4.99; N, 7.42; Found: C, 69.98; H, 4.87; N, 7.39 %

**4-(1-allyl-2-(4-chlorophenyl)-1H-indol-3-yl)-2-amino-6-ethyl-5-oxo-5,6-dihydro-4H-pyrano[3,2-c]quinoline-3-carbonitrile (P_22_):** White solid; m.p. 314^o^C; IR (KBr, ν, cm^-1^): 3,392 and 3,286 (asym. and sym. str. of –NH_2_), 2,192 (C≡N str.), 1,731 (C=O str.), ^1^H NMR (400 MHz, DMSO-*d_6_*): *δ* 1.10 (t,3H,N-CH_2_CH_3_), 4.04-4.24 (m,2H,N-CH_2_CH_3_), 4.55-4.70 (m,4H,N-CH_2_CH=CH_2_), 5.16 (s,1H,CH), 5.79-5.87 (m,1H,NCH_2_CH), 6.91-8.06 (m,14H,Ar-H+NH_2_) ppm; ^13^CNMR (100 MHz DMSO-*d_6_*): *δ* 13.16, 26.84, 37.19, 45.89, 58.16, 108.29, 111.17, 113.22, 115.08, 115.24, 116.27, 118.39, 119.92, 120.33, 121.89, 122.48, 122.90, 126.06, 128.56, 130.39, 132.04, 133.12, 133.92, 134.63, 136.63, 136.64, 137.18, 137.91, 158.61, 160.03 (C=O);Anal. Calc. for: C_32_H_25_ClN_4_O_2_ (532.17)**:** C, 72.11; H, 4.73; N, 10.51; Found: C, 72.01; H, 4.97; N, 10.78%

**Ethyl-4-(1-allyl-2-(4-chlorophenyl)-1*H*-indol-3-yl)-2-amino-6-ethyl-5-oxo-5,6-dihydro-4*H*-pyrano[3,2-*c*]quinoline-3-carboxylate (P_23_):** White solid; m.p 283^o^C; IR (KBr, ν, cm^-1^): 3,372 and 3,236 (asym. and sym. str. of –NH_2_), 1,724 and 1,693 (C=O str.), ^1^H NMR (400 MHz, DMSO-*d_6_*): *δ* 085 (s,3H, COOCH_2_CH_3_), 1.13 (t,3H,N-CH_2_CH_3_), 3.53-4.06 (m,2H,COOCH_2_CH_3_), 4.09-4.29 (m,2H,N-CH_2_CH_3_), 4.48-5.00 (m,4H,N-CH_2_CH=CH_2_), 5.20 (s,1H,CH), 5.75-5.83 (m,1H,NCH_2_CH), 6.93-8.04 (m,14H,Ar-H+NH_2_) ppm; ^13^CNMR (100 MHz DMSO-*d_6_*): *δ* 13.24, 14.82, 26.74, 37.19, 46.00, 58.74, 77.66, 110.84, 111.01, 113.49, 114.97, 116.16, 117.41, 119.36, 119.54, 121.55, 122.31, 122.91, 126.90, 128.22, 131.25, 132.70, 132.91, 133.37, 134.77, 135.56, 136.39, 136.97, 137.67, 159.17, 168.68, 160.47 (C=O);Anal. Calc. for: C_34_H_30_ClN_3_O_4_ (579.19)**:** C, 70.40; H, 5.21; N, 7.24; Found: C, 70.18; H, 5.46; N, 7.57 %

**Methyl-4-(1-allyl-2-(4-chlorophenyl)-1*H*-indol-3-yl)-2-amino-6-ethyl-5-oxo-5,6-dihydro-4*H*-pyrano[3,2-*c*]quinoline-3-carboxylate (P_24_):** White solid; m.p 295^o^C; IR (KBr, ν, cm^-1^): 3,364 and 3,215 (asym. and sym. str. of –NH_2_), 1,754 and 1,693 (C=O str.),^1^H NMR (400 MHz, DMSO-*d_6_*): *δ* 1.16 (t,3H,N-CH_2_CH_3_), 3.16 (s,3H,COOCH_3_), 4.07-4.25 (m,2H,N-CH_2_CH_3_), 4.52-4.65 (m,4H,N-CH_2_CH=CH_2_), 5.15 (s,1H,CH), 5.73-5.84 (m,1H,NCH_2_CH), 6.89-8.05 (m,14H,Ar-H+NH_2_) ppm; ^13^CNMR (100 MHz DMSO-*d_6_*): *δ* 13.1526.83, 37.26, 46.12, 58.35, 77.61, 110.78, 111.04, 113.52, 114.94, 116.22, 117.52, 119.34, 119.61, 121.51, 122.43, 122.89, 126.84, 128.18, 131.27, 132.76, 132.97, 133.41, 134.68, 135.54, 136.41, 136.93, 137.63, 158.71, 168.62, 160.59(C=O); Anal. Calc. for: C_33_H_28_ClN_3_O_4_ (565.18)**:** C, 70.02; H, 4.99; N, 7.42; Found: C, 69.97; H, 5.24; N, 7.14 %

^1^H NMR spectra of compound **P_1_**

**
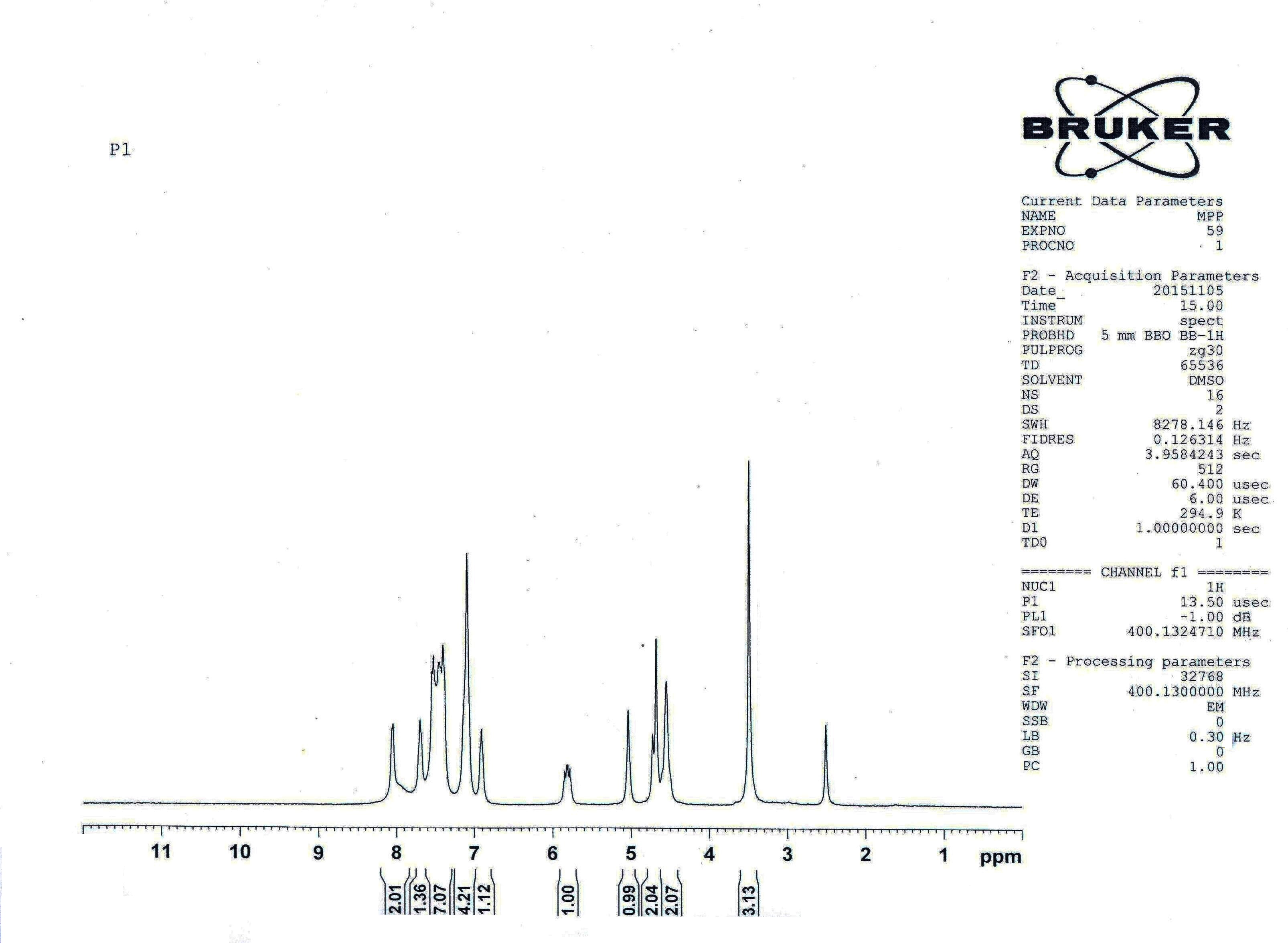
**

^1^H NMR spectra of compound **P_2_**


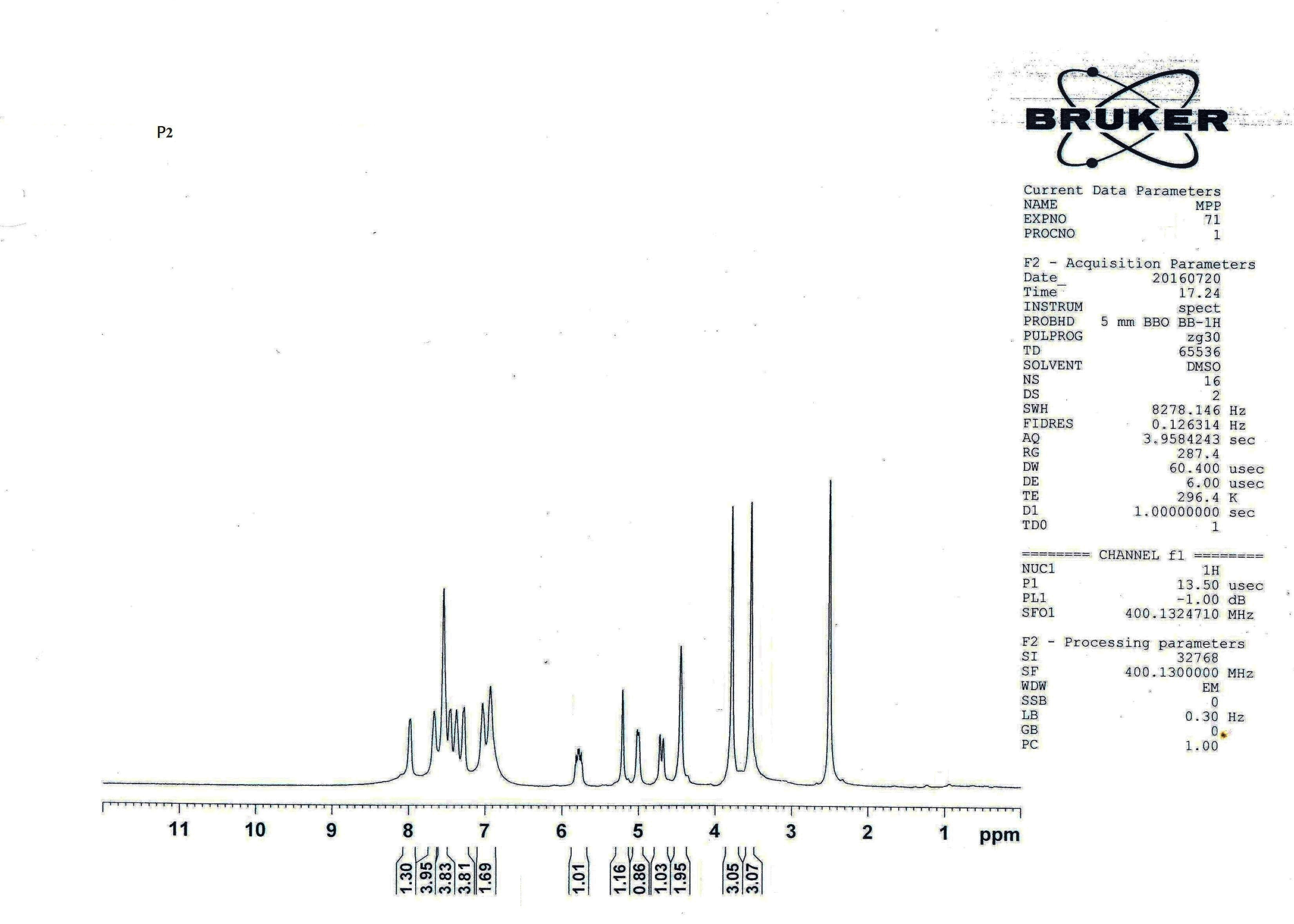


^1^H NMR spectra of compound **P_3_**


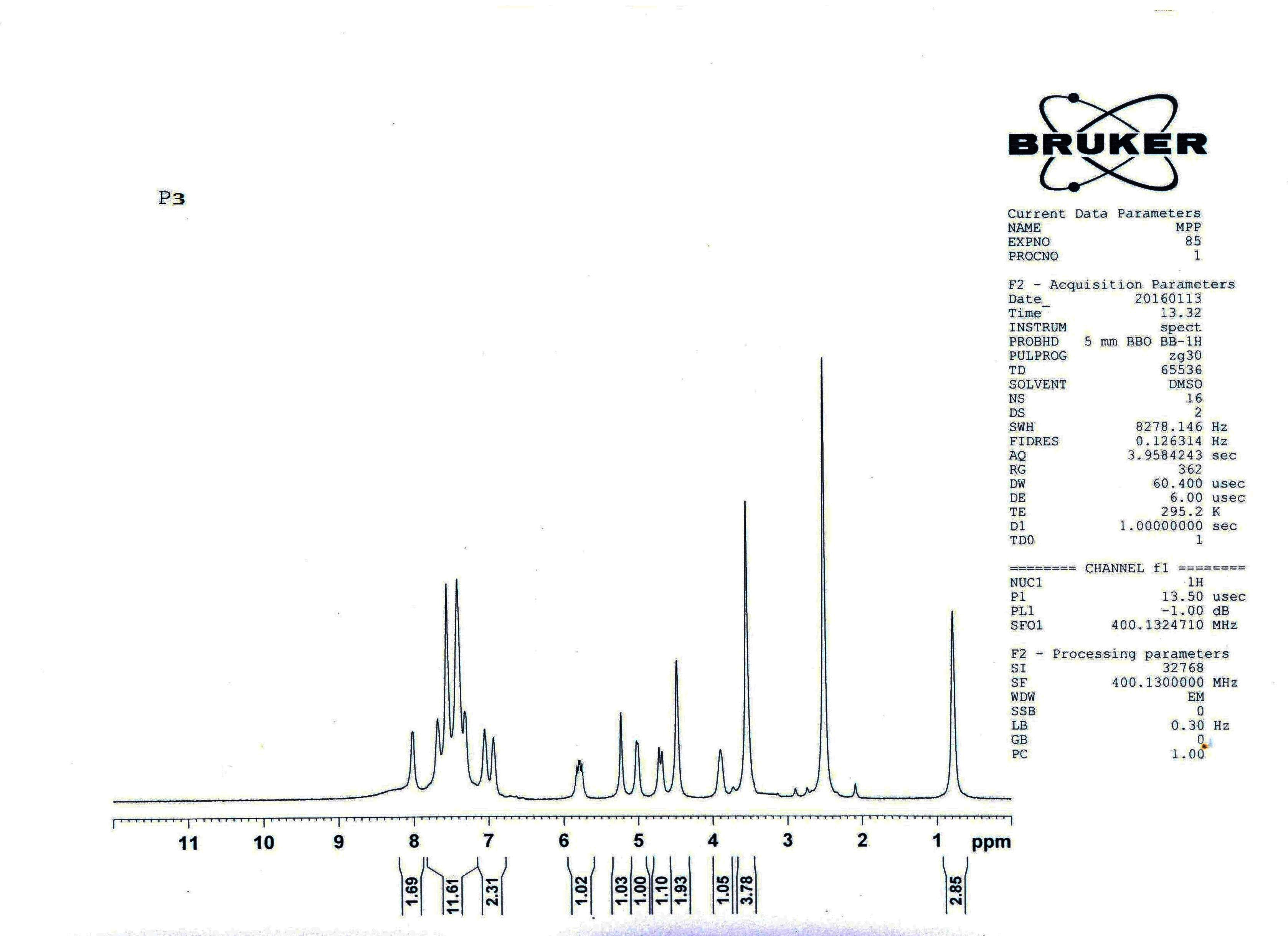


^1^H NMR spectra of compound **P_4_**

**
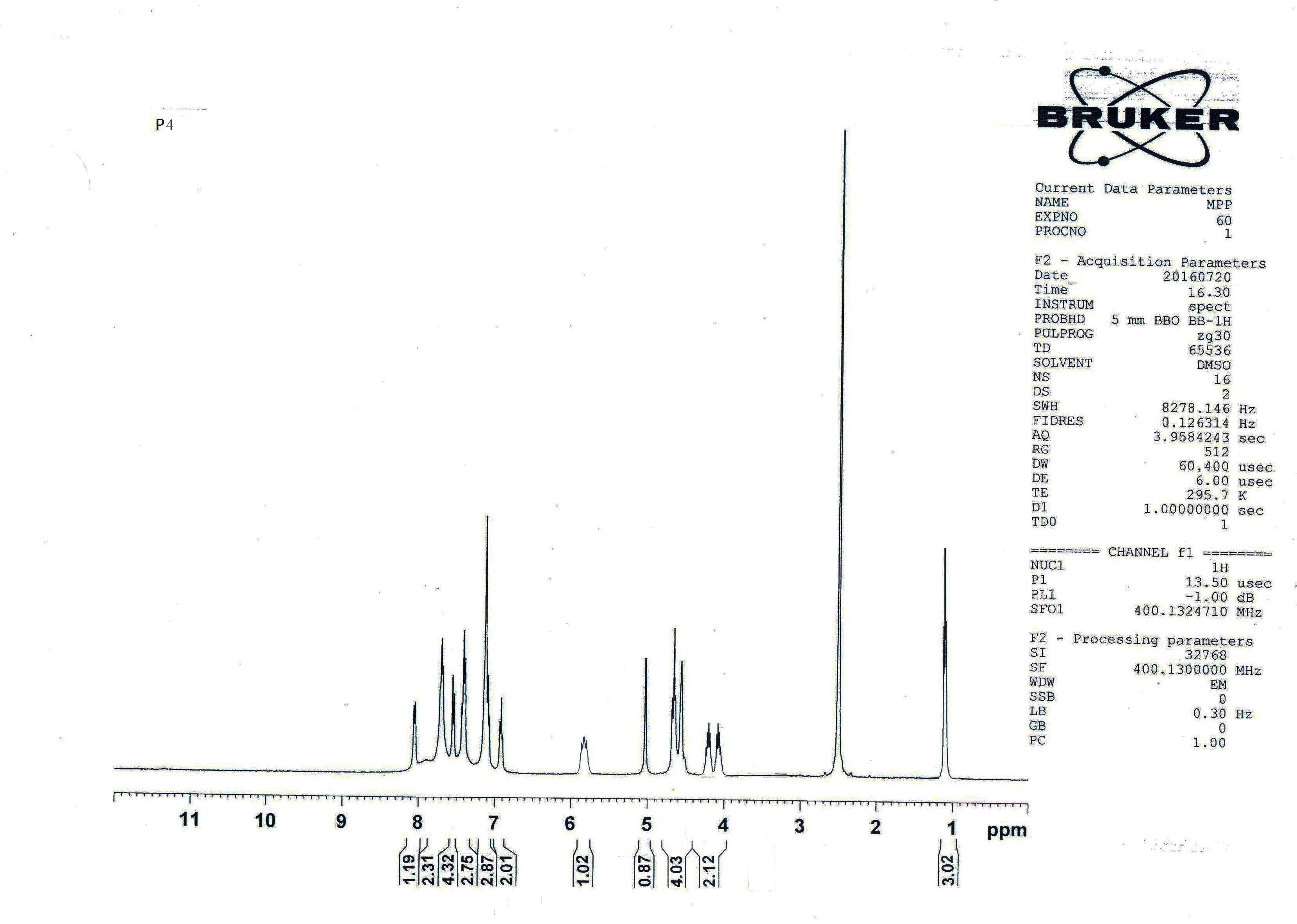
**

^1^H NMR spectra of compound **P_5_**

**
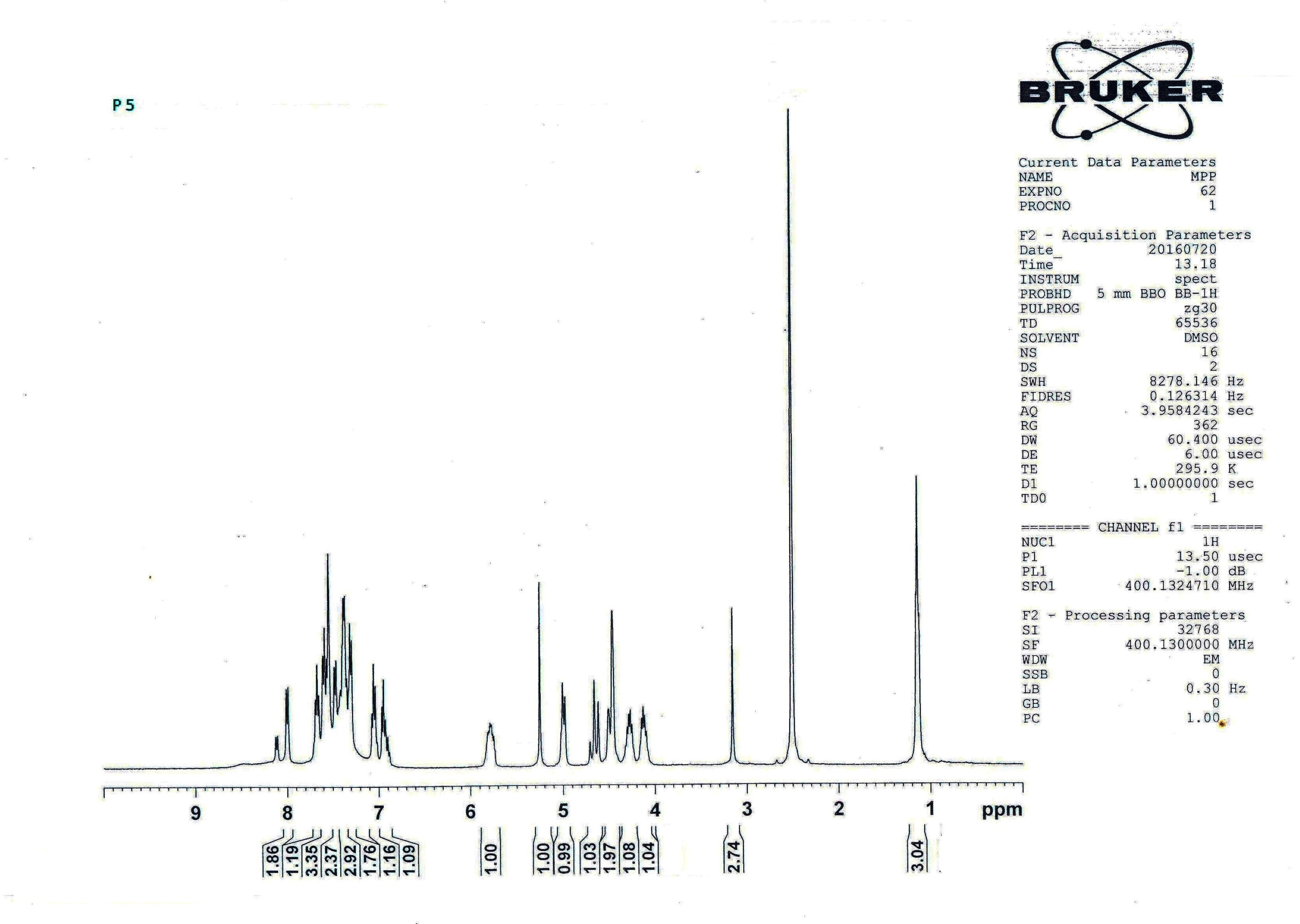
**

^1^H NMR spectra of compound **P_6_**

**
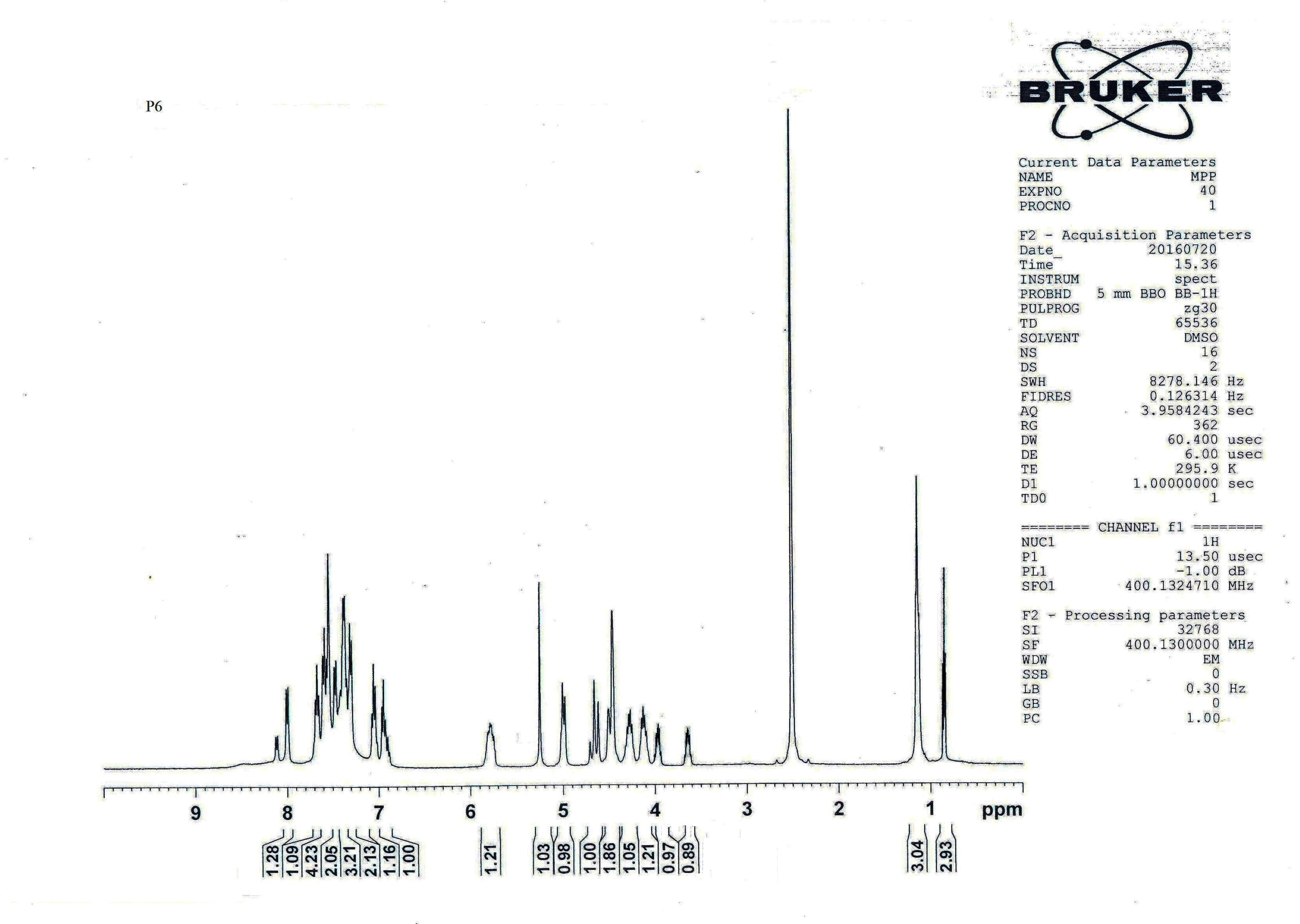
**

^1^H NMR spectra of compound **P_7_**

**
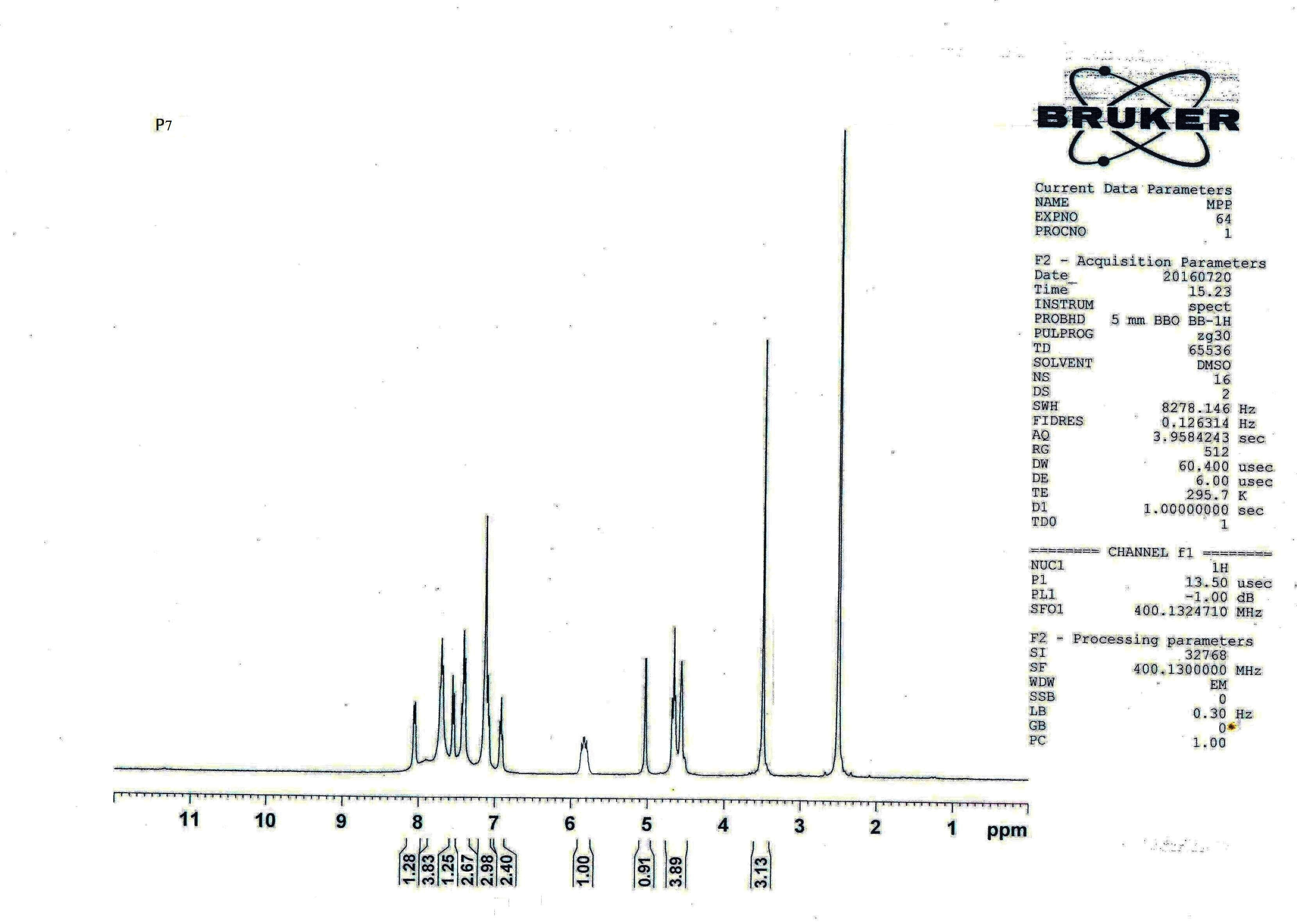
**

^1^H NMR spectra of compound **P_8_**

**
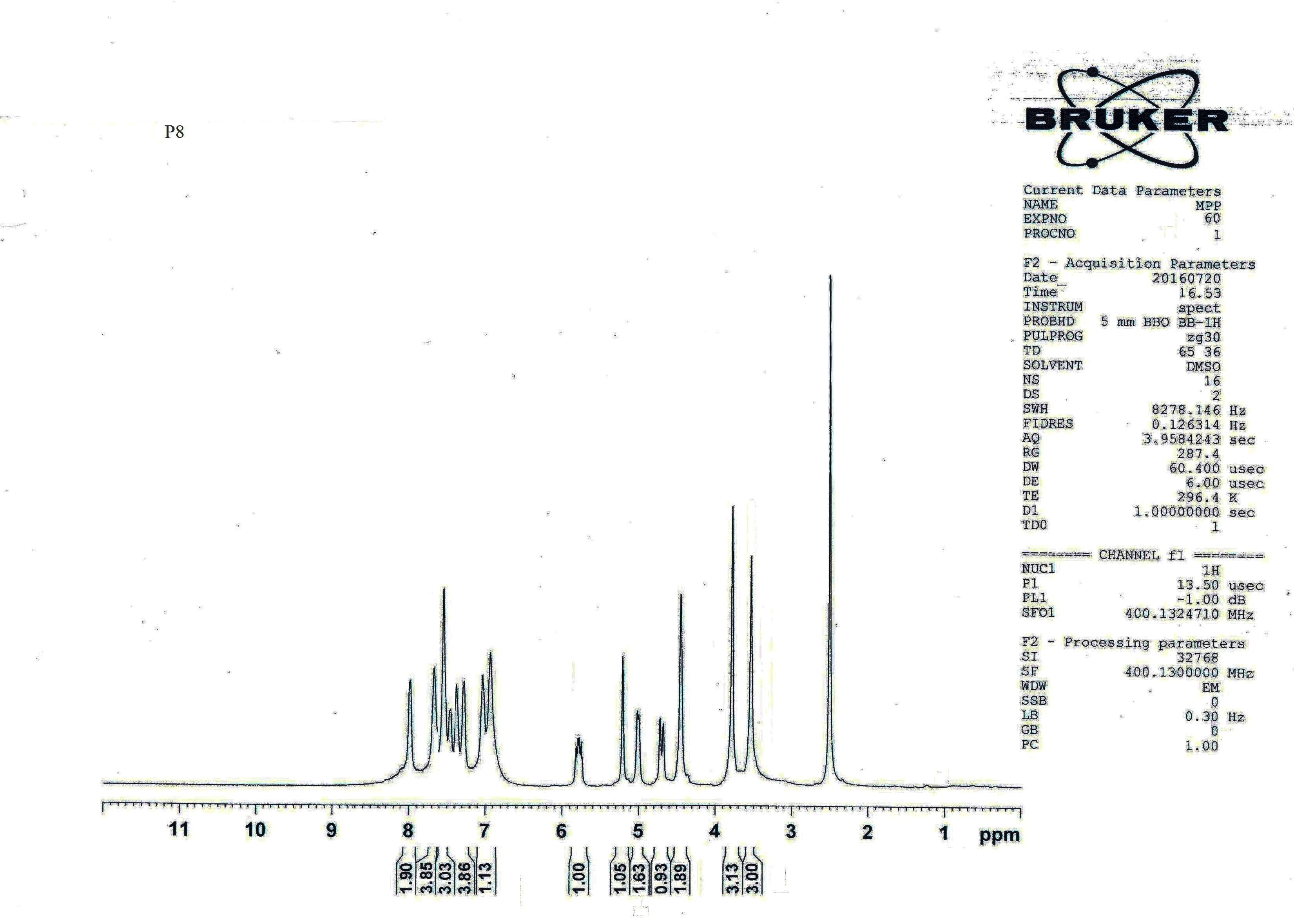
**

^1^H NMR spectra of compound **P_9_**

**
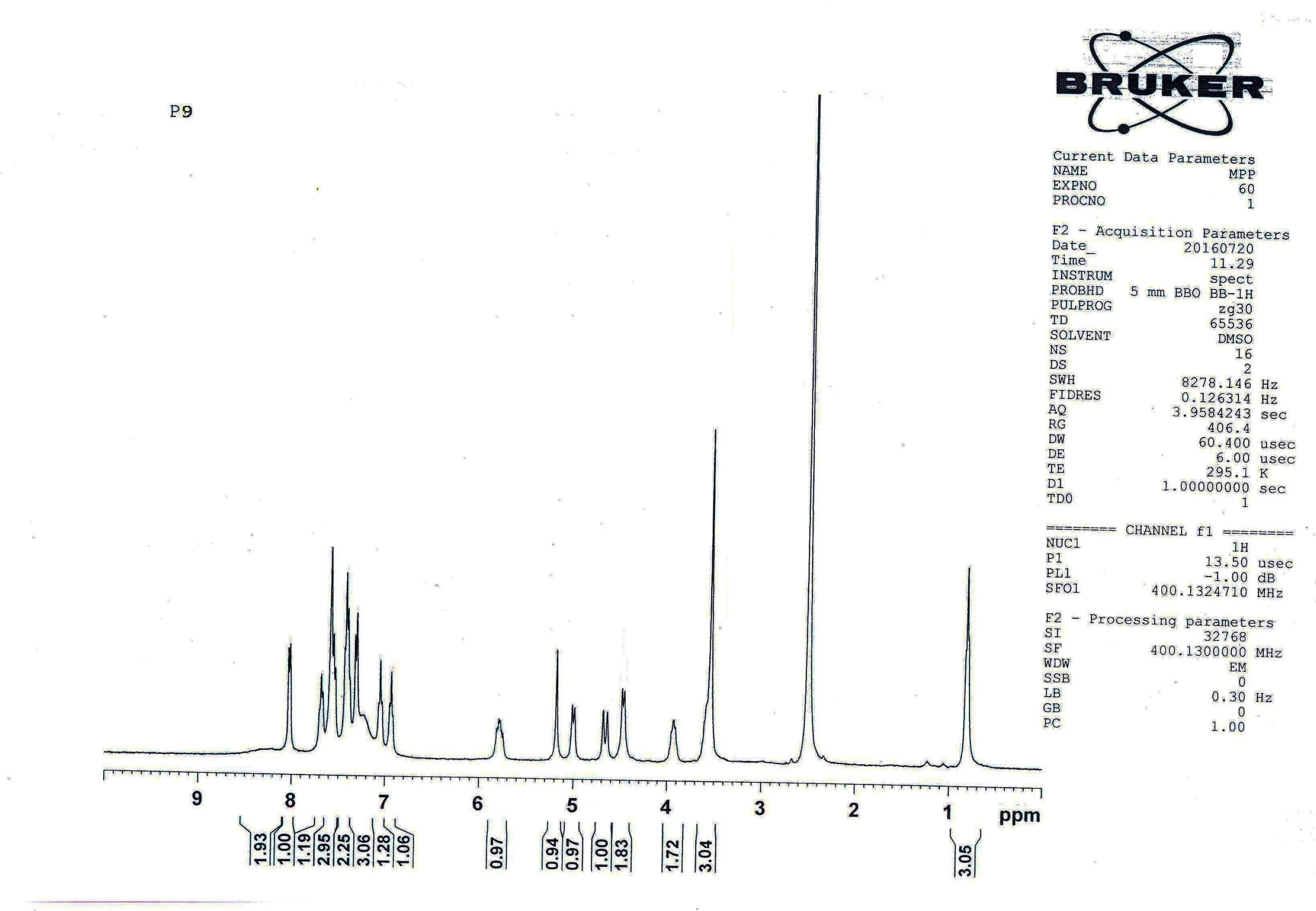
**

^1^H NMR spectra of compound **P_10_**

**
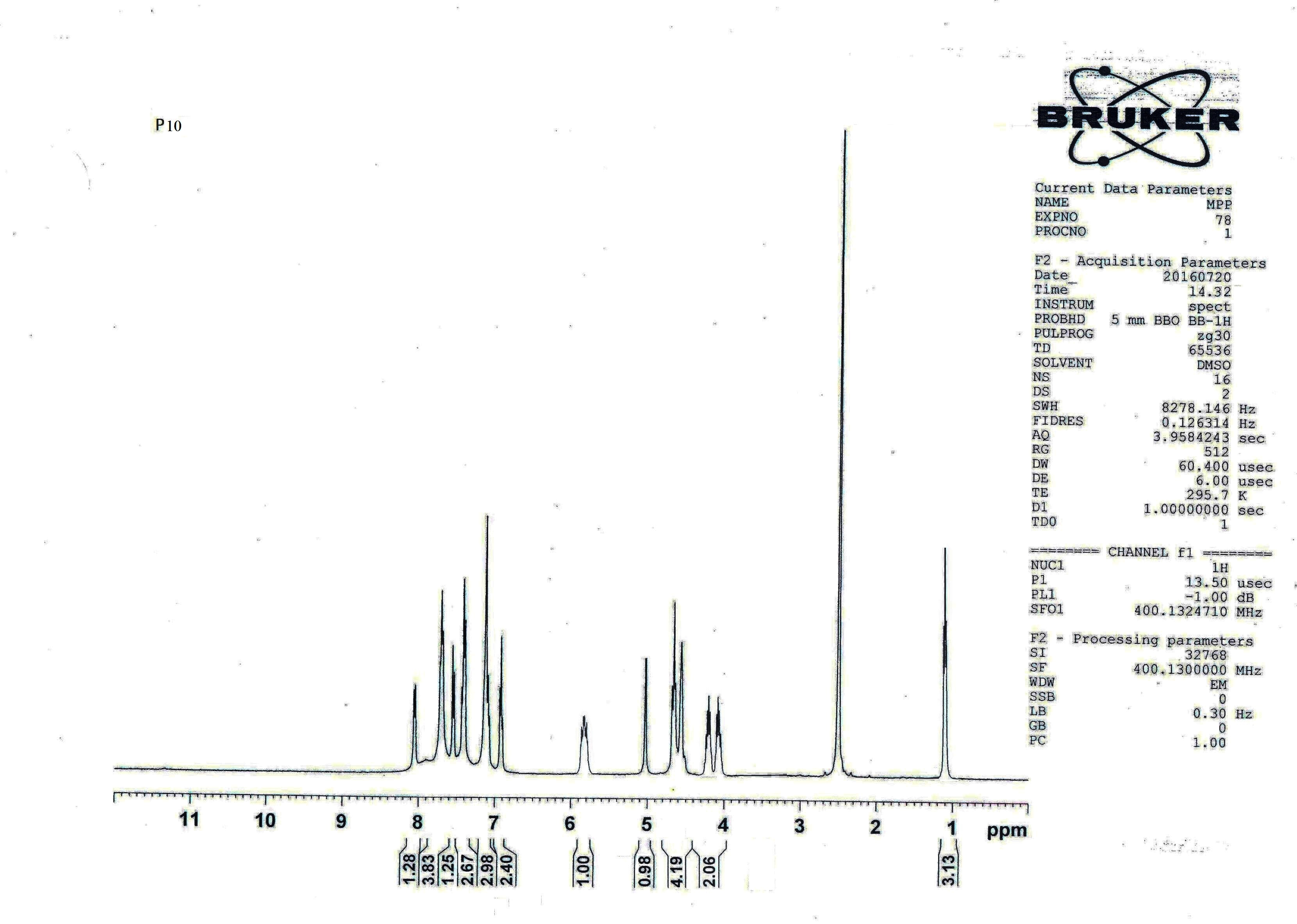
**

^1^H NMR spectra of compound **P_11_**

**
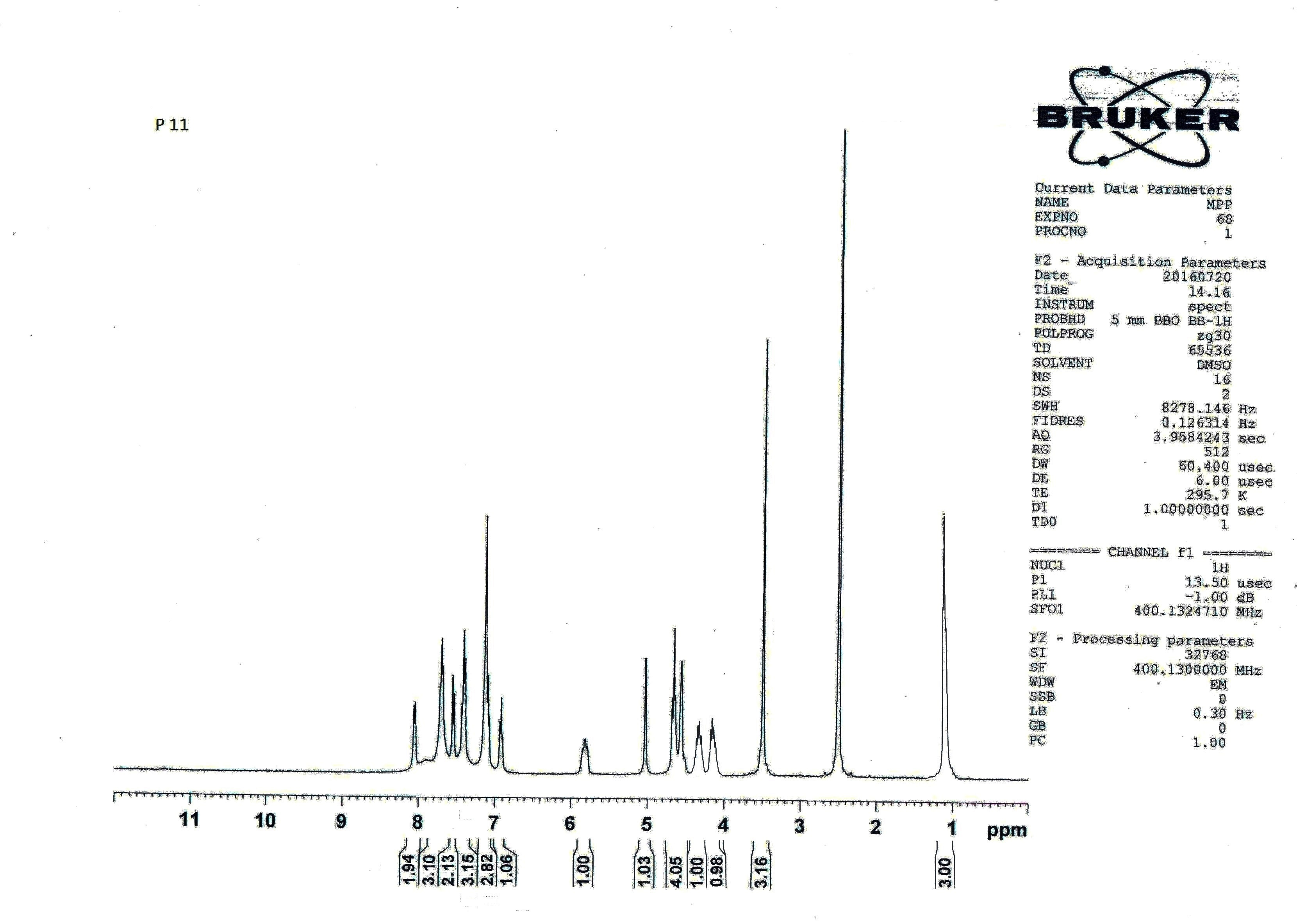
**

^1^H NMR spectra of compound **P_12_**

**
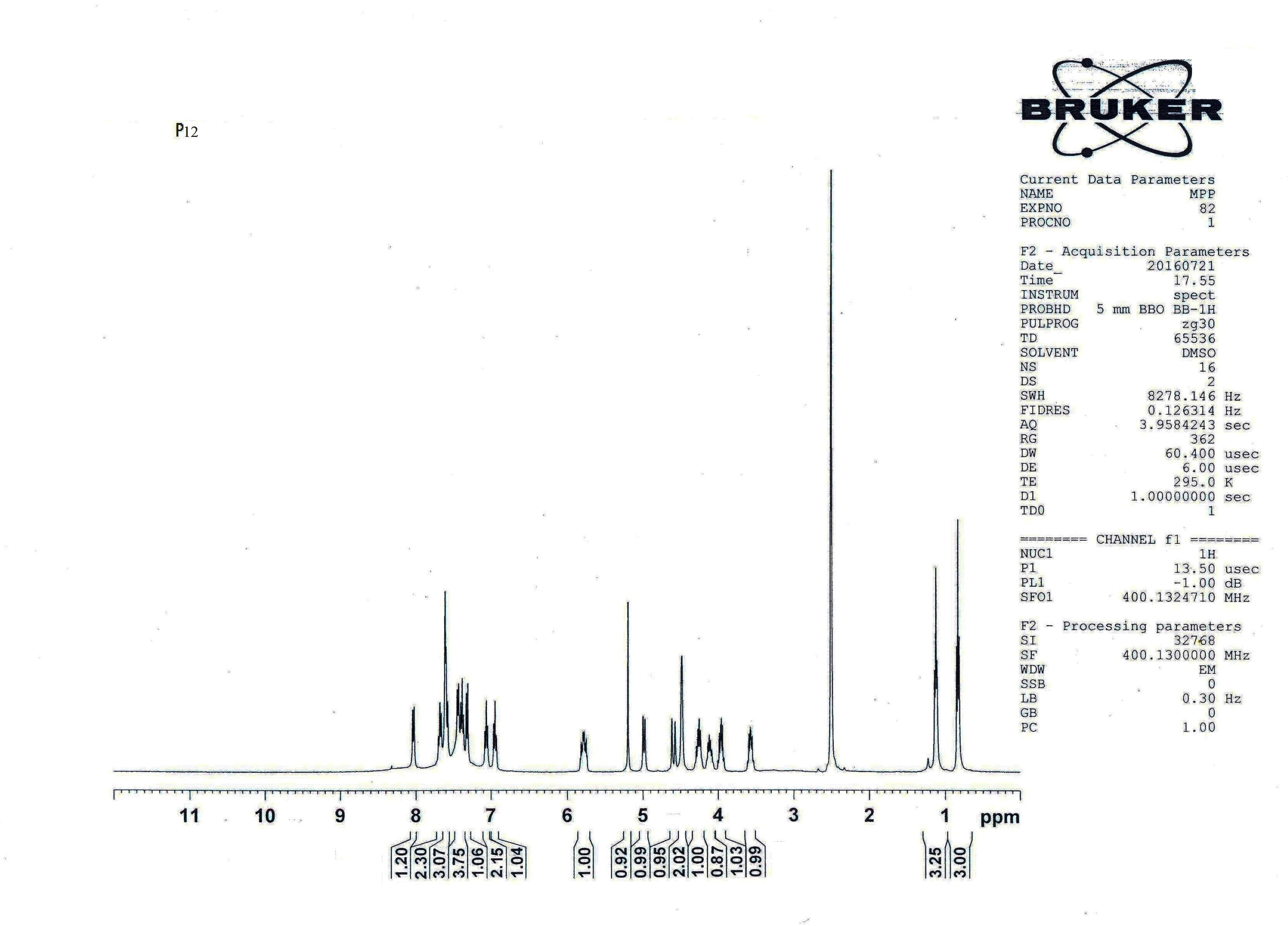
**

^1^H NMR spectra of compound **P_13_**

**
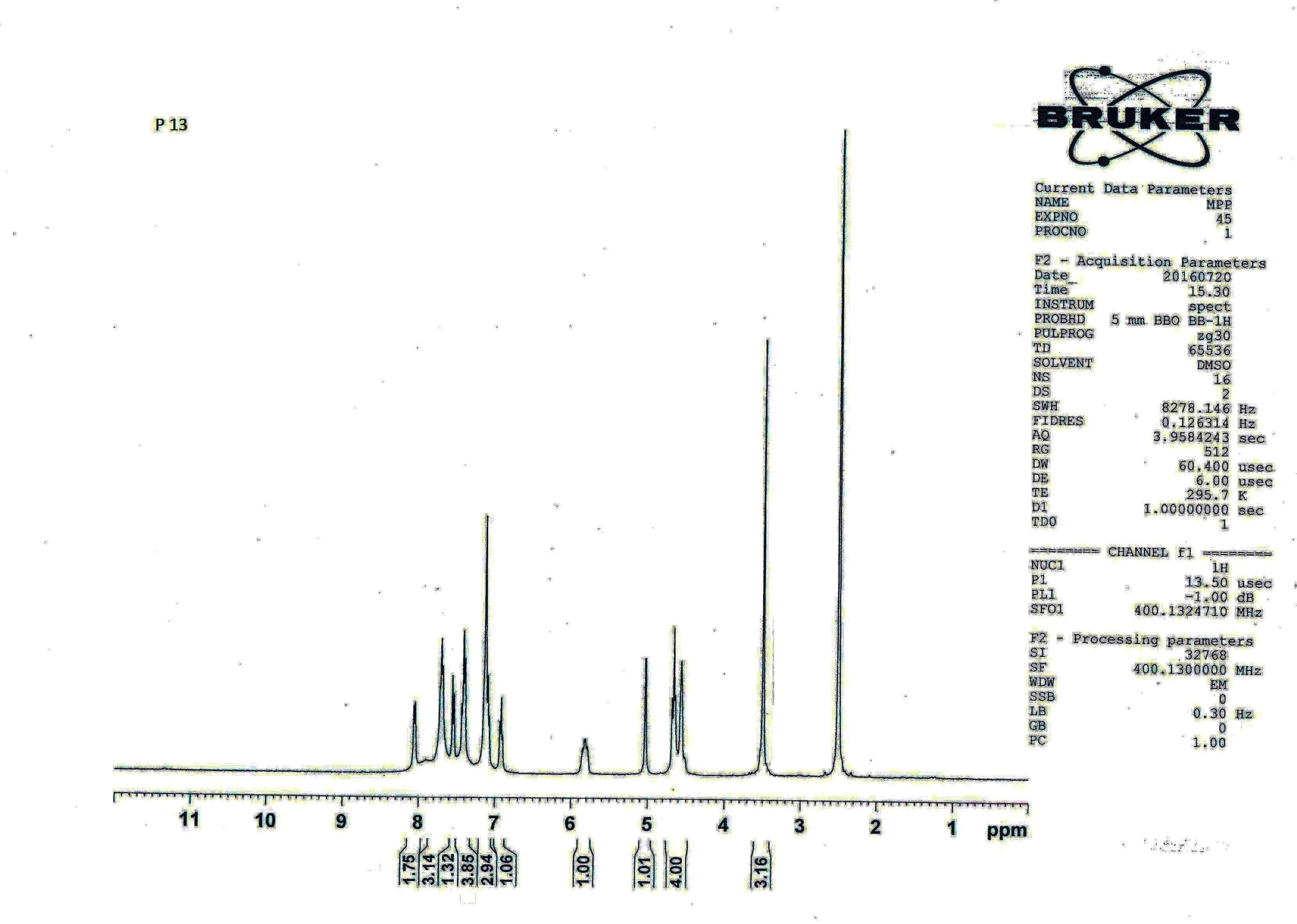
**

^1^H NMR spectra of compound **P_14_**


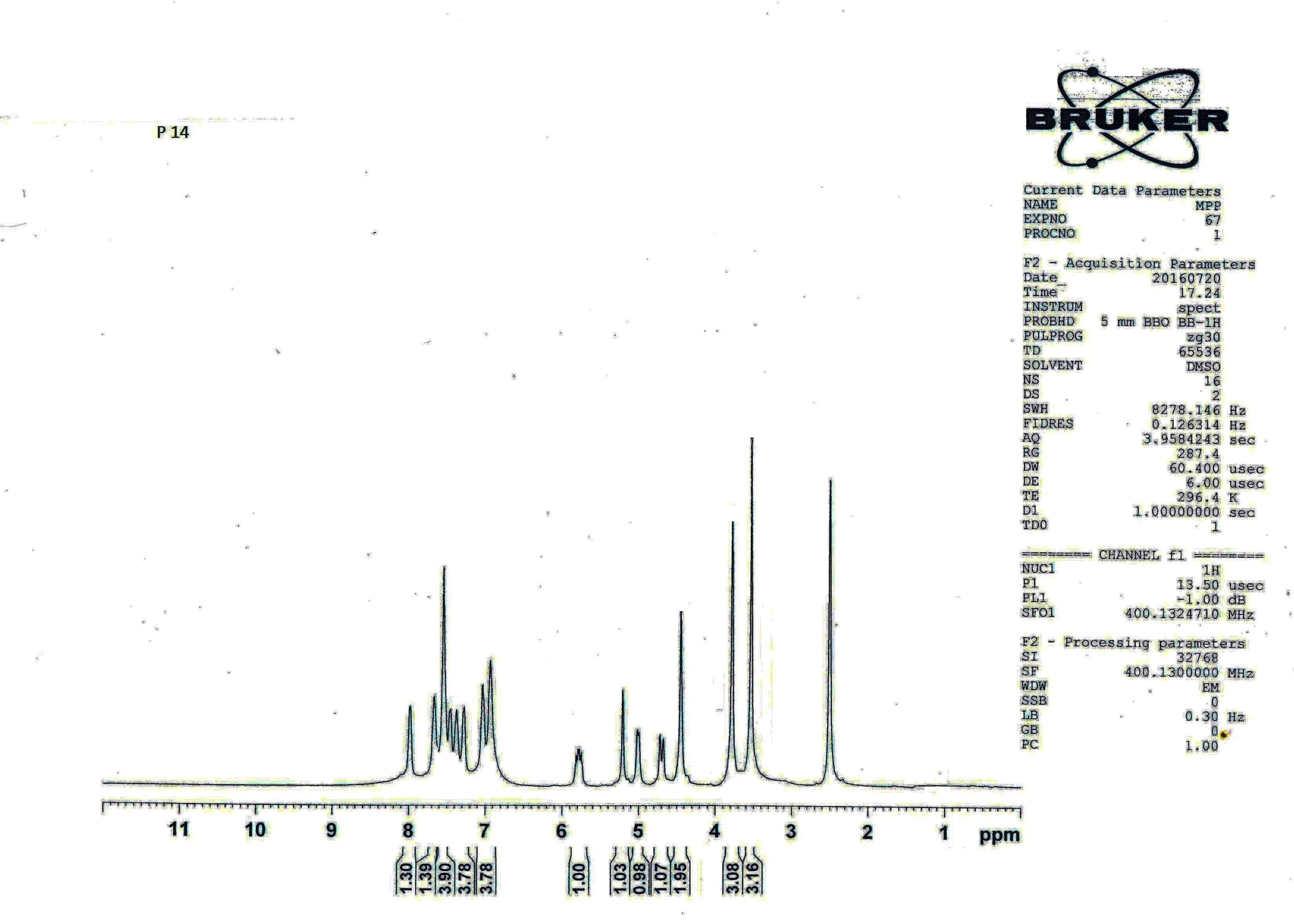


^1^H NMR spectra of compound **P_15_**


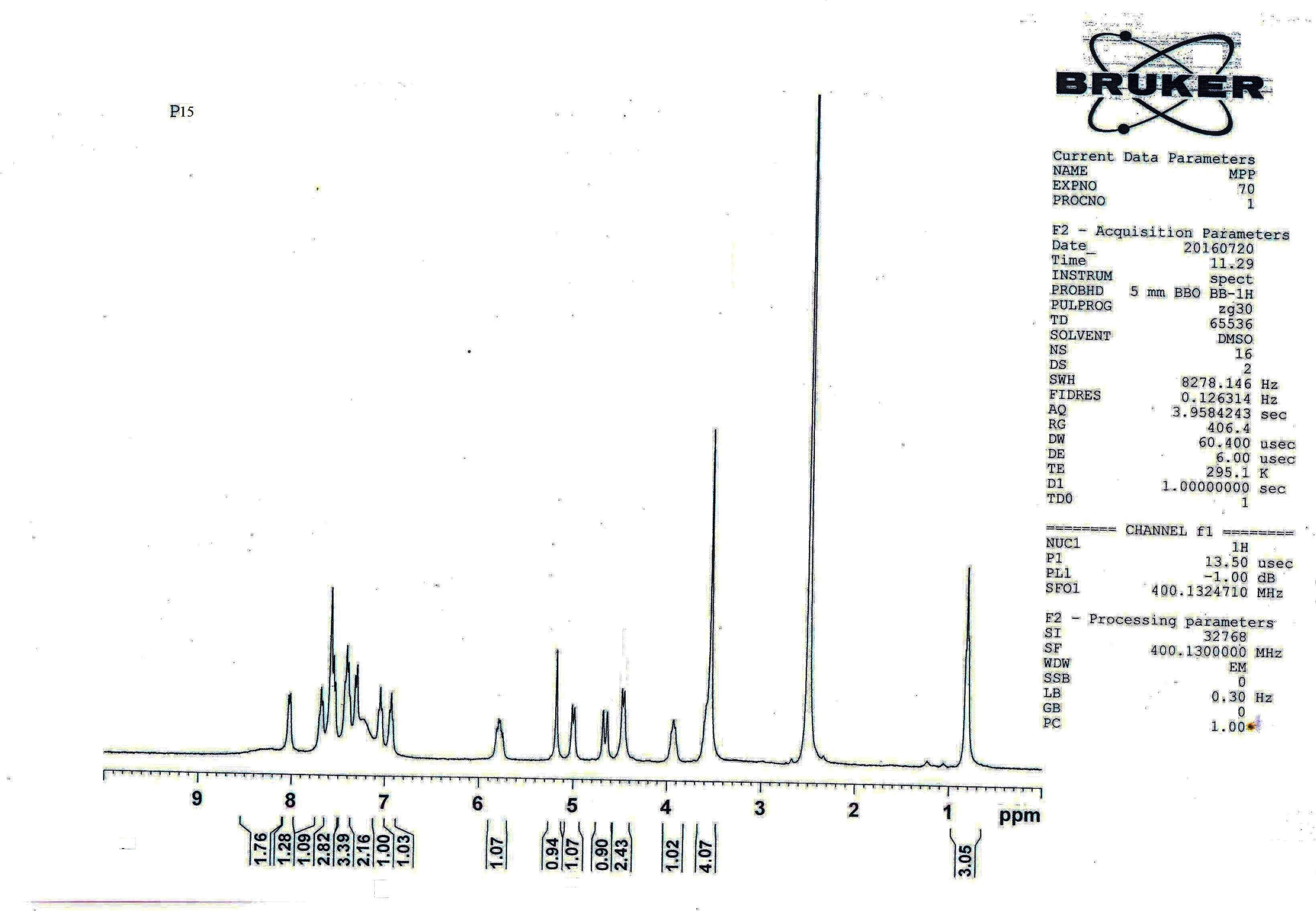


^1^H NMR spectra of compound **P_16_**

**
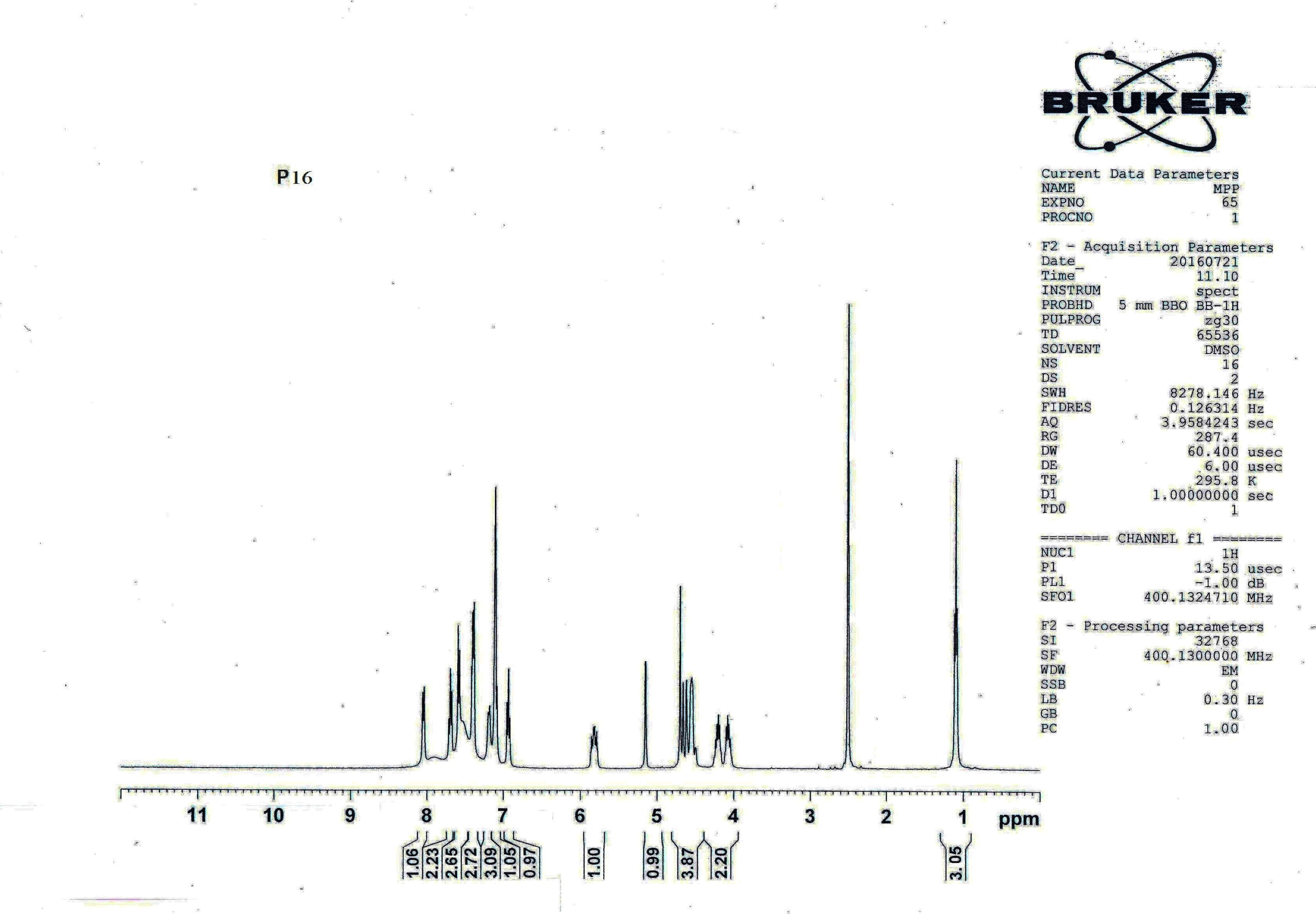
**

^1^H NMR spectra of compound **P_17_**

**
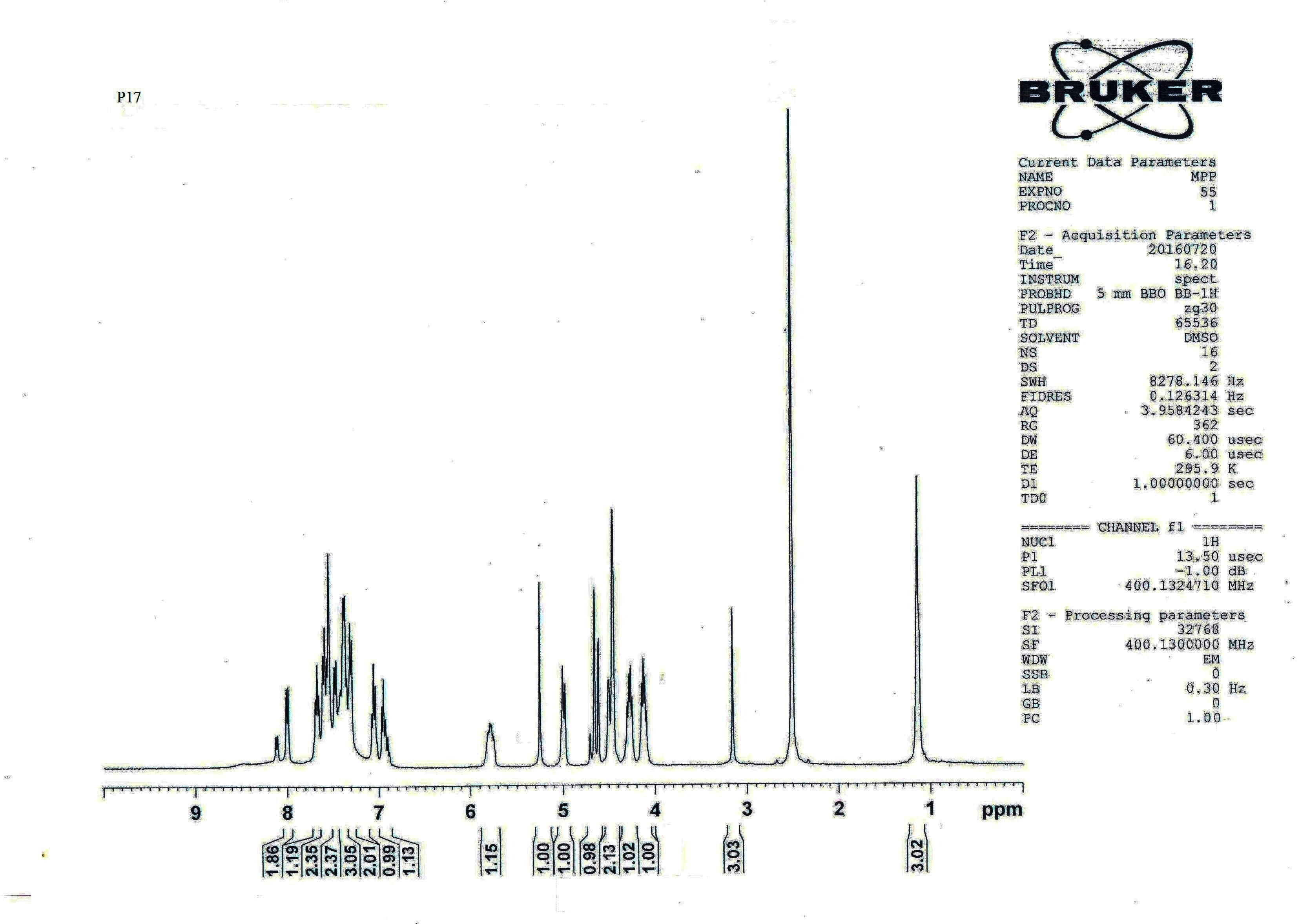
**

^1^H NMR spectra of compound **P_18_**

**
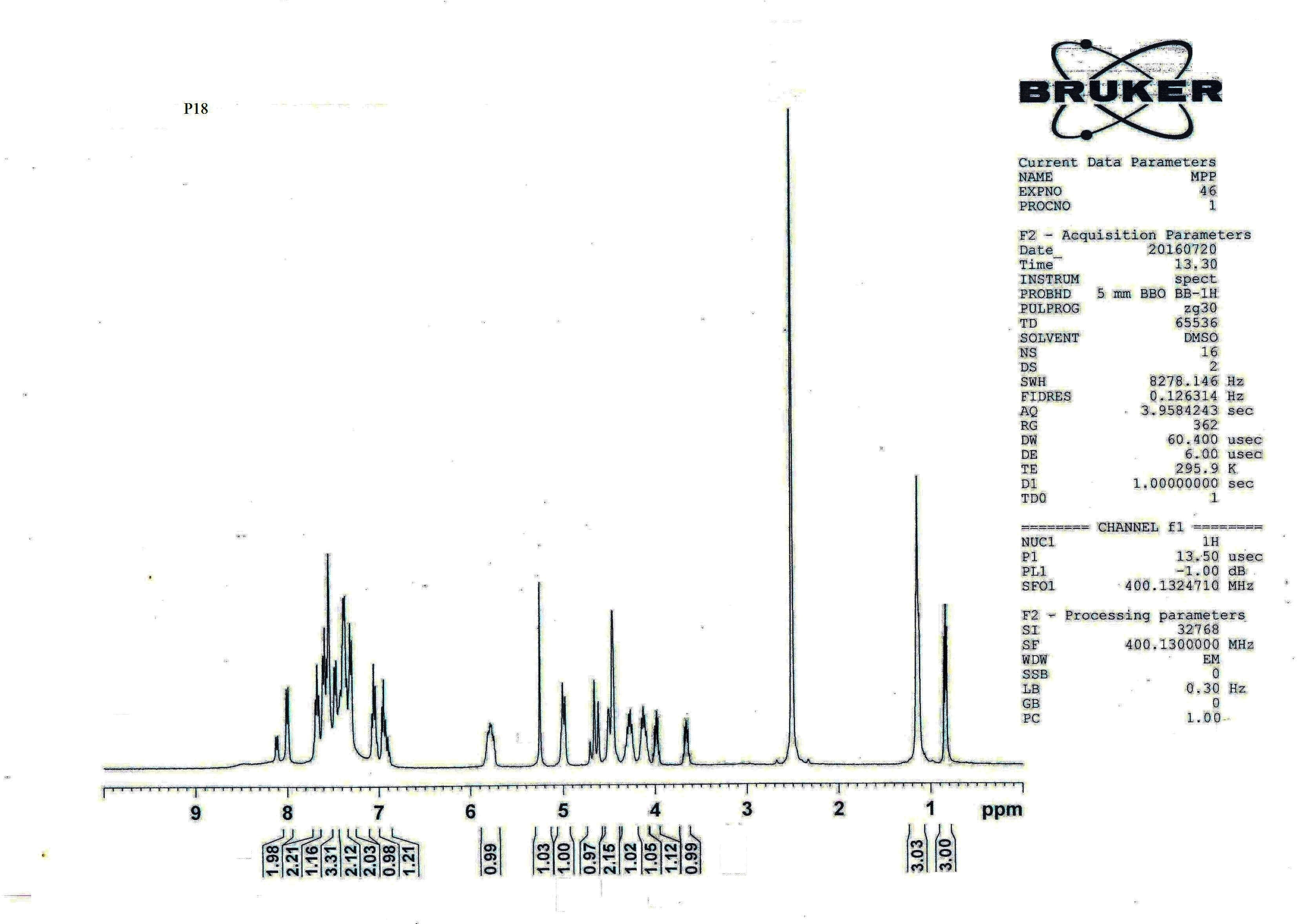
**

^1^H NMR spectra of compound **P_19_**


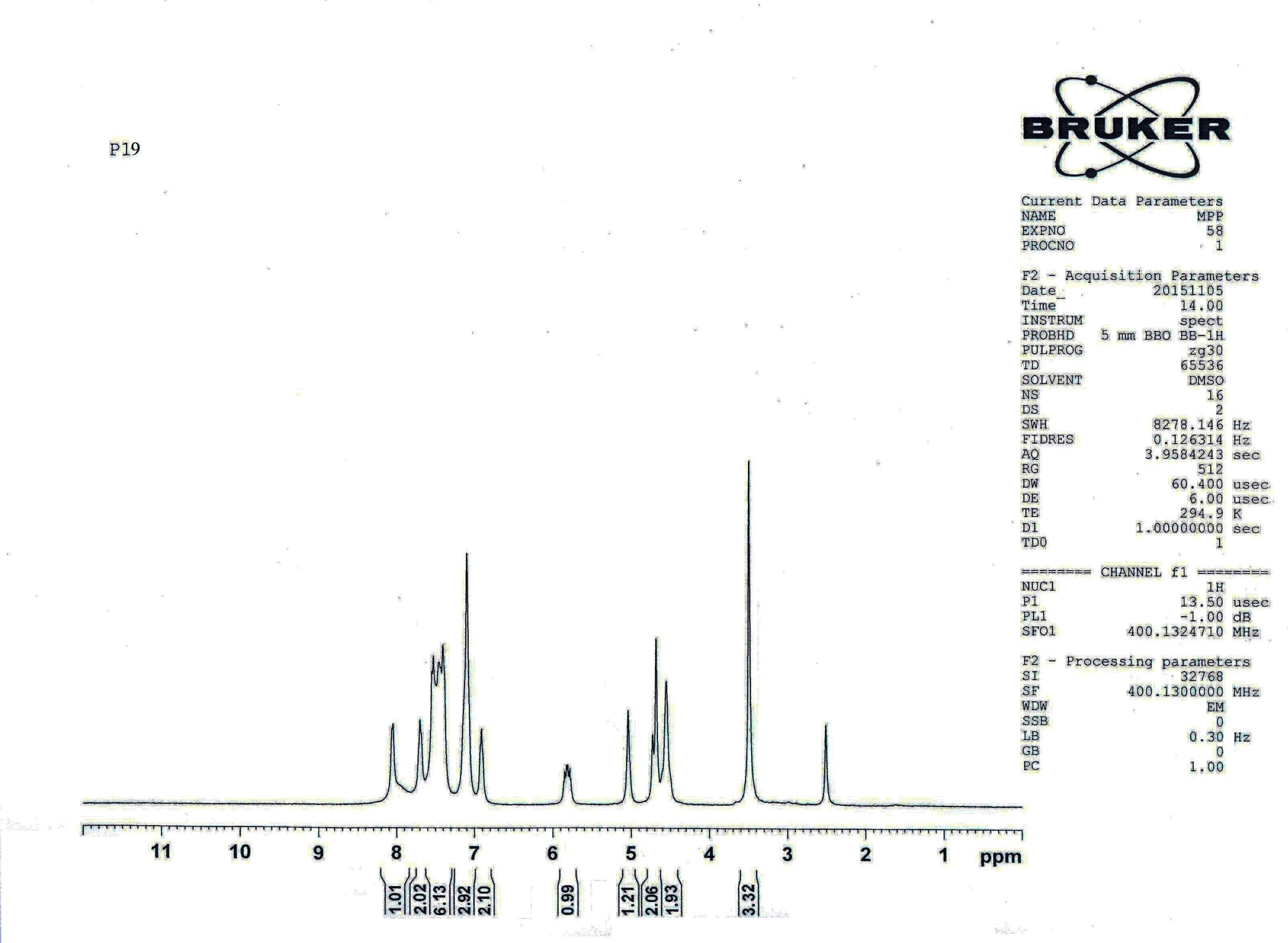


^1^H NMR spectra of compound **P_20_**


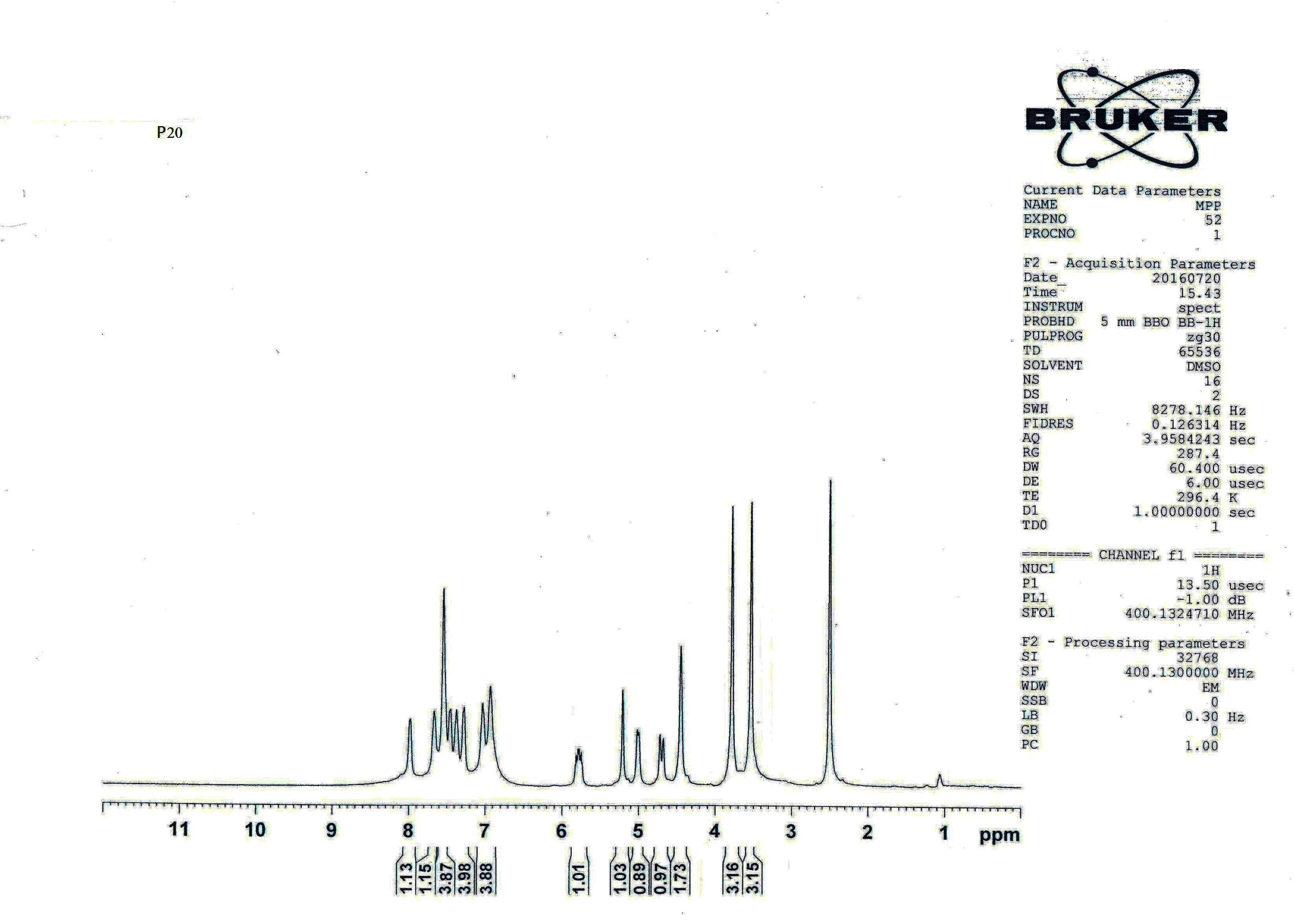


^1^H NMR spectra of compound **P_21_**


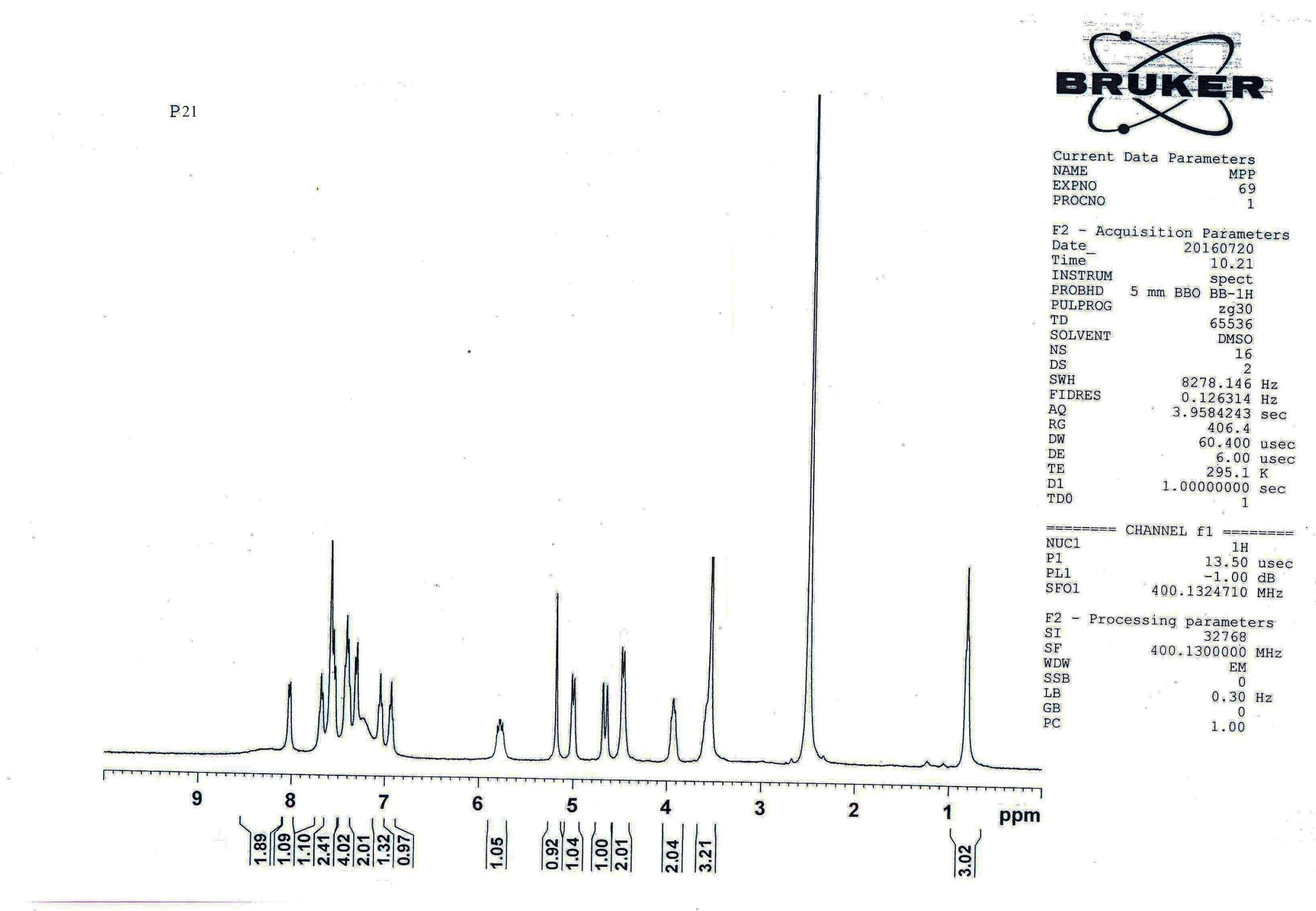


^1^H NMR spectra of compound **P_22_**

**
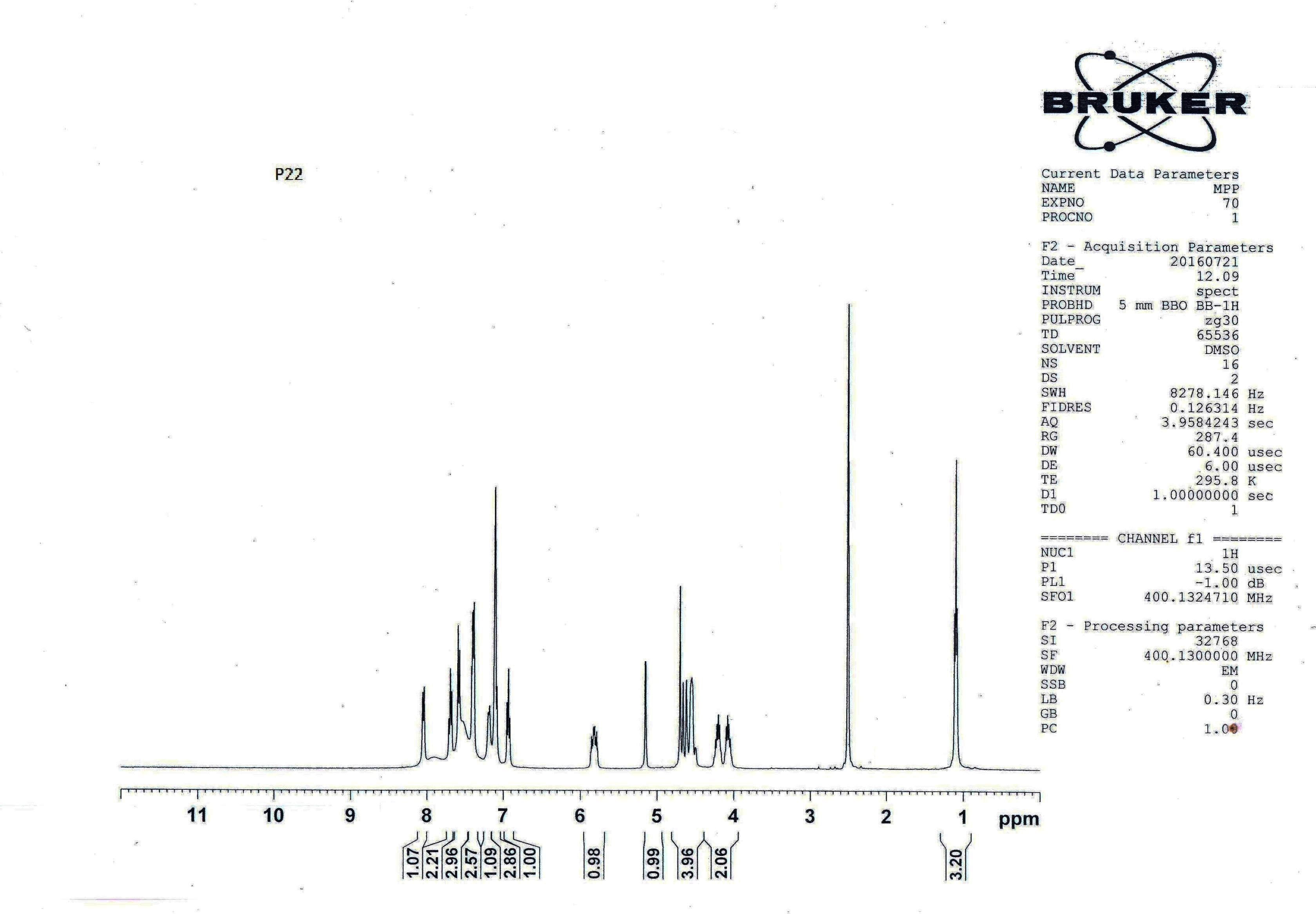
**

^1^H NMR spectra of compound **P_23_**


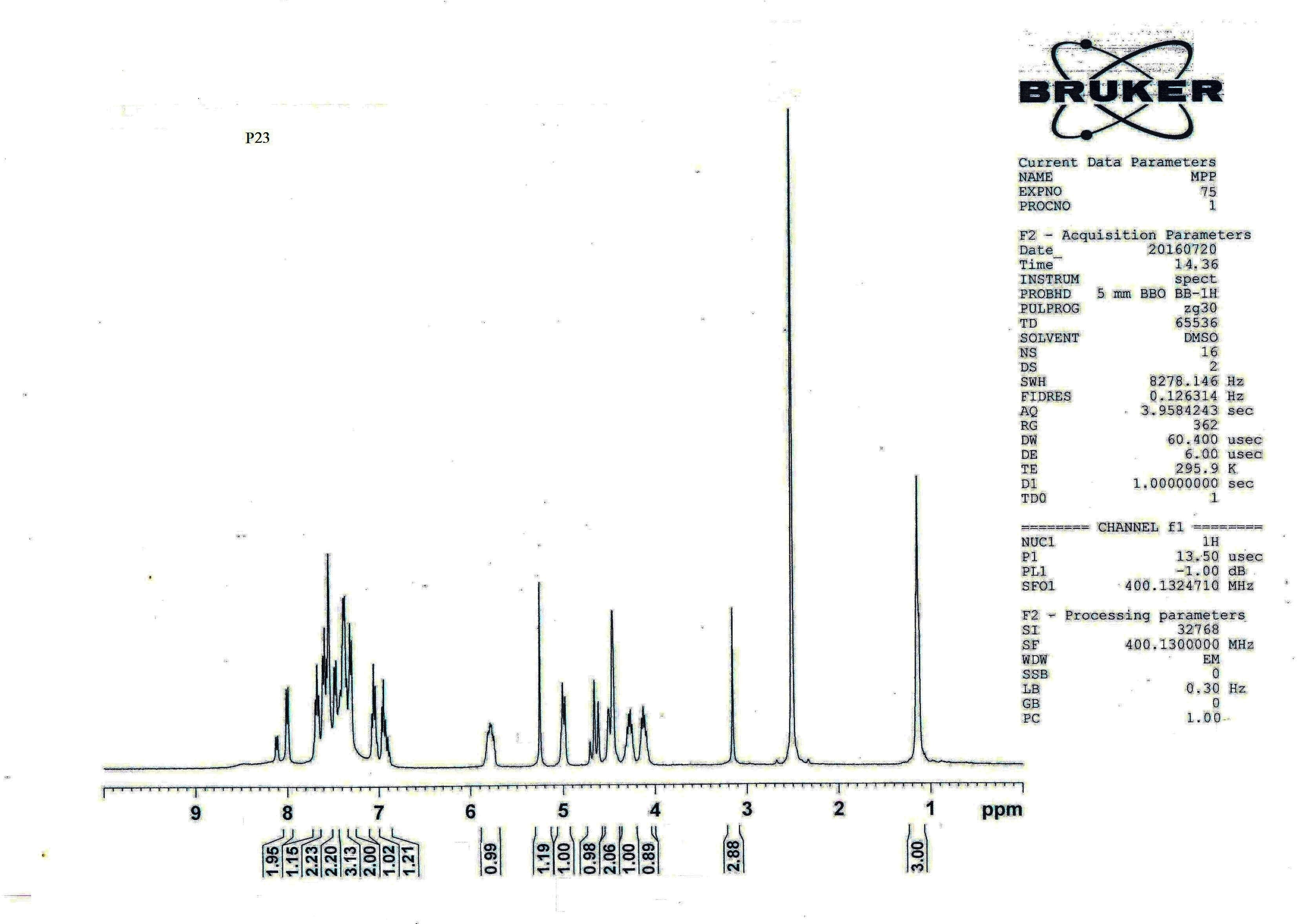


^1^H NMR spectra of compound **P_24_**

**
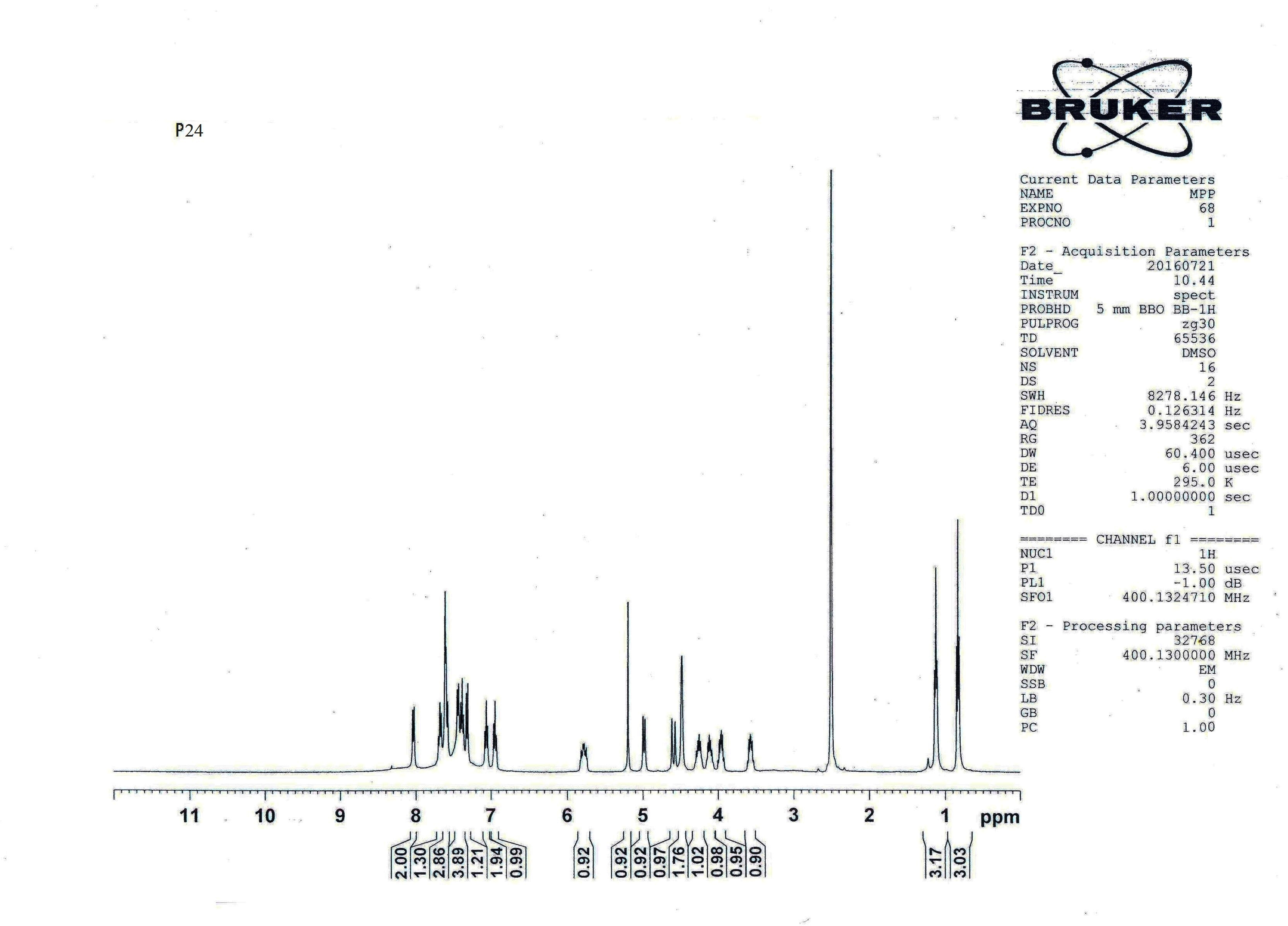
**

^13^C NMR spectra of compound **P_1_**


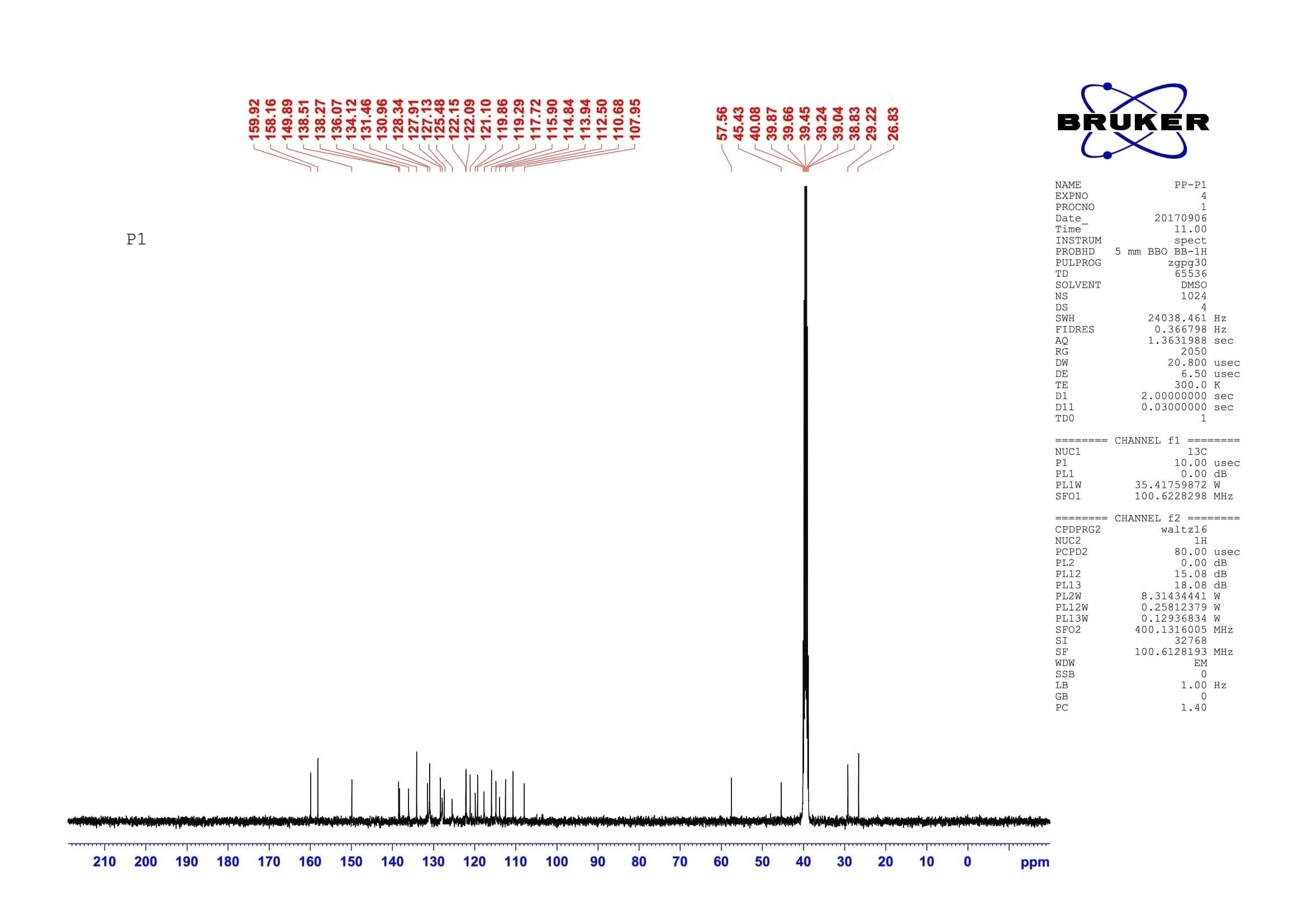


^13^C NMR spectra of compound **P_2_**

**
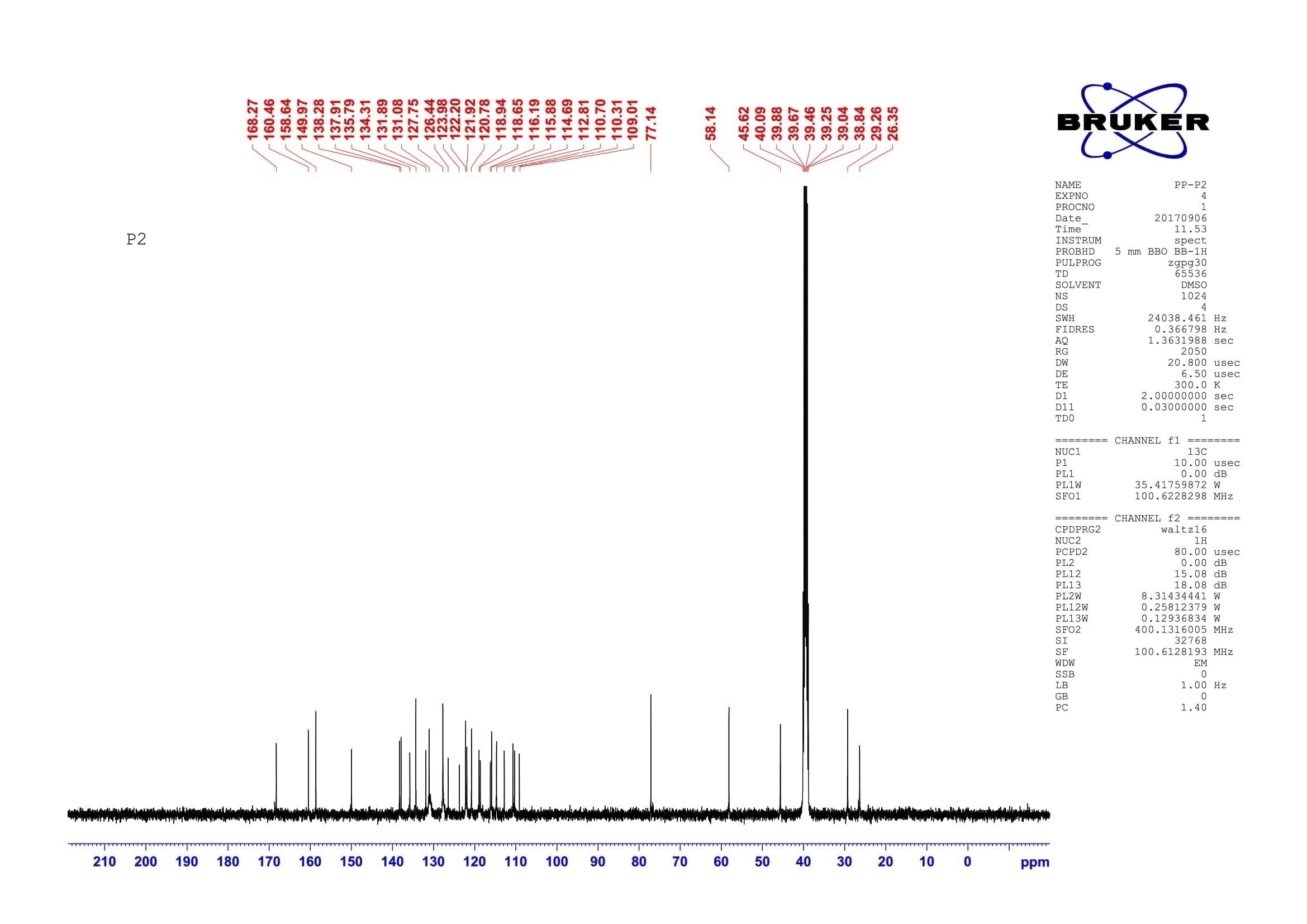
**

^13^C NMR spectra of compound **P_3_**

**
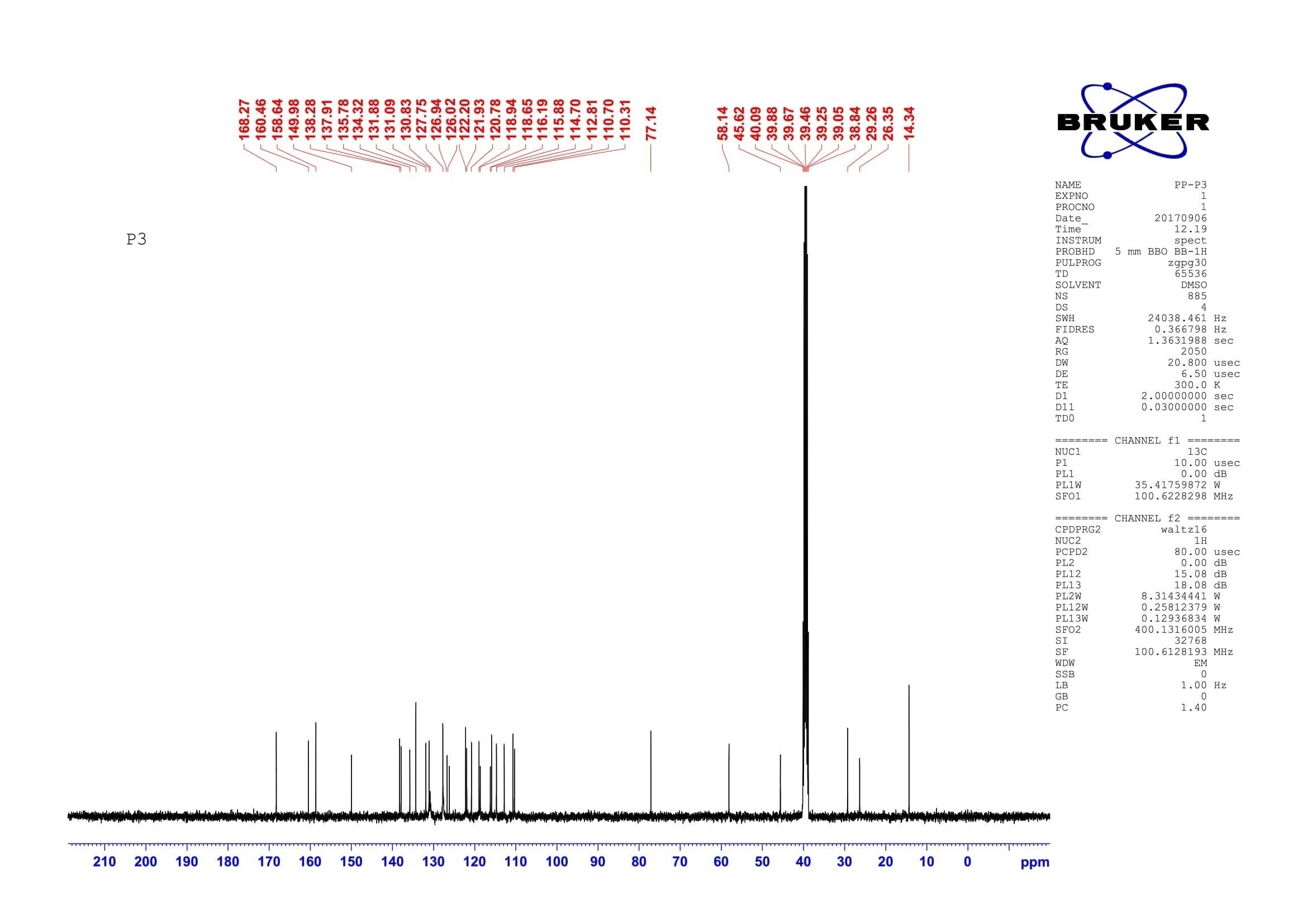
**

^13^C NMR spectra of compound **P_4_**


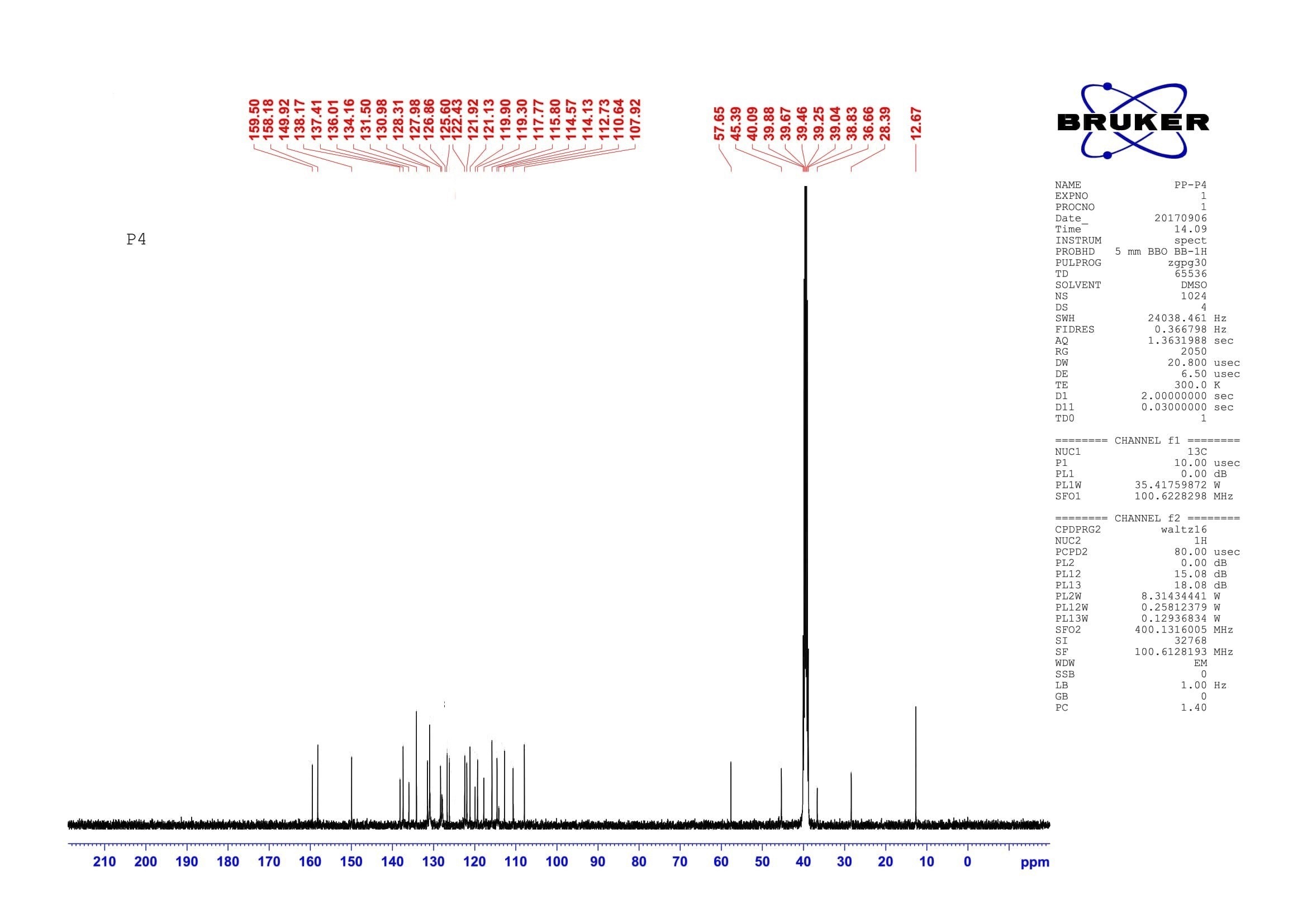


^13^C NMR spectra of compound **P_5_**


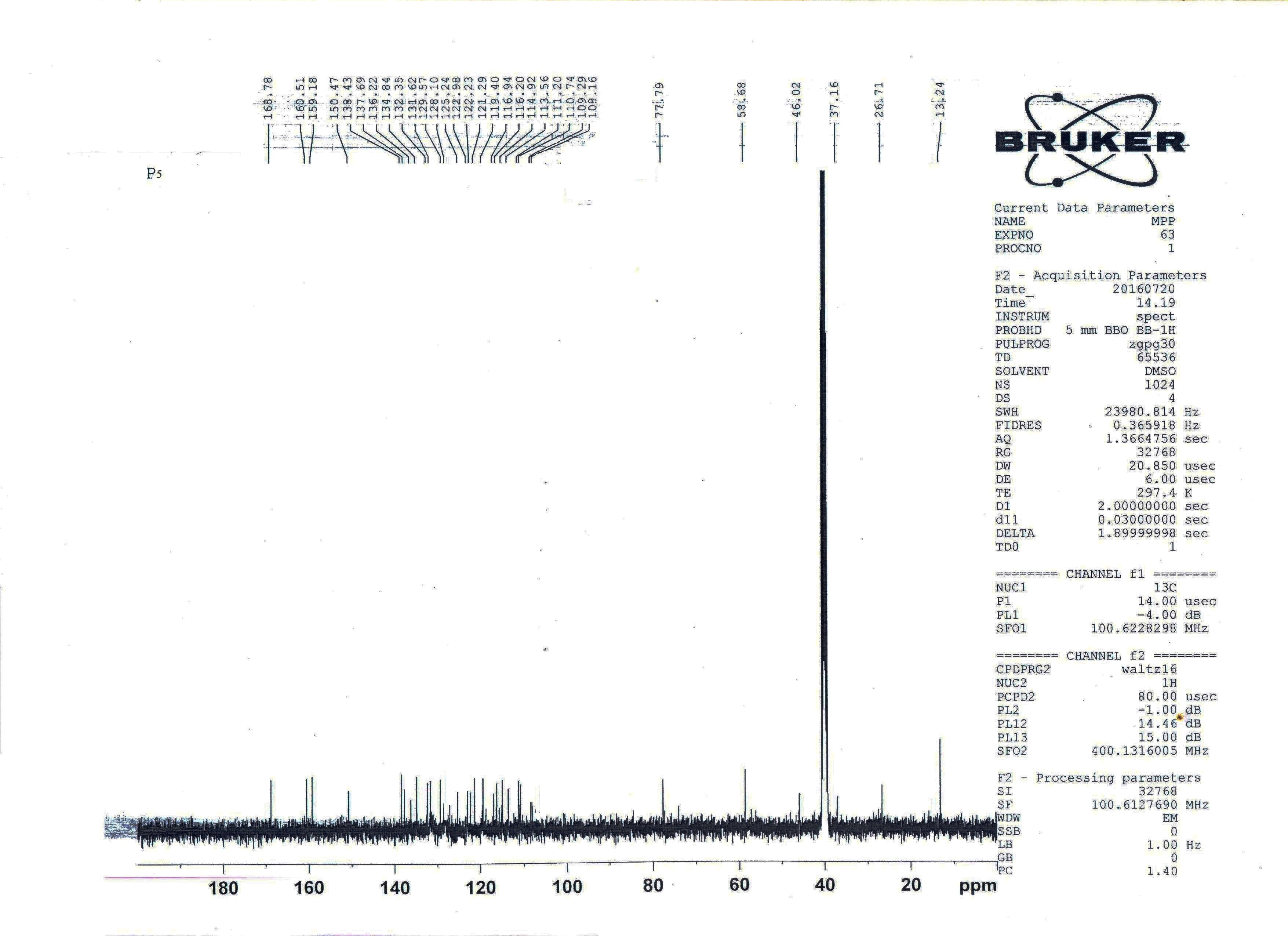


^13^C NMR spectra of compound **P_6_**

**
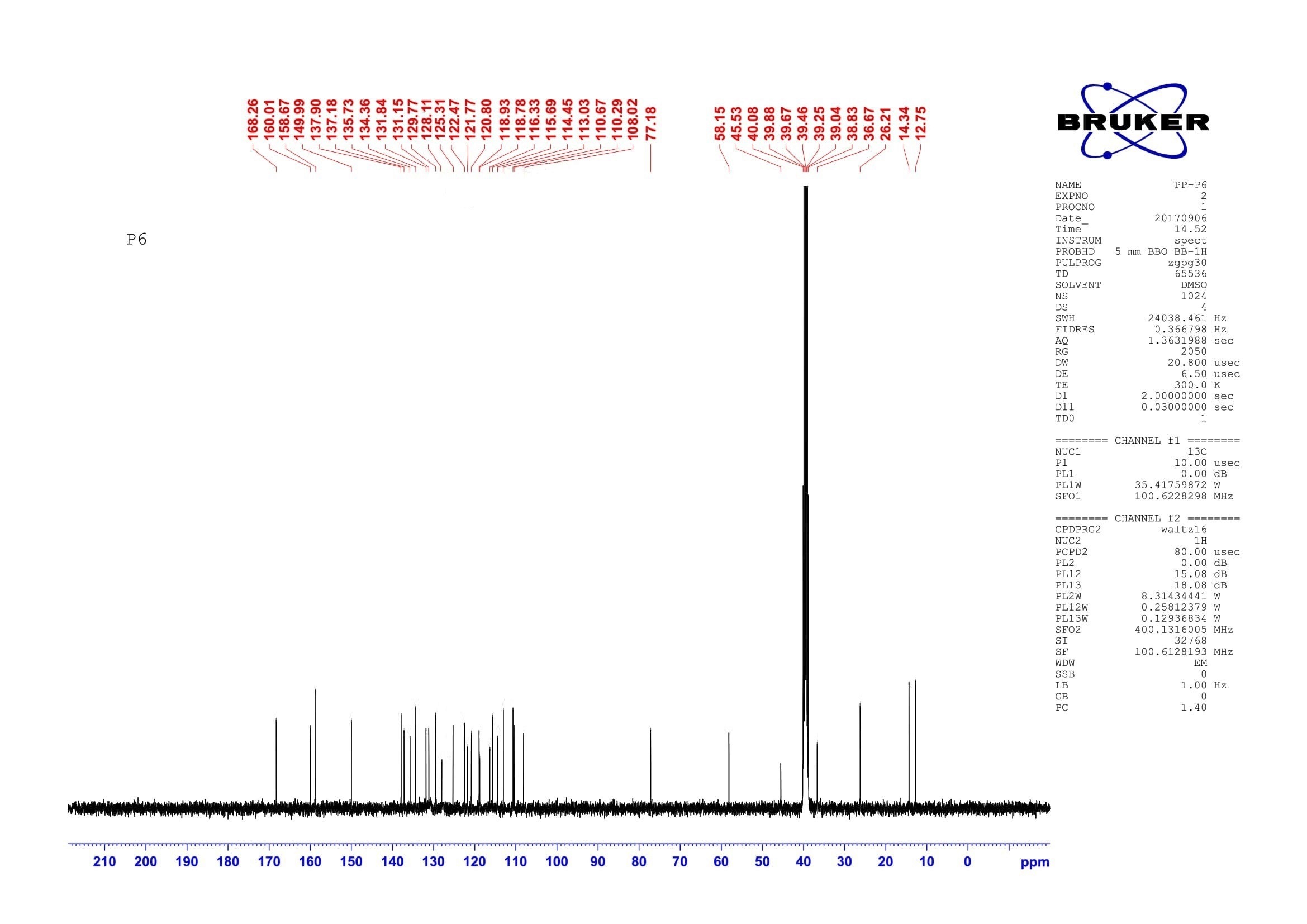
**

^13^C NMR spectra of compound **P_7_**


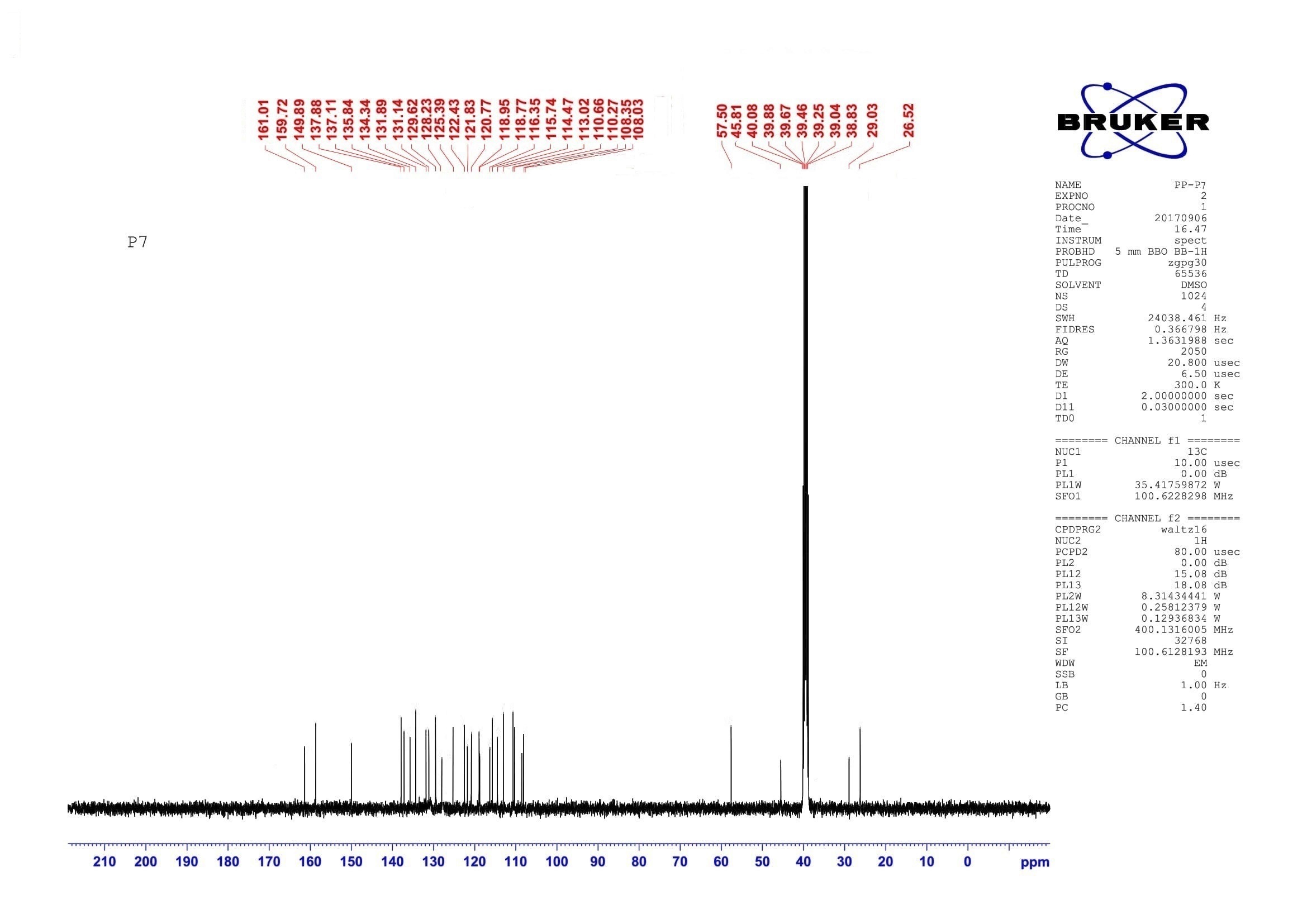


^13^C NMR spectra of compound **P_8_**


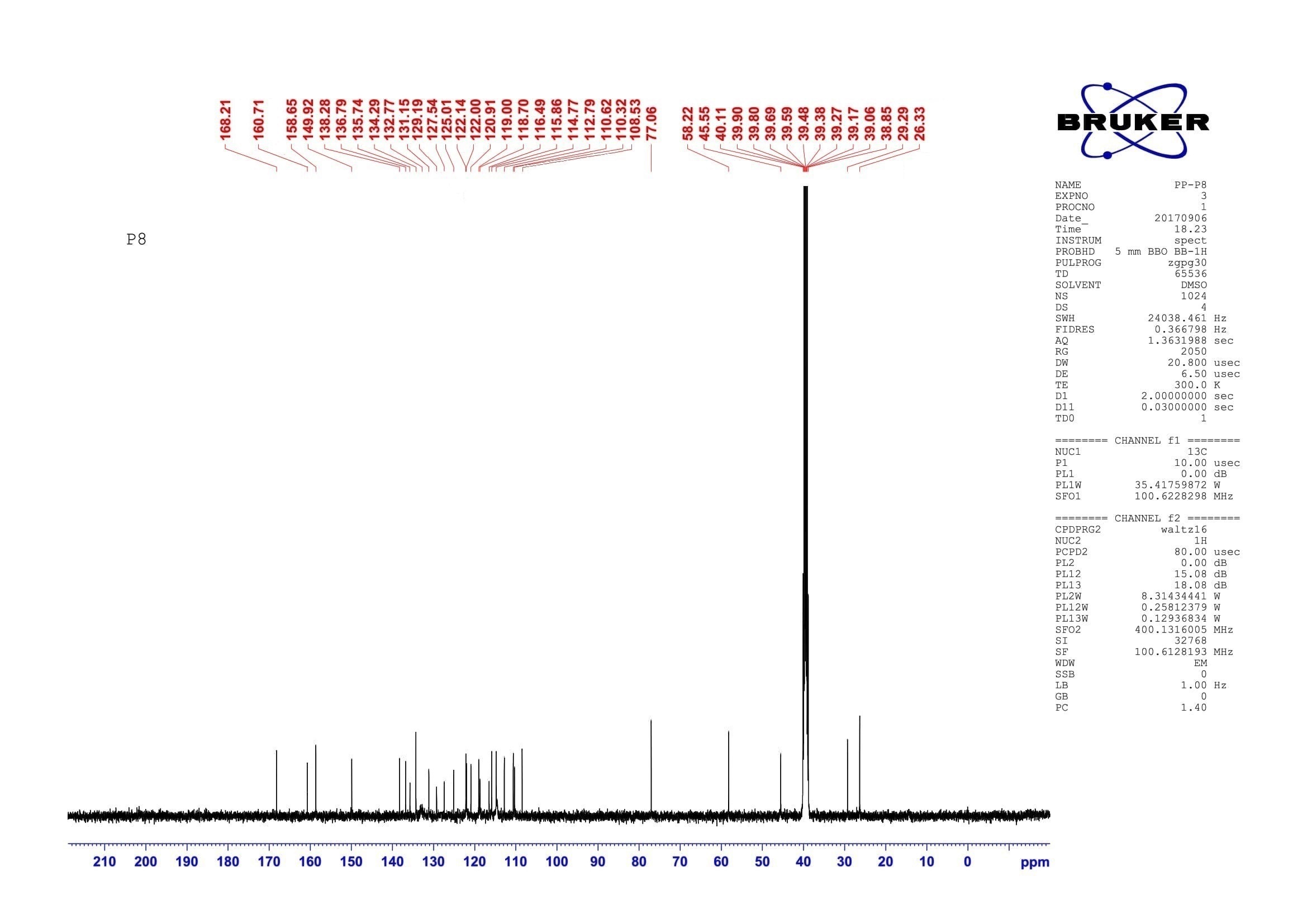


^13^C NMR spectra of compound **P_9_**


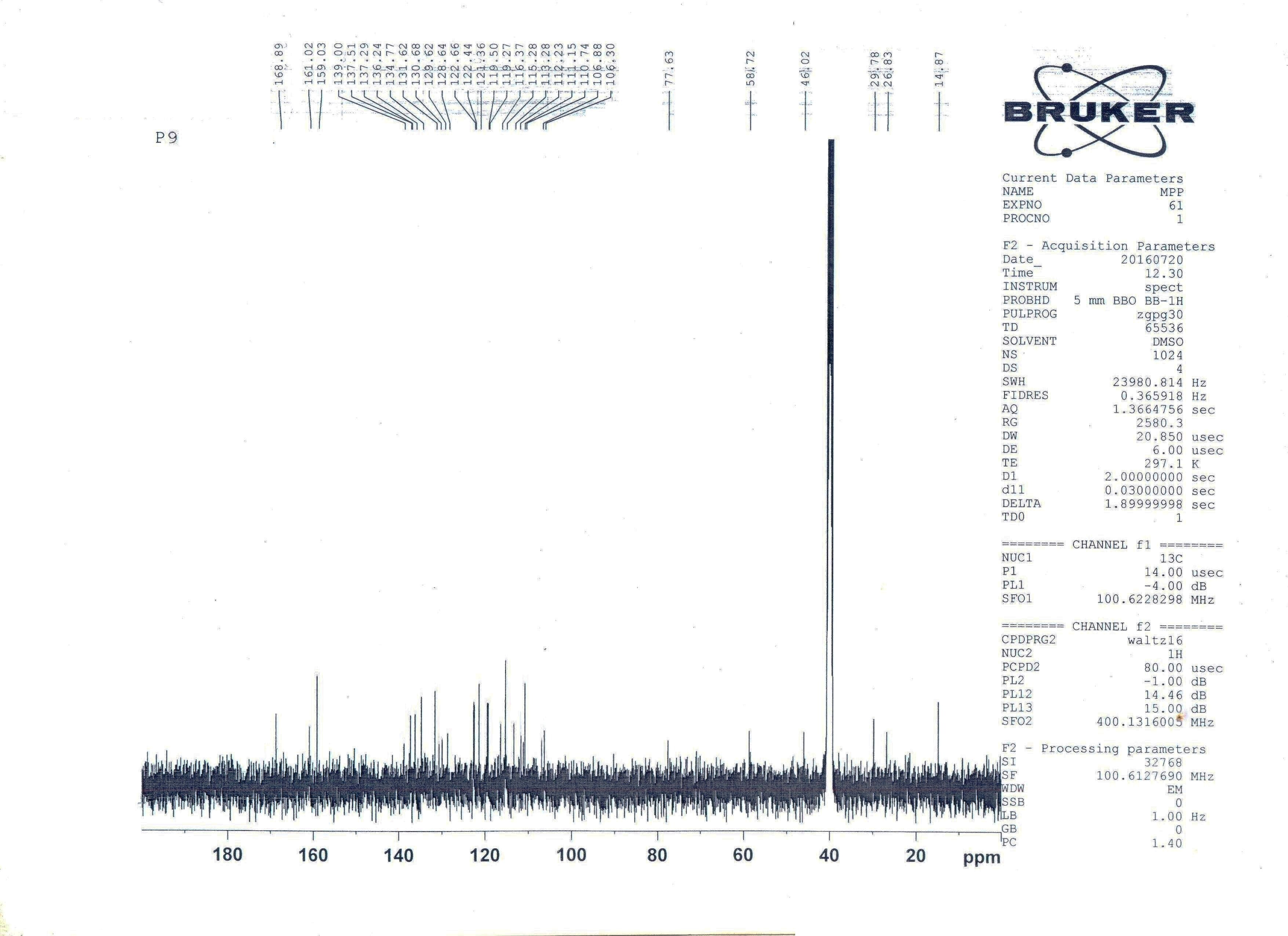


^13^C NMR spectra of compound **P_10_**

**
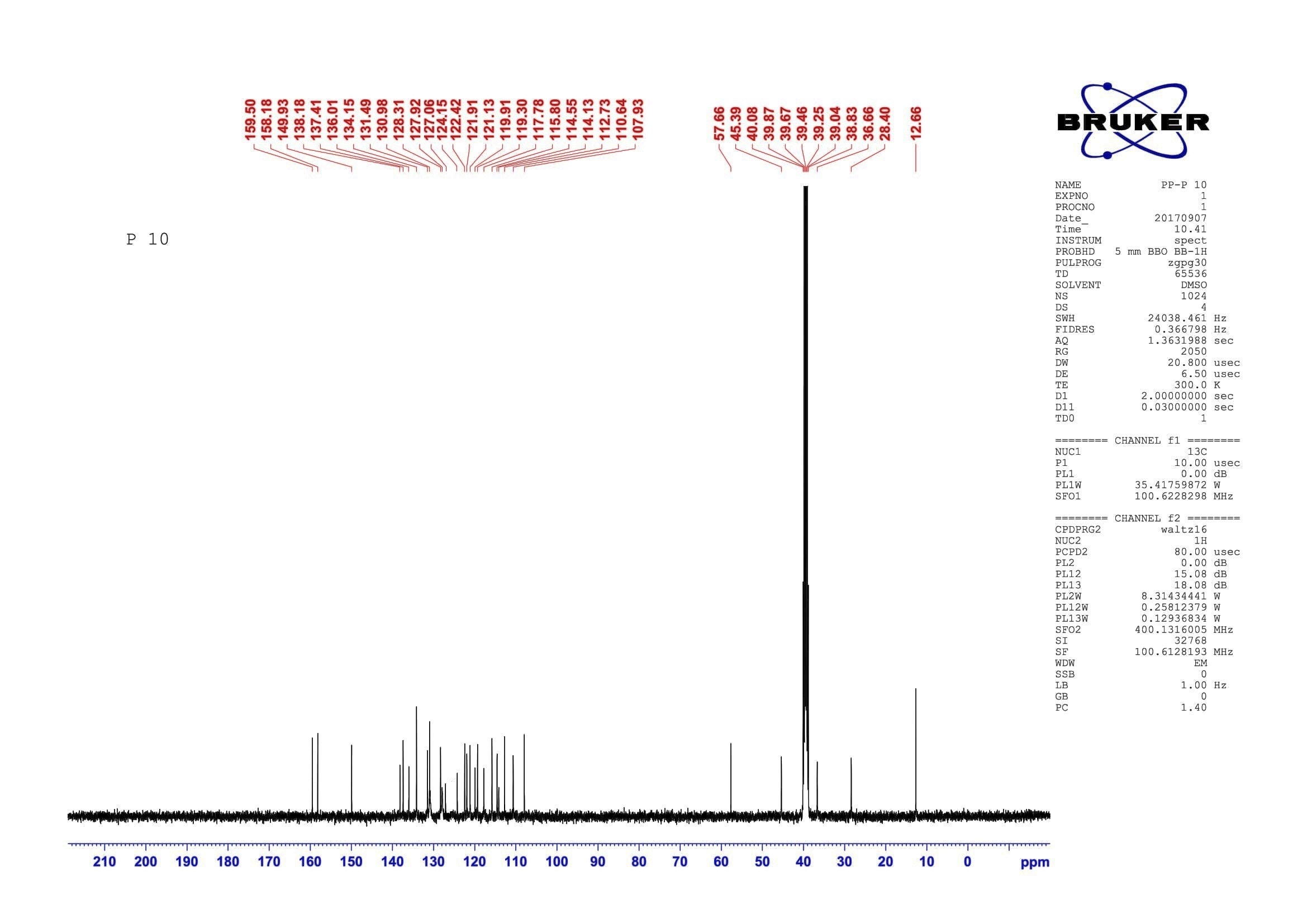
**

^13^C NMR spectra of compound **P_11_**


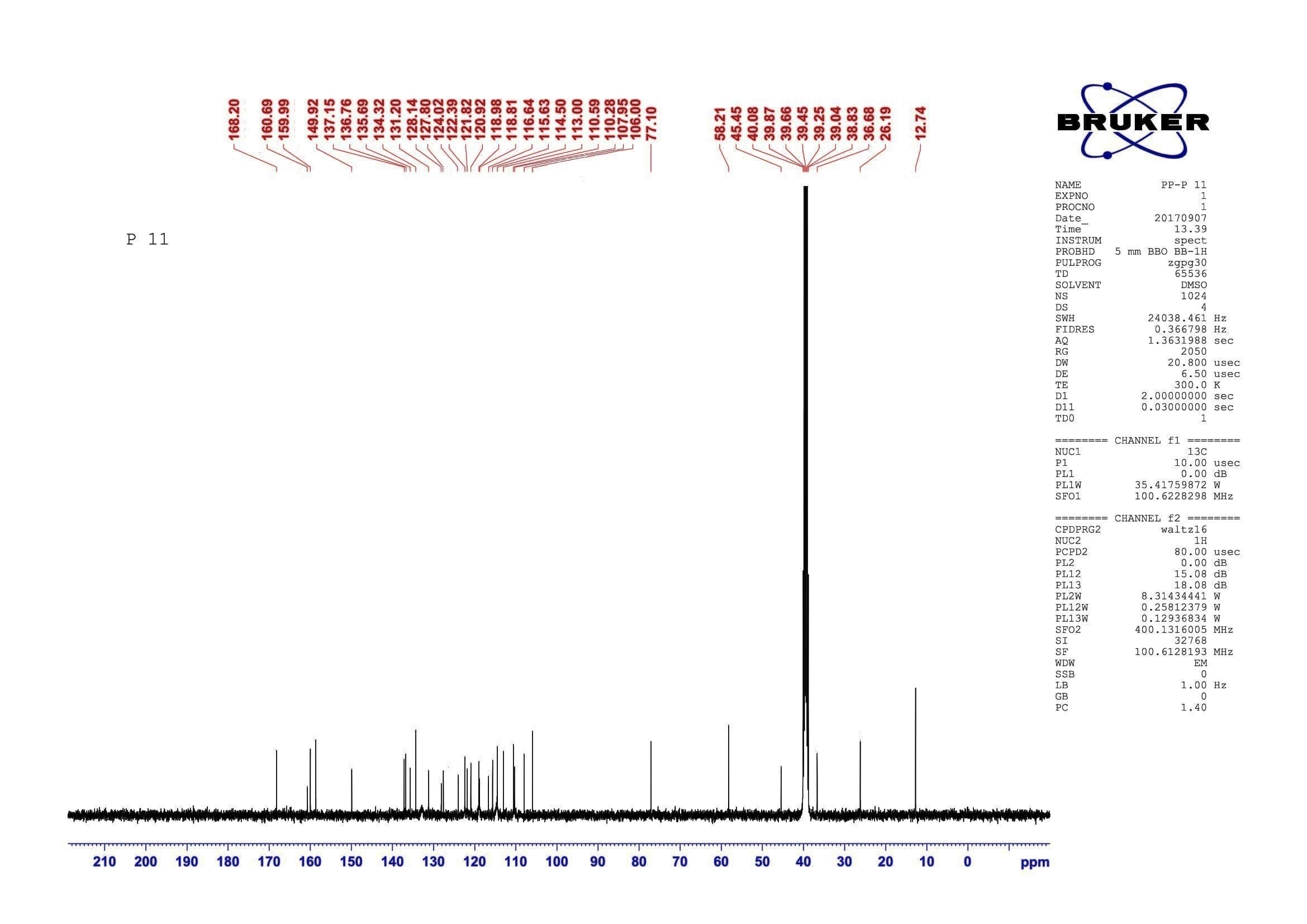


^13^C NMR spectra of compound **P_12_**


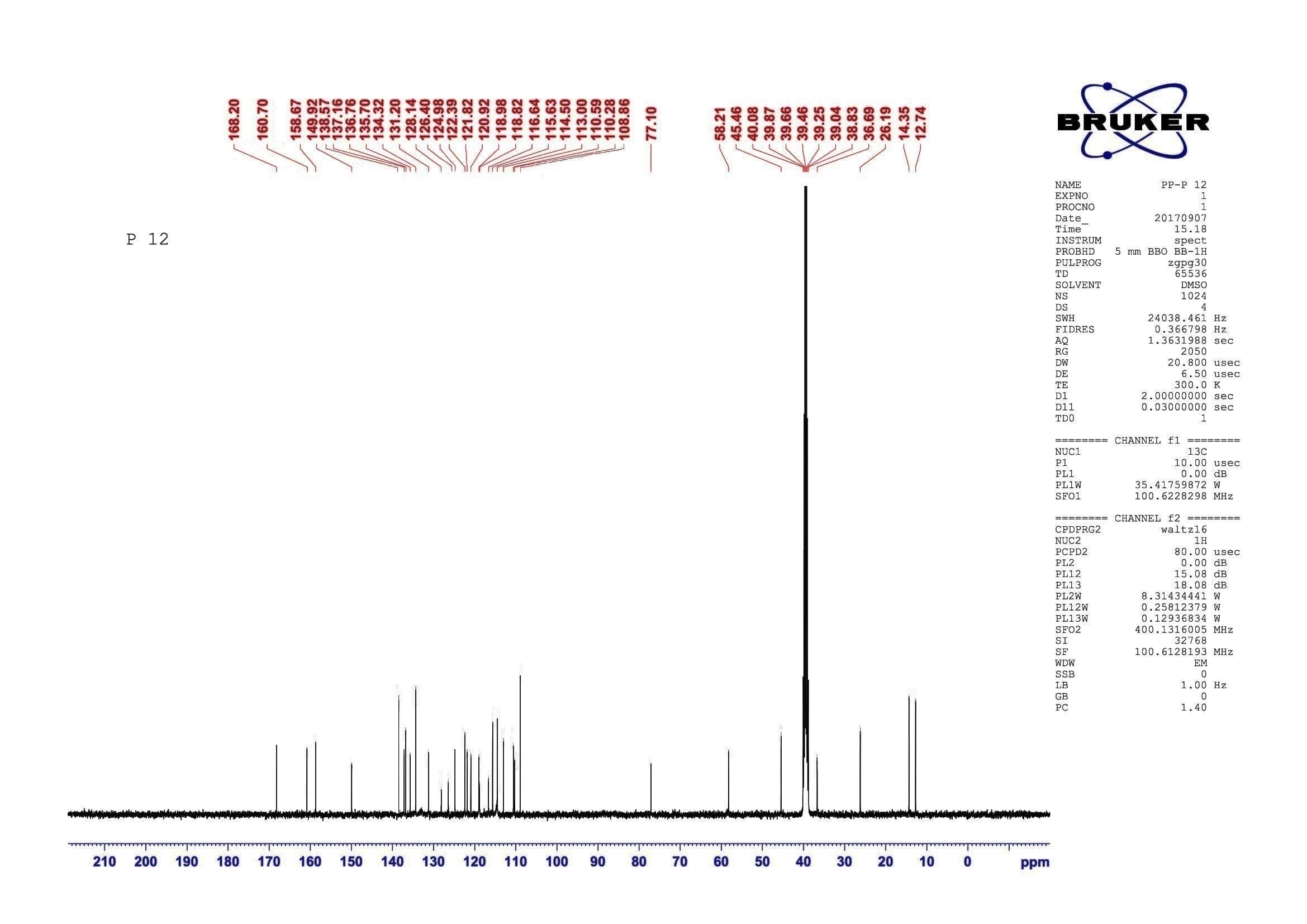


^13^C NMR spectra of compound **P_13_**


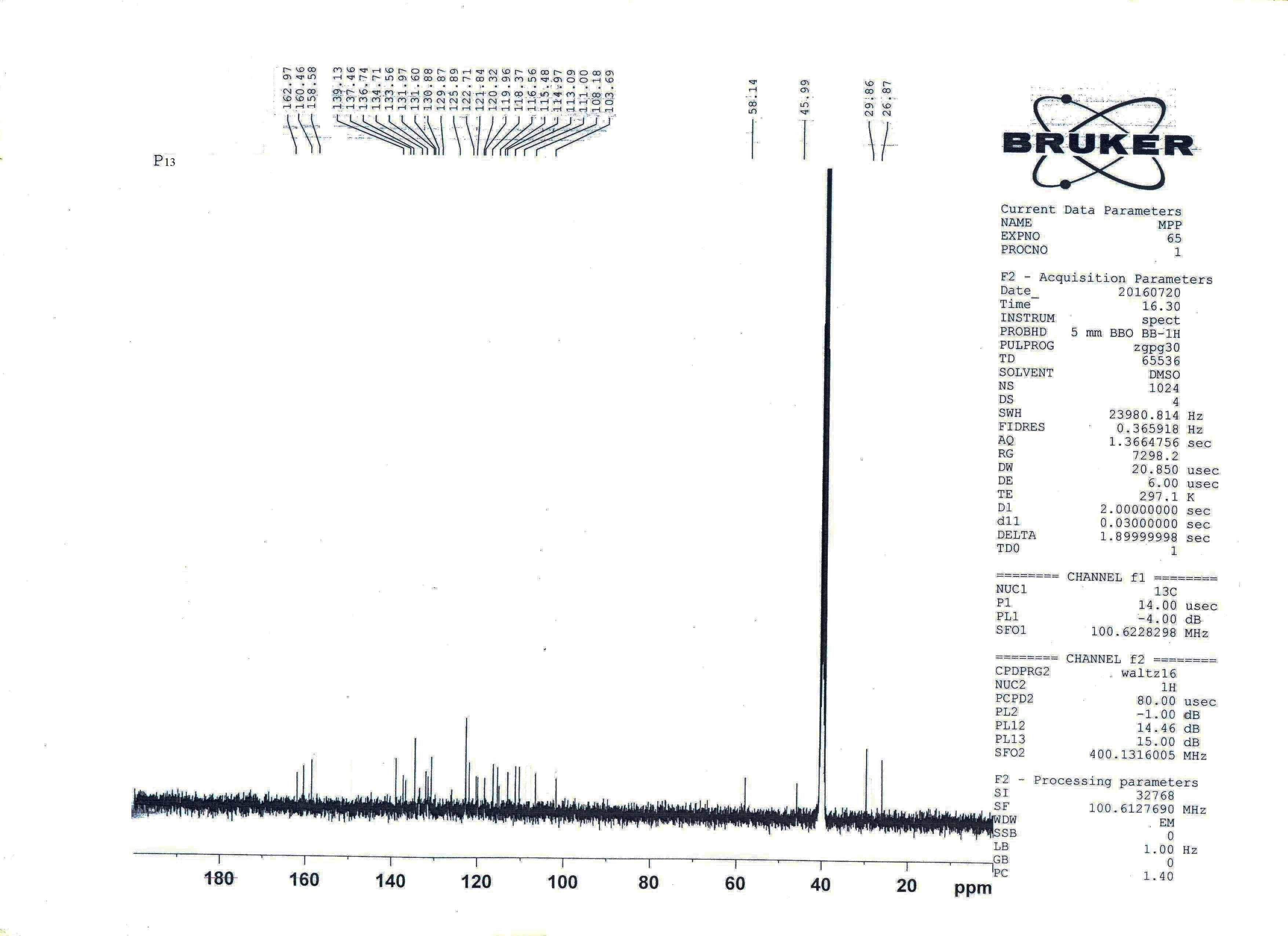


^13^C NMR spectra of compound **P_14_**


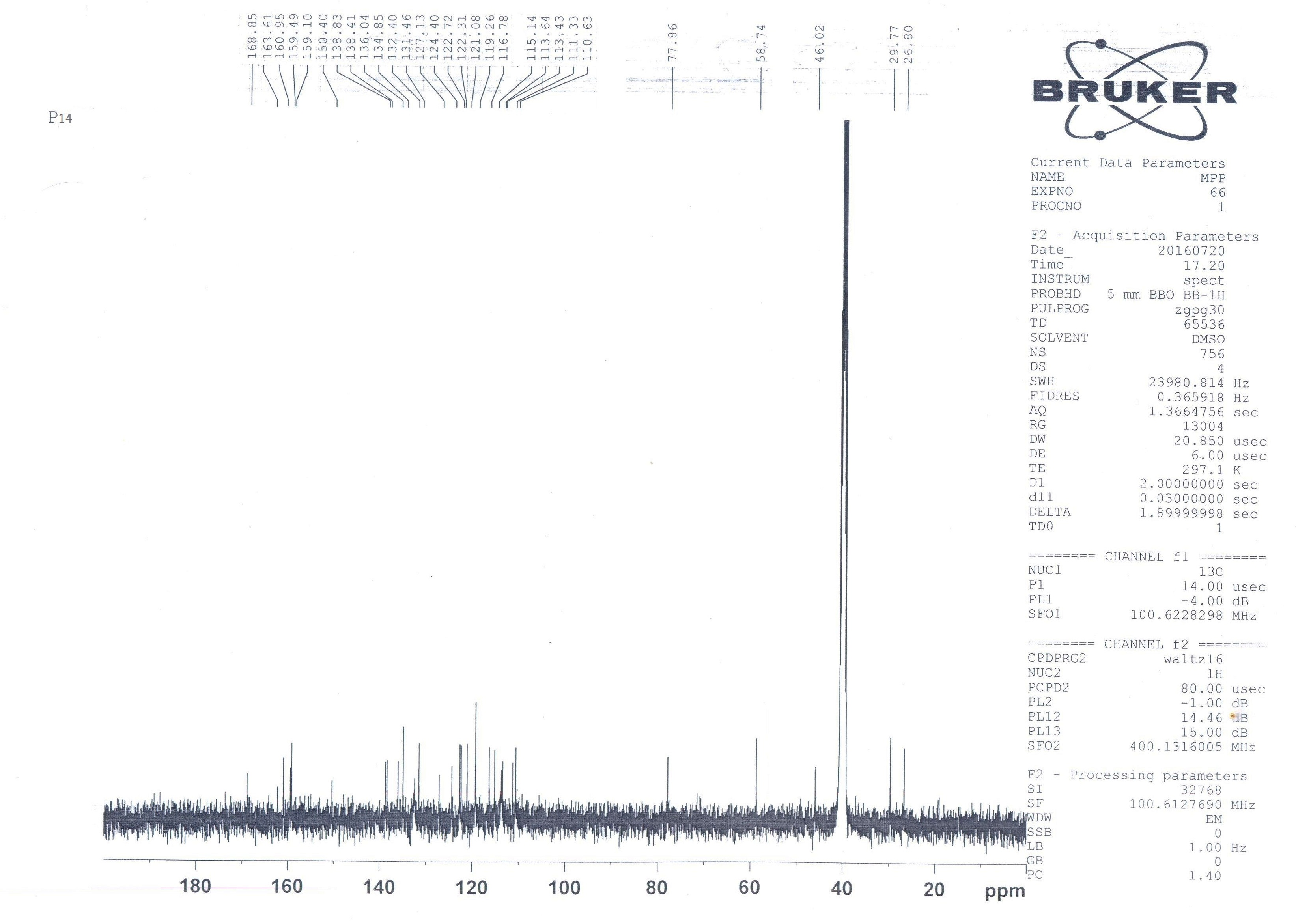


^13^C NMR spectra of compound **P_15_**


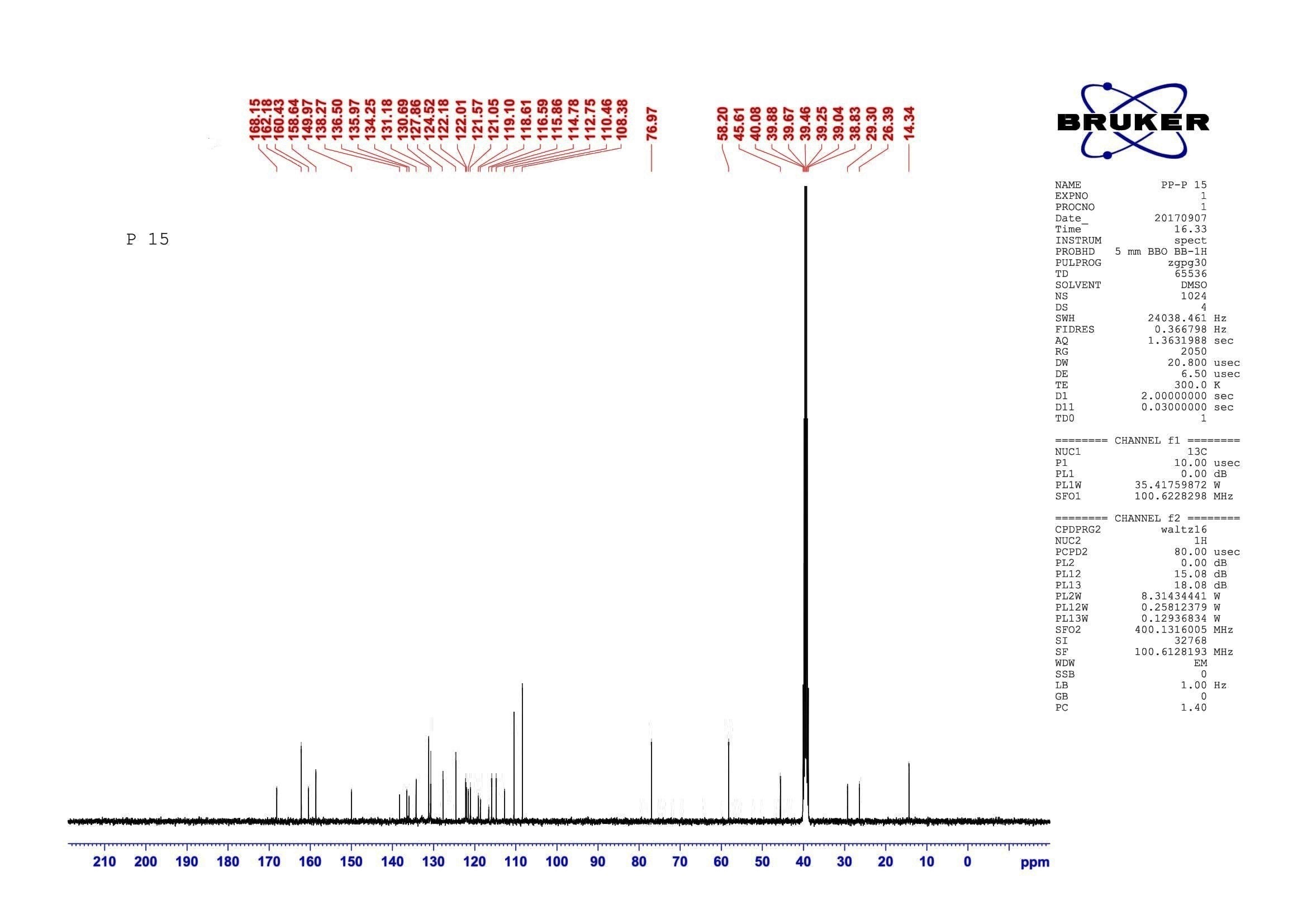


^13^C NMR spectra of compound **P_16_**

**
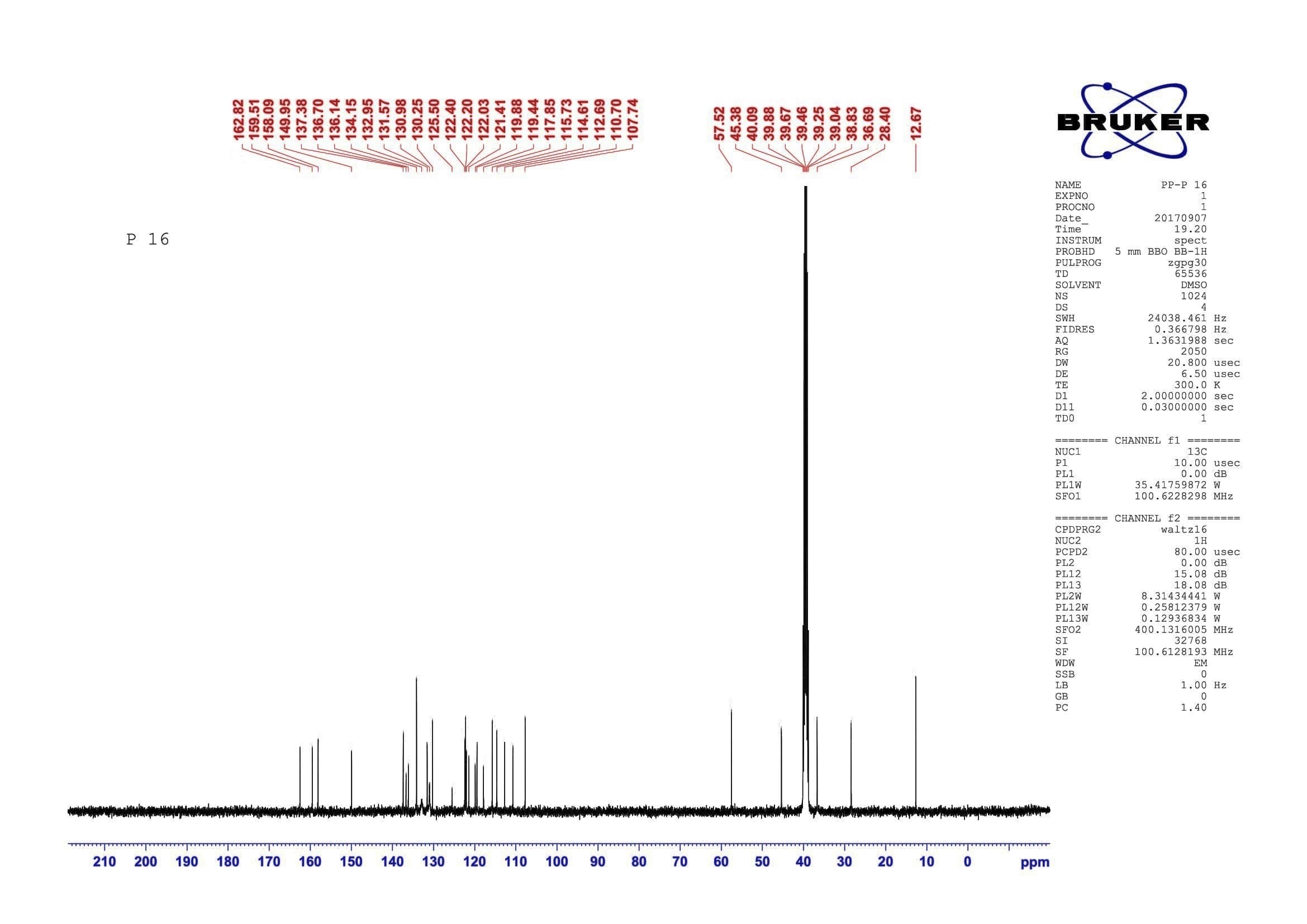
**

^13^C NMR spectra of compound **P_17_**


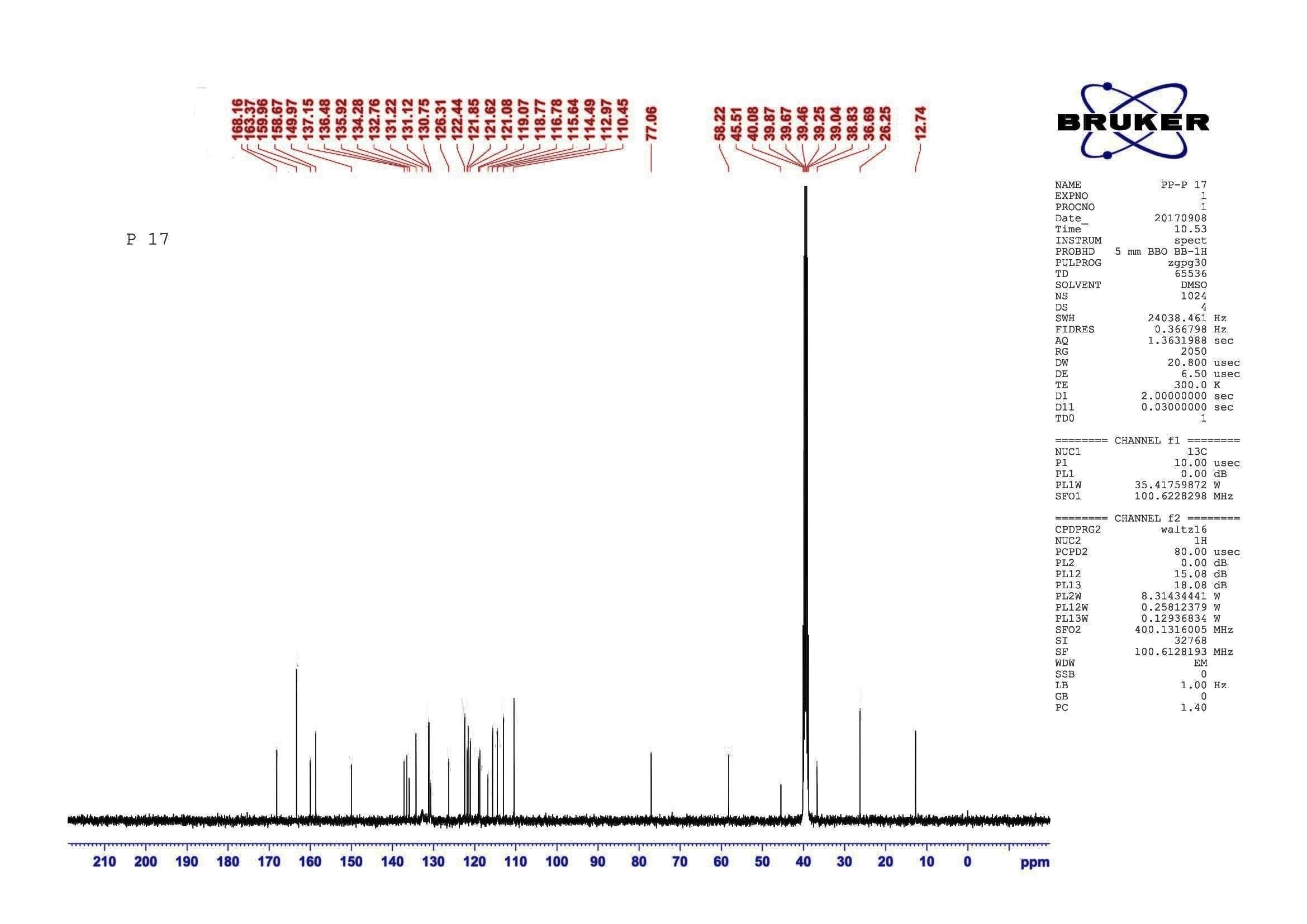


^13^C NMR spectra of compound **P_18_**

**
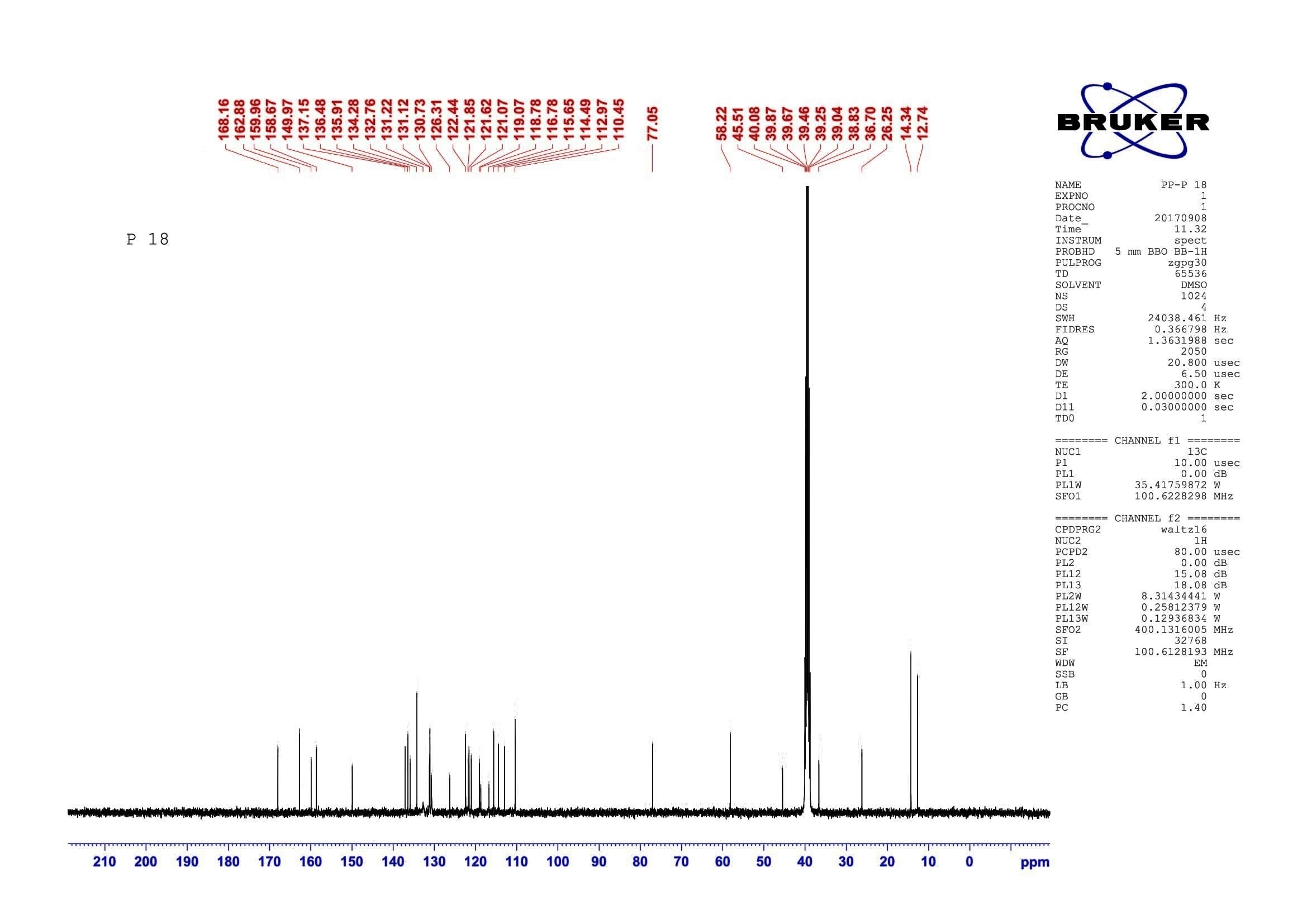
**

^13^C NMR spectra of compound **P_19_**


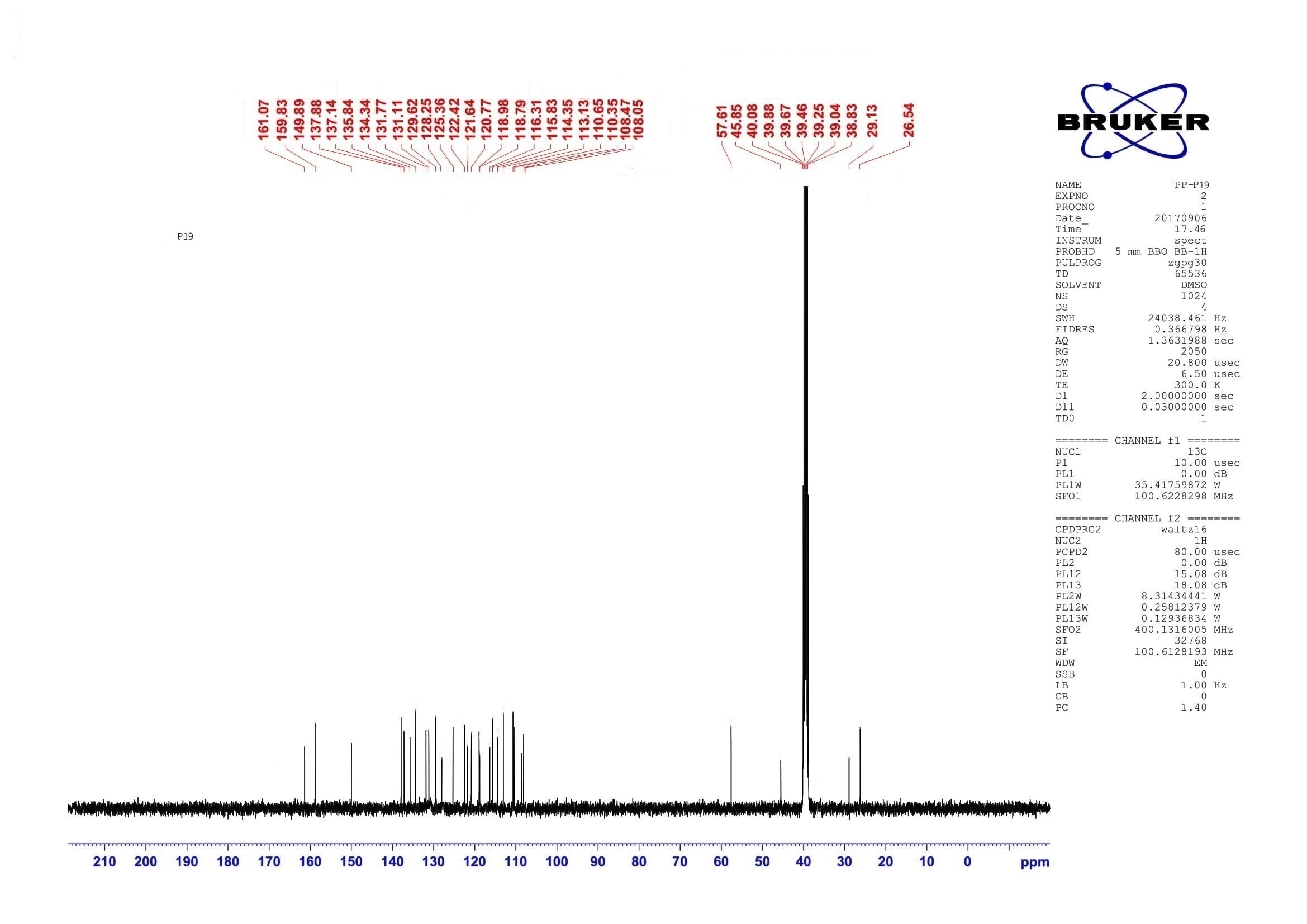


^13^C NMR spectra of compound **P_20_**


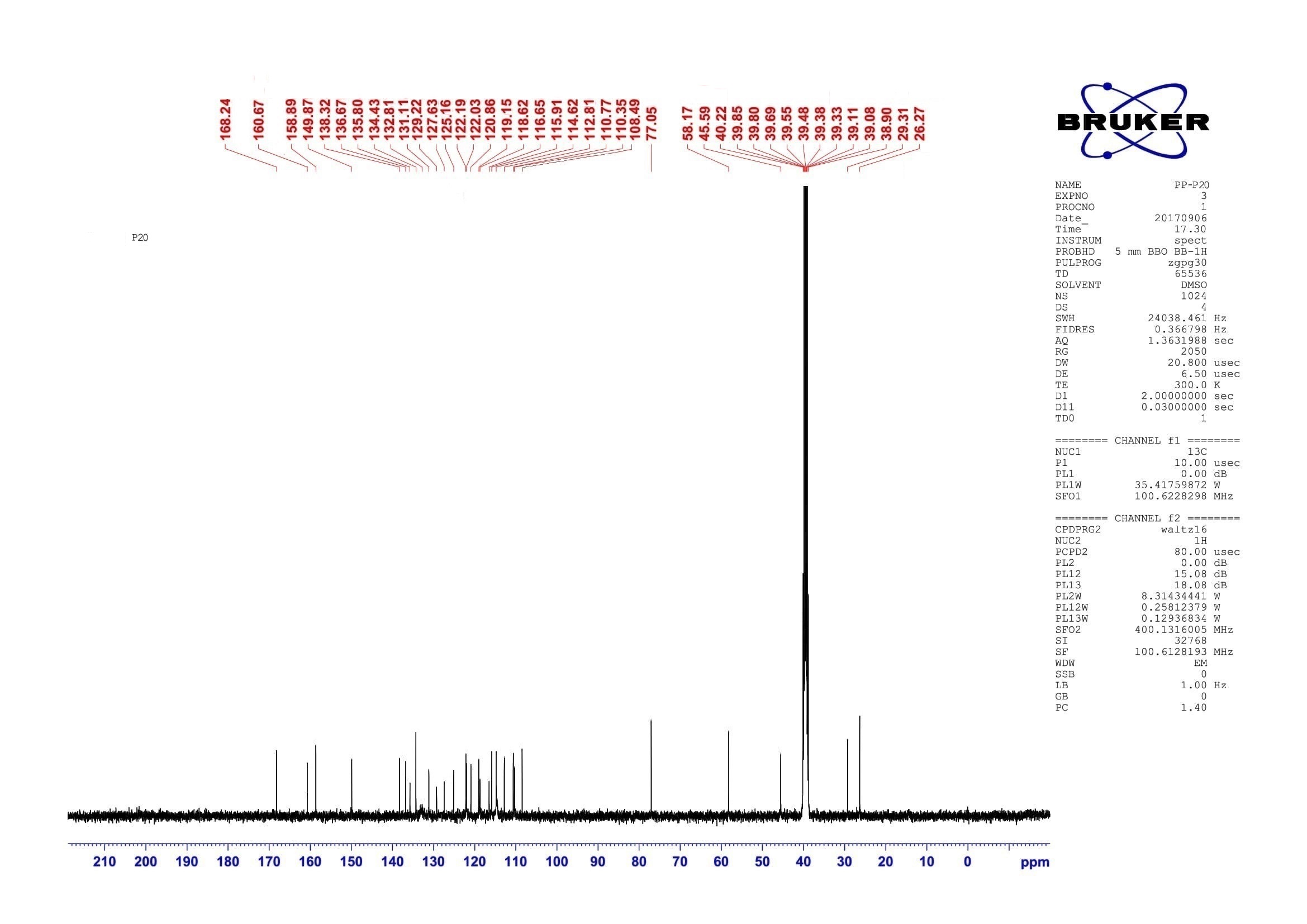


^13^C NMR spectra of compound **P_21_**


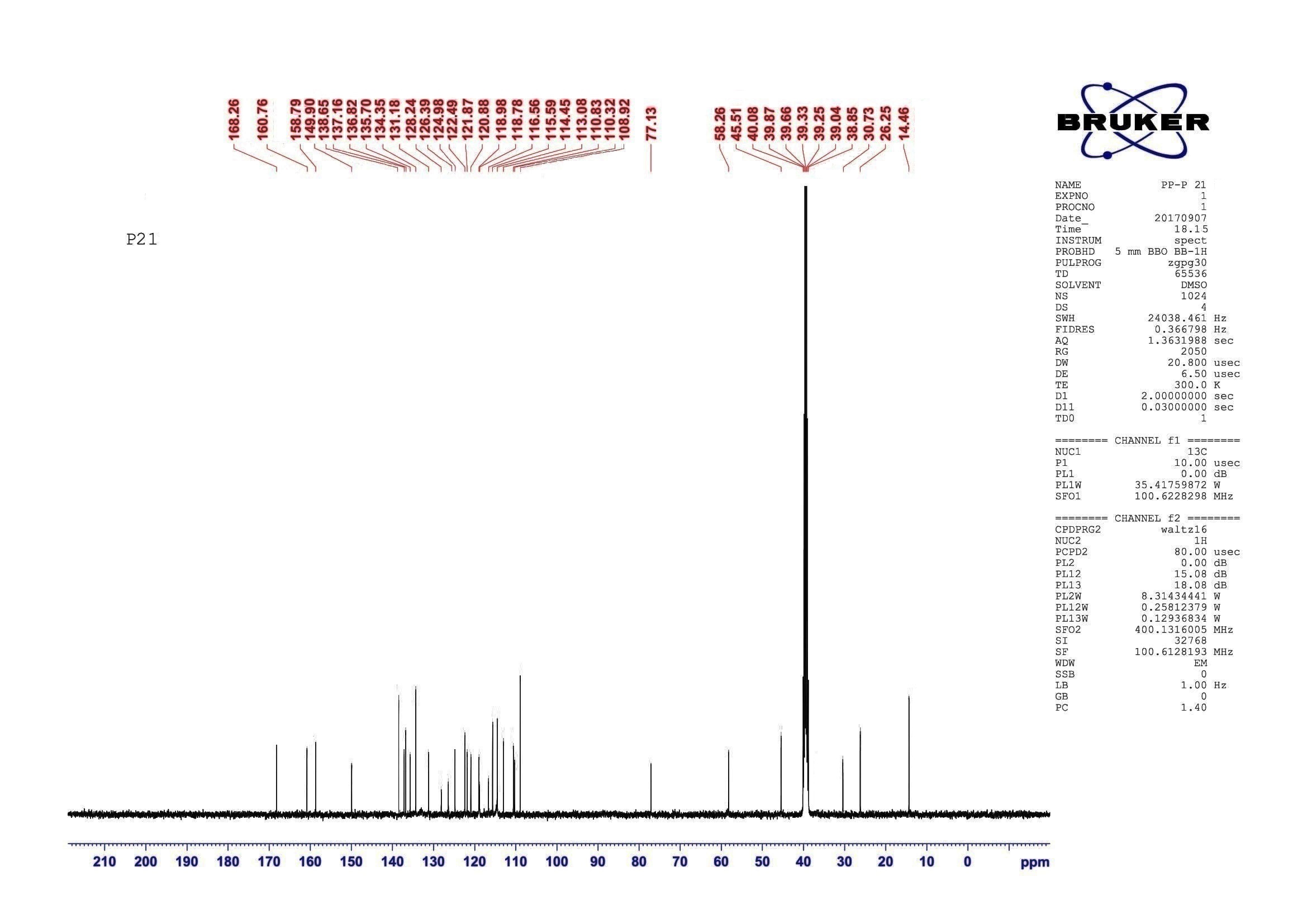


^13^C NMR spectra of compound **P_22_**

**
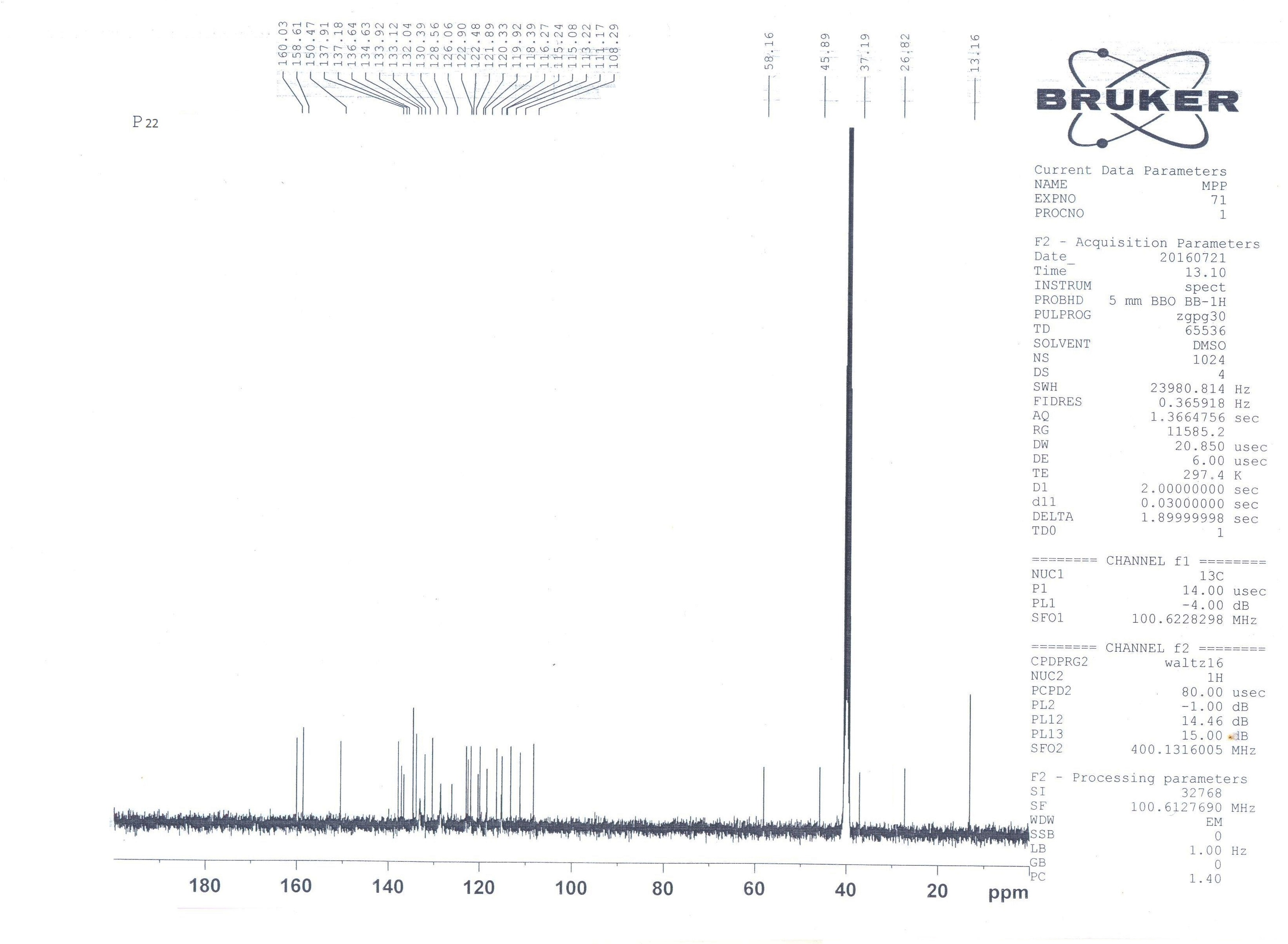
**

^13^C NMR spectra of compound **P_23_**

**
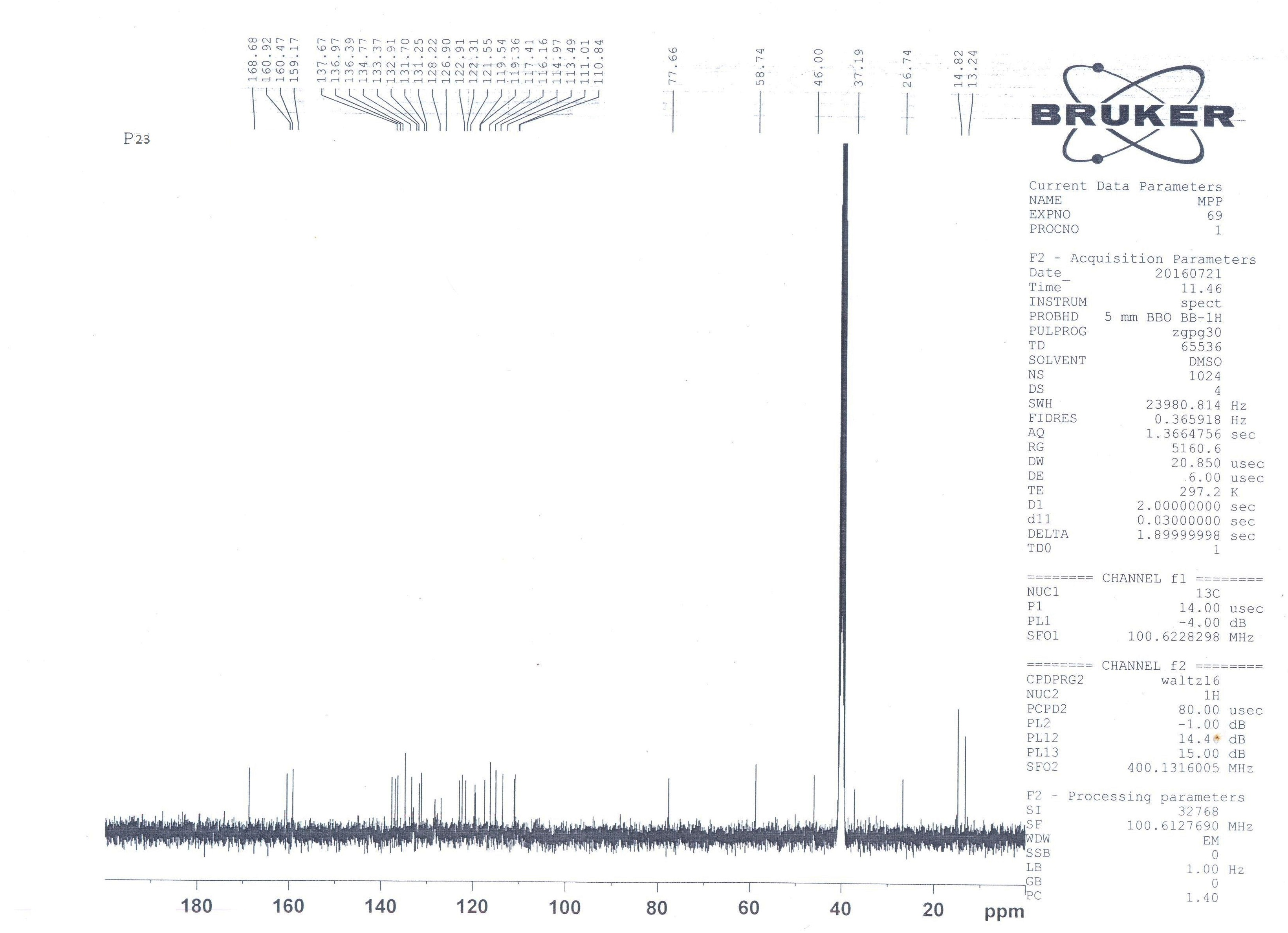
**

Mass spectra of compound **P_5_**

**
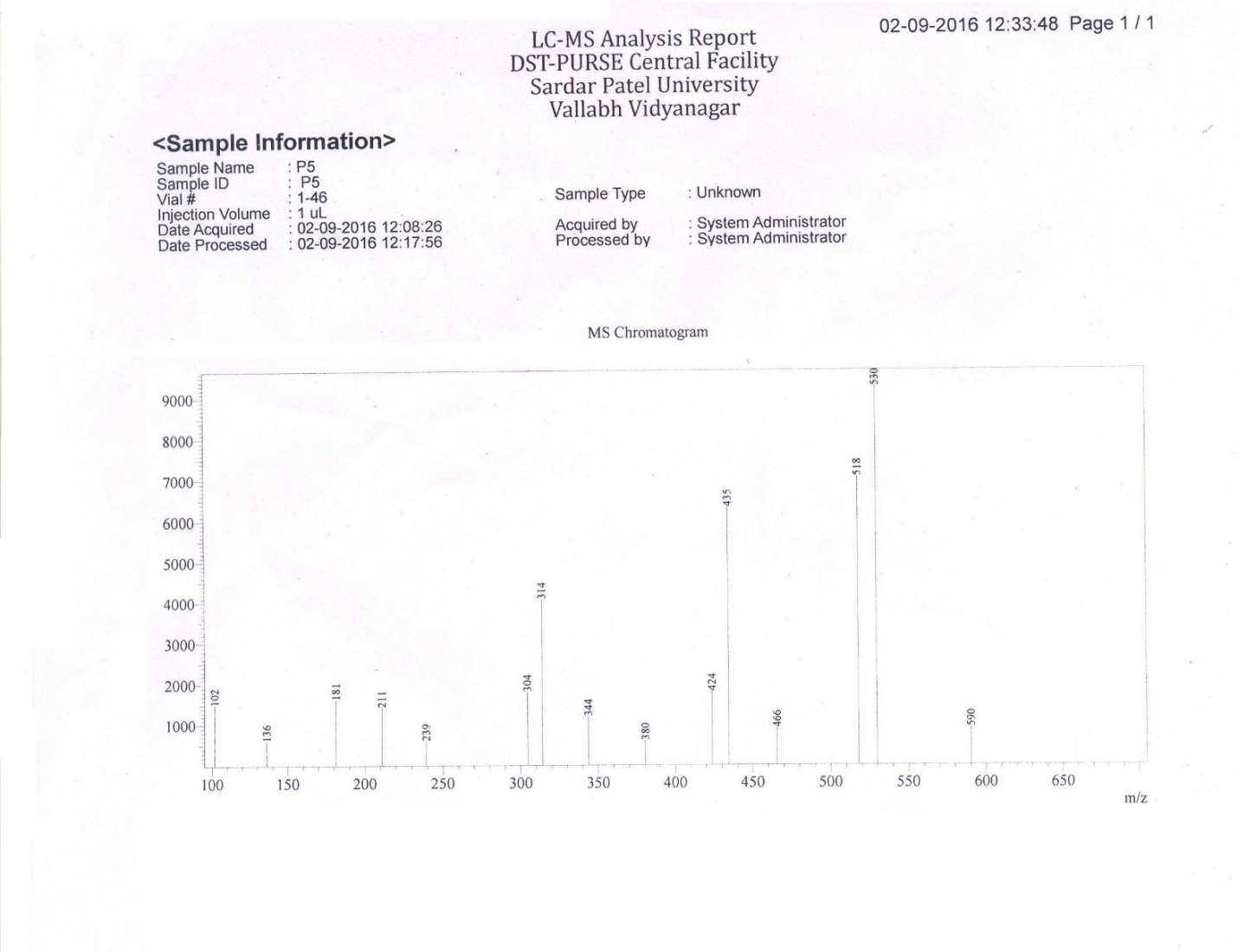
**

Mass spectra of compound **P_14_**

**
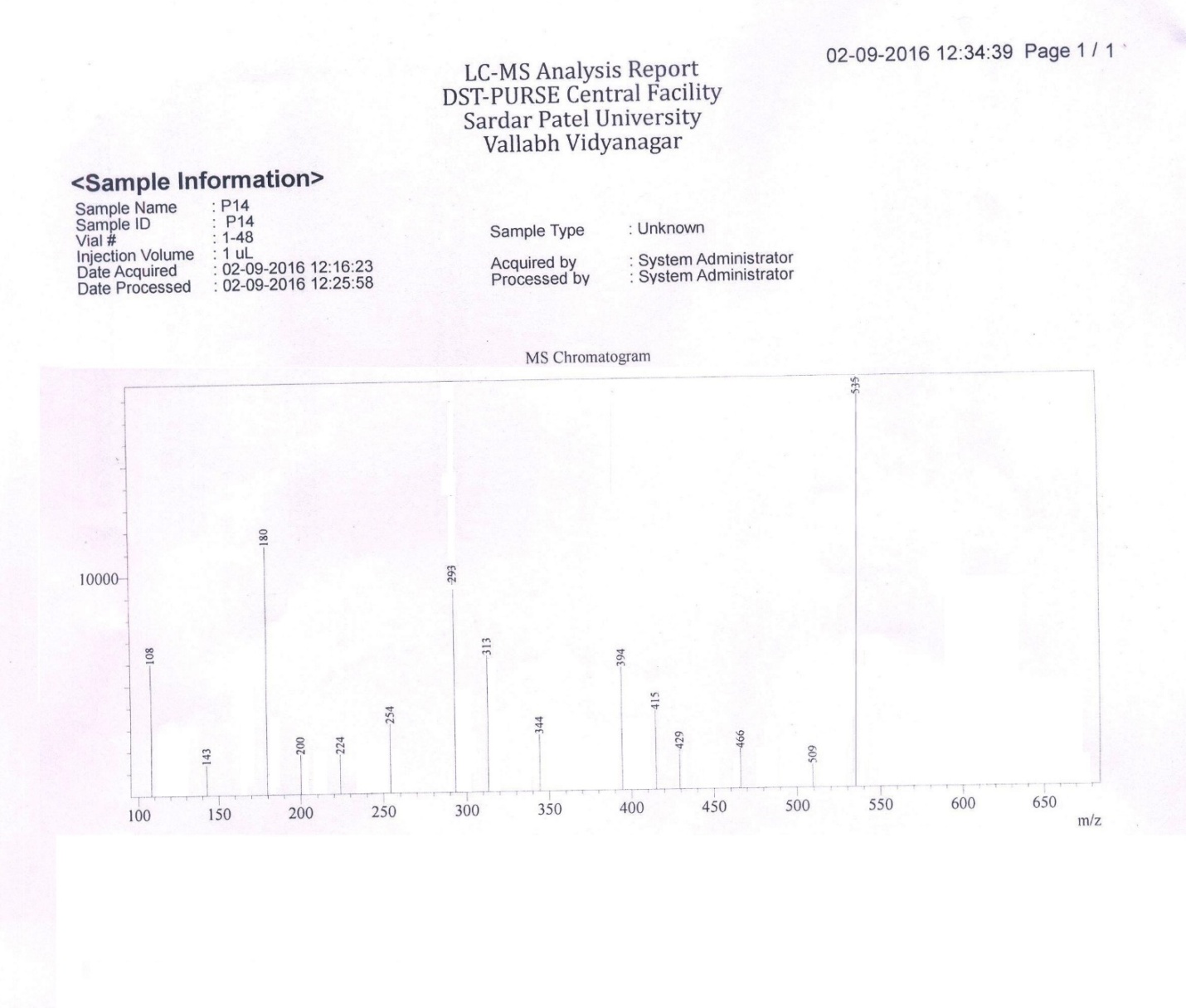
**

Mass spectra of compound **P_17_**

**
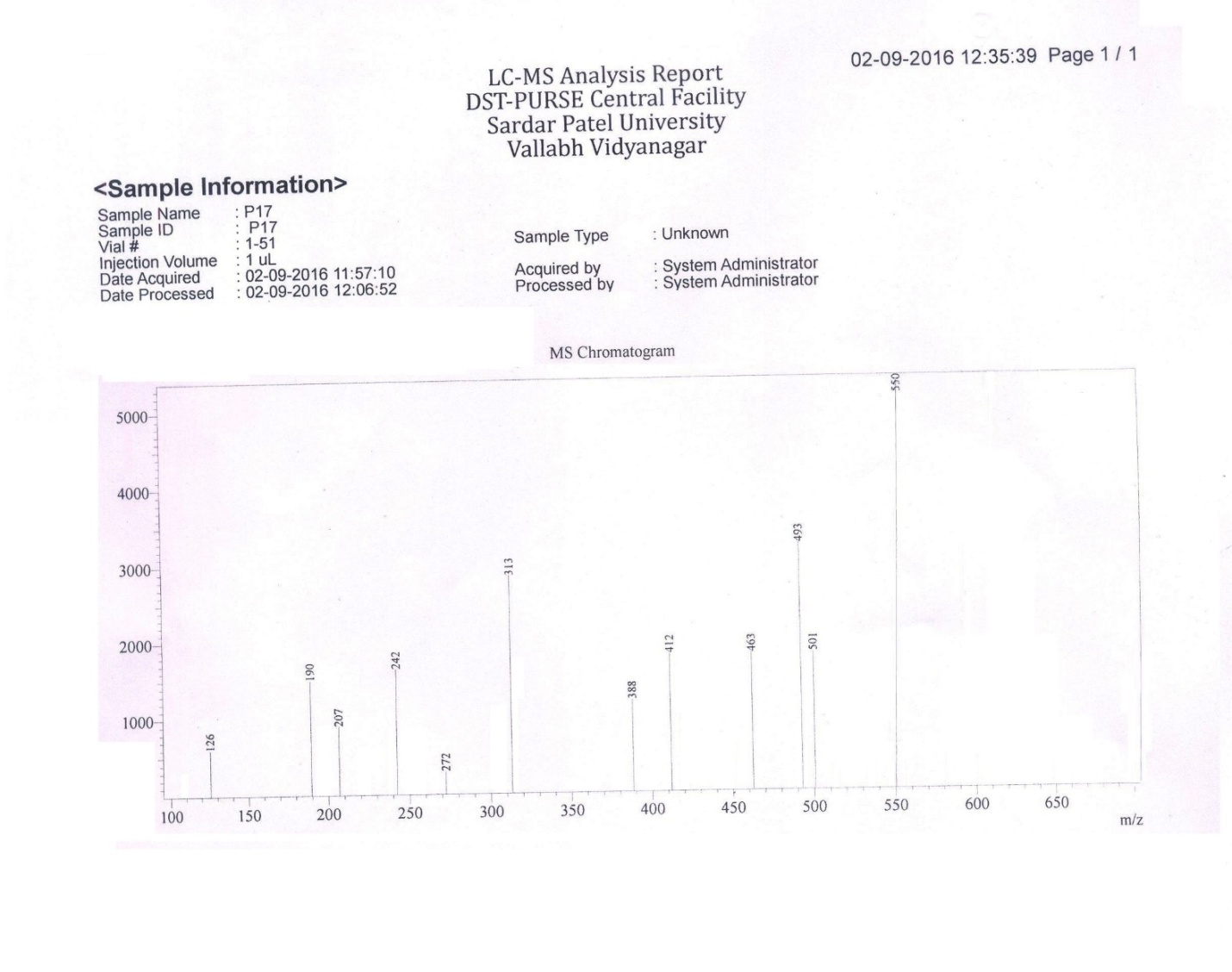
**
